# TCM visualizes trajectories and cell populations from single cell data

**DOI:** 10.1038/s41467-018-05112-9

**Published:** 2018-07-16

**Authors:** Wuming Gong, Il-Youp Kwak, Naoko Koyano-Nakagawa, Wei Pan, Daniel J. Garry

**Affiliations:** 1 Lillehei Heart Institute, University of Minnesota, 2231 6th St S.E, 4-165 CCRB, Minneapolis, MN 55114 USA; 2 Division of Biostatistics, School of Public Health, University of Minnesota, 420 Delaware St. S.E., Mayo Bldg. A302, Minneapolis, MN 55455 USA

## Abstract

Profiling single cell gene expression data over specified time periods are increasingly applied to the study of complex developmental processes. Here, we describe a novel prototype-based dimension reduction method to visualize high throughput temporal expression data for single cell analyses. Our software preserves the global developmental trajectories over a specified time course, and it also identifies subpopulations of cells within each time point demonstrating superior visualization performance over six commonly used methods.

## Abstract (web summary)

Time series single cell expression data has large variance between time points and is challenging for analysis. Here, the authors develop a new dimension reduction and data visualization tool for large scale temporal scRNA-seq data which identifies trajectories and subpopulations.

## Introduction

Single cell expression analyses such as single cell RNA-seq (scRNA-seq) and single cell PCR (scPCR) provide unprecedented opportunities to study the complex cellular dynamics during various developmental processes^1–6^, stem cell differentiation^7,8^, reprogramming^9^ and stress responses^10^. Because of the heterogeneity of the single cell data due to the stochastic nature of gene expression at the single cell level^8,11^, asynchronized cellular programs^12,13^ and technical limitations^14^, the high dimensional expression profiles are initially examined on two dimensional latent space in the form of an *x*-*y* scatter plot.

Diffusion map^6^ and t-Distributed Stochastic Neighbor Embedding (t-SNE)^15^ are among the most popular dimension reduction methods for single cell analyses. Diffusion map, as well as similar methods such as Principal Component Analysis (PCA), captures the major variance from the expression profiles and is suitable for reconstructing the global developmental trajectories, while t-SNE focuses on the definition and discovery of subpopulations of cells. Additional methods such as diffusion pseudotime^16^, Wishbone^17^, Monocle^8^ and TSCAN^12^ are based upon the high dimensional information embedded within the two dimensional scatter plot.

The time series expression data are usually characterized by large variance between time points during the developmental program. Therefore, cells from the same time points tend to cluster together on the latent spaces produced by diffusion map and t-SNE. The subpopulations of cells within each time point are usually indistinguishable, due to minor expression differences compared with the more dominant temporal differences. Thus, there is a need for an efficient algorithm to visually inspect large-scale temporal expression data on a single two-dimensional latent space that preserves the global developmental trajectories and separates subpopulations of cells within each developmental stage.

Here, we develop a dimension reduction and data visualization tool for temporal single cell expression data, which we name Topographic Cell Map (TCM). We demonstrate that TCM preserves the global developmental trajectories over a specified time course, and identifies subpopulations of cells within each time point. We provide the R implementation of TCM as a Supplementary Software Program.

## Results

### TCM is a novel prototype-based dimension reduction algorithm

**Fig. 1:**
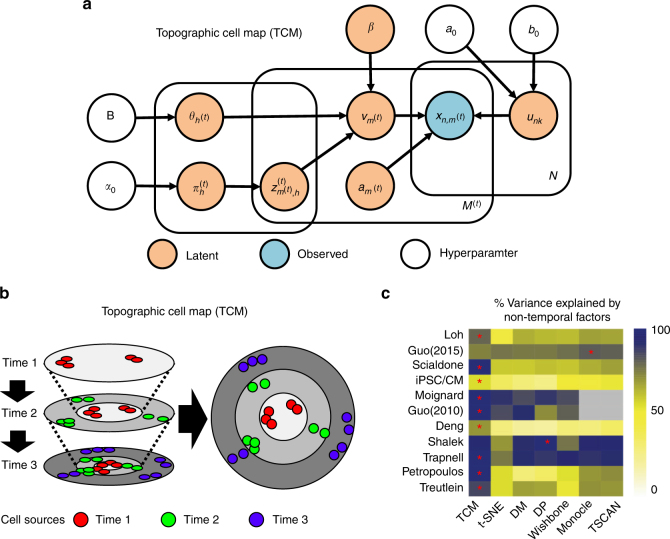
TCM reduces the variance due to temporal factors on the latent space**. a** Graphical model representation of TCM. The boxes are “plates” representing replicates. The left plate represents prototypes, the middle plate represents cells and the right plate represents genes. **b** In TCM, the cells from each time point are simultaneously mapped to multiple time point specific latent spaces, preventing the cells from the same time points crowding together due to the high temporal variance usually present in the time series expression datasets. To reconstruct the global developmental trajectories, the time point specific latent spaces are convolved together to produce a single latent space where cells from early and late time points distribute at the center and periphery, respectively. **c** The heatmap indicates the percent of variance explained by non-temporal factors on the two dimensional latent space produced by TCM, t-SNE, diffusion map (DM), diffusion pseudotime (DP), Wishbone, Monocle, and TSCAN on 11 examined single cell expression datasets. The lower percentage suggests the latent space is more dominated by the temporal variance. The red asterisk indicates the method that provides the highest percent of variance explained by non-temporal factors

TCM is a Bayesian generative model that is optimized using a variational expectation-maximization (EM) algorithm (Fig. 1a). TCM approximates the gene-cell expression matrix by the product of two low rank matrices: the metagene basis that characterizes gene-wise information and metagene coefficients that encode the cell-wise features. The cells represented as Gaussian metagene coefficients are mapped to a low-dimensional latent space in a similar fashion as non-linear latent variable models such as generative topographic mapping (GTM)^18^. To prevent a single latent space from being dominated by temporal variances, cells from different developmental stages are simultaneously mapped to multiple time point specific latent spaces, so that the subpopulations within each time period or developmental stage can be revealed on their individual latent spaces. To reconstruct the global developmental trajectories, the time point specific latent spaces are convolved together to produce a single latent space where the cells from early time points or developmental stages are located at the center and the cells from the later time points or developmental stages are located at the peripheral area (Fig. 1b and Supplementary Fig. 1).

**Fig. 2:**
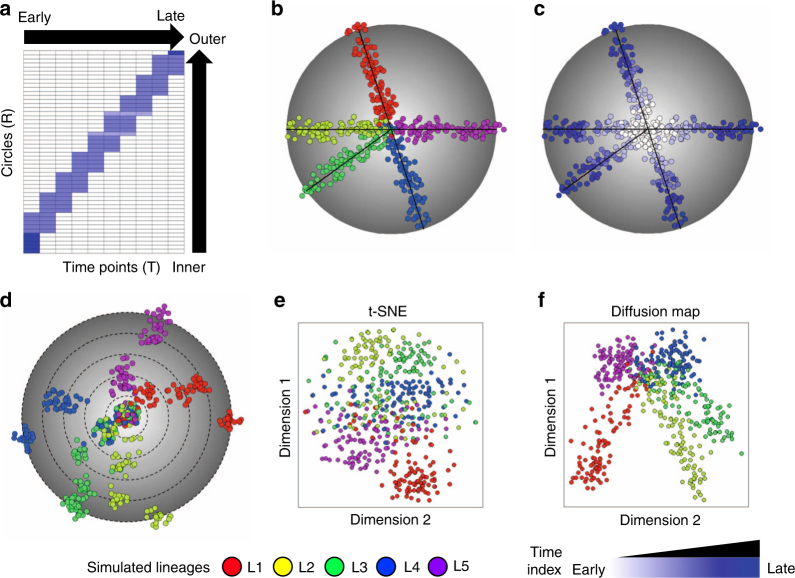
TCM has improved performance for the detection of subpopulations of cells in simulation study**. a** The heatmap shows the sampling probabilities for the sequential differentiation models. In the sequential cell sampling, the sampling time is positively correlated to the developmental speed. **b-c** The simulated temporal scRNA-seq datasets with five lineages under sequential differentiation models (*N* = 2000 genes and *M* = 500 cells, with an exponential decay model for the dropout noise), with the color indicating (**b**) the cell lineages or (**c**) time index. **d** TCM was able to successfully reveal the lineage trajectories for the sequential differentiation models. **e-f** The visualization of simulated temporal scRNA-seq datasets under three differentiation models by (**e**) t-SNE and (**f**) diffusion map

First, we systematically examined the performance of TCM on synthetic temporal scRNA-seq datasets with synchronized and two types of asynchronized developmental processes (forward and delayed differentiation models) with multiple (from two to ten) lineages (Fig. 2 and Supplementary Fig. 2). We found that TCM successfully revealed the lineage trajectories and had the best performance of cell separation from different lineages compared to other tested methods, such as t-SNE and diffusion map, under various conditions (see Supplementary Note 1 for the details of simulating the temporal scRNA-seq dataset, Supplementary Note 2 for three cell differentiation models, and Supplementary Note 3 for evaluation of the performance of TCM on four types of synthetic temporal scRNA-seq datasets; Supplementary Figs. 2 and 3). We also observed that TCM had decreased generation of artificial branches on homogenous scRNA-seq datasets with random time index and temporal scRNA-seq datasets with a single lineage (Supplementary Fig. 3).

### TCM preserves the global developmental trajectories

**Fig. 3:**
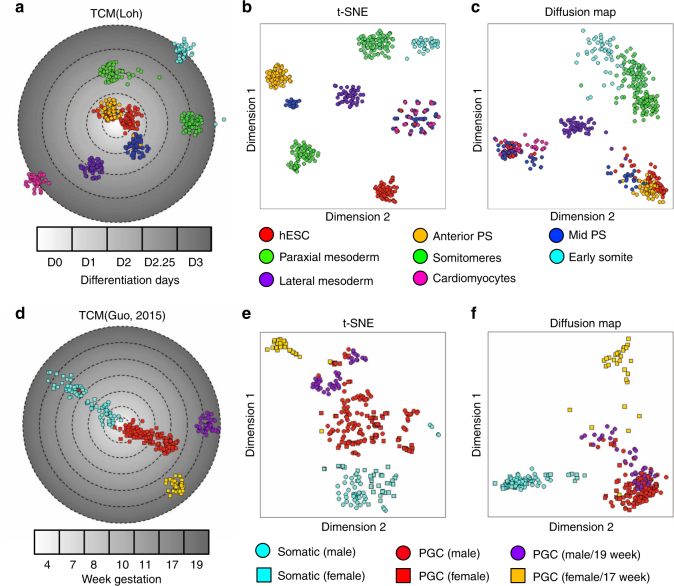
TCM preserves the global developmental trajectories for the visualization of temporal single cell expression data. **a-c** TCM shows superior performance pertaining to the discovery of two major lineages of anterior and mid primitive streak (PS), and separating individual subpopulations compared to (**c**) t-SNE and (**d**) diffusion map on the visualization of a scRNA-seq dataset of hESC derived mesodermal lineages. **d**–**f** TCM shows superior performance compared to (**e**) t-SNE and (**f**) diffusion map on the reconstruction of the bifurcation of somatic and primordial germ cells (PGCs), and the female (17 weeks after gestation) and male (19 weeks after gestation) PGCs on a temporal scRNA-seq dataset of human somatic cell and PGC development from weeks 4 to 19 after gestation

Next, we compared the performance of the visualization of 11 temporal single cell expression datasets between TCM and six other algorithms. We found that TCM produced latent spaces with a significantly higher percent of variance explained by non-temporal factors compared to six commonly used tools on nine of 11 datasets (Fig. 1c and Supplementary Fig. 4a). To examine the capability of TCM to preserve the global developmental trajectories, we performed the TCM analysis on a recently published scRNA-seq dataset from human embryonic stem cell (hESC)-derived mesodermal lineages^7^. In this study, human ESCs (day 0) were initially differentiated into two distinct lineage paths: the anterior and mid-primitive streak (PS) (day 1). The anterior PS then differentiated into paraxial mesoderm (day 2), somitomeres (day 2.25), and early somites (day 3), while the mid-PS differentiated into lateral mesoderm (day 2), followed by cardiomyocytes (day 3). TCM successfully revealed the bifurcation of two major mesodermal lineages toward somites (outer circle, cyan dots) and cardiomyocytes (outer circle, pink dots) (Fig. 3a). In contrast, t-SNE and Wishbone failed to distinguish the trajectories of two mesodermal lineages (Fig. 3b and Supplementary Fig. 5b). The diffusion map, as well as diffusion pseudotime, Monocle and TSCAN, on the other hand, failed to separate more than 60% of the subpopulations of cells (e.g., hESCs, anterior PS, and mid PS), although the bifurcation of the two mesodermal lineages was generally recovered (Fig. 3c and Supplementary Fig. 5a, c and d).

As another example, we compared the performance of defining developmental trajectories of human primordial germ cells (PGC) and neighboring somatic cells from weeks 4 to 19 post-gestation datasets^4^. TCM clearly identified two major lineages of somatic cell and PGC development and a bifurcation of female (17 weeks) and male (19 weeks) PGCs (Fig. 1d). In contrast, other tools did not preserve the bifurcation of female and male PGCs or resolve the majority of somatic cells and PGCs from weeks 7 to 11, as well as part of the male week 19 PGCs (Fig. 1e, f and Supplementary Fig. 6a–d).

### TCM identifies subpopulations of cells within each time point

**Fig. 4:**
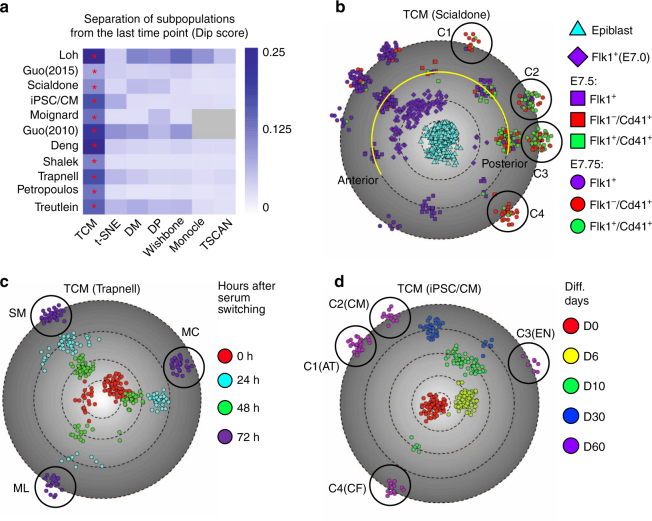
TCM identifies subpopulations of cells from the last time points for the visualizing of temporal single cell expression data. **a** The heatmap indicates the capability of separating subpopulations from the last time point on the two dimensional latent space produced by TCM, t-SNE, diffusion map (DM), diffusion pseudotime (DP), Wishbone, Monocle, and TSCAN on 11 examined single cell expression datasets. The performance is quantitatively measured by Hartigan’s Dip statistics using the cells’ coordinates on the latent space. The high Dip score suggests the cells from the last time point are separated to a greater extent on the latent space. The red asterisk indicates the method that provides the highest Dip score. **b** TCM is used to visualize the scRNA-seq dataset of mouse mesodermal diversification. The principal anterior-posterior axis is highlighted along the single cells captured at E7.5. TCM identifies four hematopoietic (Cd41^+^, red circle Flk1^−^/Cd41^+^, and green circle Flk1^+^/Cd41^+^) subpopulations from E7.75 cells (C1-C4). **c** TCM is used to visualize the single cell RNA-seq data following the differentiation of human primary myoblasts, where the expression pattern of 372 single cells were profiled from 0, 24, 48, and 72 h post-serum switching, respectively. TCM successfully identifies three distinct subpopulations: skeletal muscle (SM), interstitial mesenchymal cells (MC) and myocyte-like cells (ML) from the last time point (72 h). **d** TCM is used to visualize the scRNA-seq dataset of human induced pluripotent stem cells (hiPSCs) to cardiomyocytes (CMs) differentiation, where the single cell transcriptomes were profiled at days 0, 6, 10, 30, and 60 following differentiation. TCM identifies four terminal subpopulations of cells from day 60 (C1-C4)

Time series single cell expression analysis is usually utilized to study dynamic biological process where the cells from the later time points (or later developmental stages) demonstrate increased heterogeneity than the earlier ones. We found that TCM has consistently significantly better performance with the separation of the subpopulations form the final or last time point on all 11 datasets, as measured by the Hartigan’s Dip statistics of the cells’ distribution on the latent space (Fig. 4a and Supplementary Fig. 7)^19^.

On three single cell expression datasets from mouse and human preimplantation embryonic development^2,3,5^, we verified the capability of TCM to define the bifurcation of the inner cell mass (ICM) and trophectoderm (TE) from the blastocyst stage, while other algorithms were unable to separate the ICM and TE populations on two scRNA-seq datasets (Supplementary Figs. 8–10).

On scRNA-seq dataset of mouse mesodermal diversification^1^, TCM not only identified multiple populations from E7.5 Flk1^+^, Flk1^−^/Cd41^+^, and Flk1^+^/Cd41^+^ cells along the principal anterior/posterior axis, but also identified four distinct hematopoietic subpopulations (Cd41^+^ cells) from E7.75 cells (Fig. 4b and Supplementary Fig. 11a–c). The C1 population co-expressed genes from multiple lineages: increased expression of mesodermal genes Hand1 and Fgfr1, and decreased expression of Gata1 and Hba-x, suggesting that cells are in progenitor states during hematopoiesis (Supplementary Fig. 11c, d, and g)^20^. C2 and C3 populations were characterized by the strong expression of Zfpm1 (Fog1) and Gata2 that were related to the primitive erythrocyte differentiation (Supplementary Fig. 11e and h)^21^, while hemoglobin genes such as Hbb-bh1 and Hba-x were highly expressed in the C4 population (Supplementary Fig. 11f and i). In contrast, the latent space produced using other algorithms were unable to distinguish these four subpopulations (C1-C4) as they clustered together due to the high temporal variance present in this dataset (Supplementary Fig. 11j–o).

Then, we used TCM to visualize the scRNA-seq dataset of the differentiation of human primary myoblasts, where the expression pattern of 372 single cells were profiled from 0, 24, 48, and 72 h post-serum switching, respectively (Fig. 4c)^1–3,5,6,8,22^. TCM successfully identified three distinct subpopulations: skeletal muscle (SM), interstitial mesenchymal cells (MC) and myocyte-like cells (ML) from the last time point (72 h), as suggested by the expression profiles of known gene markers (Supplementary Fig. 12a–f). In contrast, other algorithms were unable to separate these three subpopulations from the remaining cells, including Monocle, which was used in the original study (Supplementary Fig. 12g–l)^7,8^.

Next, we differentiated human induced pluripotent stem cells (hiPSCs) to cardiomyocytes (CMs) and used TCM to reconstruct the developmental trajectories from the scRNA-seq data of 315 cells captured from hiPSCs and following differentiation (days 6, 10, 30 and 60) (Supplementary Fig. 13). TCM revealed the dynamic changes in gene expression pattern during differentiation (Supplementary Fig. 14a–k) and identified three differentiation trajectories and four subpopulations from 54 single cells at day 60 (post-differentiation) (Fig. 4d). Among them, C1 and C2 populations were characterized by robust expression of mature CM markers such as TNNT2 and MYL2, while some atrial genes such as NPPA and NPPB had higher expression levels in C1 than C2, suggesting diversification of CMs at day 60 (Supplementary Fig. 14d–g, l). On the other hand, C3 and C4 populations represented the minor endothelial and cardio-fibroblast (CF) lineages, supported by the expression of lymphatic endothelial markers such as NR2F2 and AVR1 in the C3 population, CF markers such as CDH11 and CFH in the C4 population, and diminished expression of cardiomyocyte-specific markers (Supplementary Fig. 14h, i, m). In contrast, other algorithms failed to preserve the global developmental trajectories from day 0 to day 60 and to uncover or identify the minor endothelial population from day 60 cells (Supplementary Fig. 14o–t).

Finally, using three additional published temporal single cell expression datasets^6,9,10^, we demonstrated the capability of TCM to discover various subpopulations of cells from the late developmental stages, which were visually indistinguishable on the latent space produced by the other six algorithms (Supplementary Figs. 15–17).

## Discussion

We provide evidence that TCM overcomes the problems regarding the balance between the capability of preserving the global structure of gene expression and the sensitivity of discovering subpopulations of cells. Compared with other algorithms, the average percent of variance explained by non-temporal factors on 11 examined temporal expression datasets increases to 78.6% by using TCM, suggesting a significant reduction of the crowding problem^10,15^ of cells from the same time points (Fig. 1a). Downstream analysis such as trajectory inference^8,11^, cell clustering^13,23^ and differential expression analysis^14^ could be readily performed on the latent space produced by TCM. Furthermore, TCM provides a function for inferring developmental trajectories (Supplementary Note 8). We also recognize the limitations of this novel algorithm. TCM requires the scRNA-seq datasets with complete time index and the time index needs to be correlated with the underlying dynamic expression pattern. Otherwise, we recommend the use of the pseudotime index in conjunction with TCM or other generic dimension reduction tools (Supplementary Note 6). In addition, TCM does not provide immediate biological interpretation of cell clusters on the latent space, and further pathway analysis will need to be conducted to elucidate the biological meaning of each trajectory. In the future, the flexibility of the TCM framework will allow the extension of TCM to incorporate additional information such as spatial expression patterns and other–omics data and to provide accurate and comprehensive visual inspection of the biological progression and subpopulations of cells for the single cell studies. In summary, TCM is a novel tool to visualize developmental trajectories and discover hidden cell populations from time series single cell expression data. We provide the R implementation of TCM as a Supplementary Software Program.

## Methods

### The topographic cell map (TCM)

The topographic cell map (TCM) is a flexible probabilistic graphical model for modeling the temporal single cell RNA-seq (scRNA-seq) or single cell PCR (scPCR) data (Supplementary Fig. 1a).

Let **X**^(*t*)^ be a *N*×*M*^(*t*)^ observed read count matrix for *N* genes and *M*^(*t*)^ cells from time point *t*, in a temporal scRNA-seq expression dataset with total *M*_0_ cells and *T* time points, where *t* = 1,…,*T* and $M_0 = \mathop {\sum }\limits_{t = 1}^T M^{\left( t \right)}$, and $x_{n,m^{\left( t \right)}}$ be the read count of gene *n* in cell *m*^(*t*)^. We first introduced the modeling of scRNA-seq data from a single time point on a single 2D latent space, then extended the description to multiple time points on multiple latent spaces. To reduce the clutter of the notations, we first dropped the time index (*t*), and described how TCM models single cells from a single time point.

We modeled the observed read count *x*_*nm*_, as the sum of *K* auxiliary parameters, $s_{n,1,m}, \cdots ,s_{n,k,m}, \cdots ,s_{n,K,m}$, which represents the number of reads that can be explained by *K* components, respectively. We denoted each component as a *metagene*. The read counts that can be explained by the *k*-th metagene, *S*_*n*,*k*,*m*,_ further modeled as a Poisson distribution with the mean parameter *μ*_*n*,*k*,*m*_:

1 $$x_{nm} = \mathop {\sum }\limits_{k = 1}^K s_{n,k,m}$$

2 $$p\left({{\bf{S}}\vert{\bf{U}},{\bf{V}}}\right) = {\prod\limits_{n = 1}^N} {\prod\limits_{k = 1}^K} {\prod\limits_{m = 1}^M} {\mathrm{Pois}}\left( {s_{n,k,m}\vert \mu _{n,k,m}} \right)$$

This formulation takes advantage of the additive property of the Poisson distribution and was often used for modeling non-negative count data to simplify the following inference^24,25^.

The mean parameter *μ*_*n*,*k*,*m*_ for the Poisson distribution for each metagene was further modeled as the product of two parts:

3 $$\mu _{n,k,m} = u_{nk}{\mathrm{exp}}\left( {a_m + v_{km}} \right)$$

The cell independent metagene basis, *u*_*nk*_, models the non-negative expression levels of gene *n* in the *k*-th metagene, with a Gamma distribution prior with pre-specified shape parameter *c*_0_ and rate parameter *d*_0_, that is,

4 $$p\left( {\mathbf{U}} \right) = \mathop {\prod }\limits_{n = 1}^N \mathop {\prod }\limits_{k = 1}^K {\mathrm{Gamma}}\left( {u_{nk}|c_0,d_0} \right)$$

The metagene coefficient, *v*_*km*_, is a real variable, indicating the contribution of the *k*-th metagene for cell *m*. To account of the individual cell effect, we introduced a scaling parameter *a*_*m*_ for each cell *m*, which is positively correlated with the library size of cell *m* and allows *v*_*km*_ to only model the random effects of cell *m* of the *k*-th metagene.

### Modeling the cell-to-cell relationships

To capture the cell-to-cell relationships, TCM assumes cells reside on a low dimensional latent space, consisting of *H* units (prototypes), similar to the prototypes in self-organizing map (SOM)^26^ and generative topographic mapping (GTM)^18^. The prototypes form a pre-specified topographic structure, for example, a regular grid, as used in SOM and GTM modeling. In TCM, a *R* by *S* radial grid design is used to facilitate the convolution of prototypes between neighboring time points, where *R* represents the number of layers of prototypes and *S* represents the number of prototypes per layer (Fig. 1b, Supplementary Fig. 1a). The total number of prototypes on the latent space is therefore defined as *H* = *R*×*S*.

Each prototype on the latent space is represented by a unique *K*-dimensional metagene coefficient $\phi _h(h = 1, \cdots ,H)$ and has respective coordinates ***y***_h_ on the 2D space. We modeled each cell as a Gaussian mixture of all prototypes on the latent space, that is,

5 $$p\left( {\boldsymbol{\pi }} \right) = {\mathrm{Dir}}\left( {{\boldsymbol{\pi }}|\alpha _0} \right)$$

6 $$p\left( {{\mathbf{Z}}|{\boldsymbol{\pi }}} \right) = \mathop {\prod }\limits_{m = 1}^M \mathop {\prod }\limits_{h = 1}^H \left( {\pi _h} \right)^{z_{mh}}$$

7 $$p\left( {{\mathbf{V}}|{\mathbf{\Theta }},{\mathbf{Z}}} \right) = \mathop {\prod }\limits_{m = 1}^M \mathop {\prod }\limits_{h = 1}^H {\cal N}\left( {{\boldsymbol{v}}_m|\phi _h,\left( {\beta {\mathbf{I}}} \right)^{ - 1}} \right)^{z_{mh}}$$

where ***v***_*m*_ is the *K*-dimensional metagene coefficient for cell *m*, *β* is the inverse variance and *α*_0_ is a pre-specified parameter for the Dirichlet prior.

### Prototype coordinates on the 2D latent space

The 2D coordinate of the *h*-th prototype on the 2D latent space is represented as:

8 $${\boldsymbol{y}}_h = \left( {l_rcos\omega _s,l_rsin\omega _s} \right)$$

assuming that the prototype locates at the *r*-th layer with the polar angle *ω*_*s*_, where $r \in \left[ {1, \cdots ,R} \right]$, $s \in \left[ {1, \cdots ,S} \right]$, $\omega _s = \frac{s}{S}2\pi$, and $l_r = \frac{r}{R}$.

It should be noted that since TCM is a prototype-based dimension reduction method, multiple cells could possibly be mapped onto one prototype, and these cells would be visually indistinguishable. In order to separate the cells mapped onto one prototype, their 2D coordinates were added random Gaussian noise.

### Gaussian process (GP) prior for free prototypes

To ensure the neighboring free prototypes have similar metagene coefficients so that the transition from every prototype toward its neighboring prototypes is smooth, a Gaussian process (GP) prior was used to regularize the free prototypes, similar with the formulation in the GTM^18^. Specifically, for each metagene *k*, let ${\boldsymbol{\theta }}_{k,1:H}$ be a vector of length *H* consisting of the *k*-th metagene of ***θ***_1_ though ***θ***_*H*_. Consider a Gaussian prior distribution on the center location given by

9 $$p\left( {\mathbf{\Theta }} \right) = \mathop {\prod }\limits_{k = 1}^K {\cal N}\left( {{\boldsymbol{\theta }}_{k,1:H}|0,{\mathbf{B}}} \right)$$

where **B** is a positive definite matrix. The theory of Gaussian process regression allows **B** to be quite general. The covariance between ***θ***_*ki*_ and ***θ***_*kj*_ can be taken to depend on the 2D coordinates of their respective prototype ***y***_*i*_ and ***y***_*j*_, so that *B*_*ij*_ = *f*(***y***_*i*_,***y***_*j*_), where $f\left( \cdot \right)$ is a covariance function. In this study, we used a simple radial basis function (RBF) kernel, that is:

10 $$B_{ij} = {\mathrm{exp}}\left( { - \frac{{\left\| {{\boldsymbol{y}}_i - {\boldsymbol{y}}_j} \right\|_2^2}}{{2s_0}}} \right)$$

where *s*_0_ is a pre-specified scaling parameter for controlling the tightness of underlying 2D latent space (i.e., how similar the neighboring prototypes should be).

It should be noted that the prototype coordinates (***y***) on the 2D latent space were only used to determine the covariance structure **B** of the prototypes, along with a suitable covariance function. The 2D latent space, however, does not assume that the cell evolution process is linear on a 2D space, and can also be used to describe non-linear process. Moreover, running TCM on a scRNA-seq dataset without time index can be viewed as a process of clustering single cells on the 2D latent space (Supplementary Note 5).

### Modeling temporal scRNA-seq using multiple latent spaces

TCM assigns cells from one time point to a corresponding time point specific latent space (e.g., cells from the *t*-th time point are mapped onto the *t*-th latent space). In the meanwhile, TCM also constrains the neighboring latent spaces so that the similar cells from the different time points should have a similar polar angle *ω*. This constraint is achieved by using the *convolving prototypes*. The convolving prototypes are a subset of prototypes on the latent space and convolve the neighboring latent spaces to produce a single latent space representing cells from all time points (Supplementary Fig. 1b).

Specifically, the convolving prototypes serve to associate the latent spaces from the previous time points. The convolving prototypes are defined as (*R*-*ρ*) inner layers of prototypes on the *t*-th latent space, thus the total number of convolving prototypes on the *t*-th latent space is $H_{\mathrm{{conv}}} = \left( {R - \rho } \right) \times S$.

On the other hand, the *free prototypes* are defined as *ρ* outer layers of non-convolving prototypes on the *t*-th latent space where $1 < \rho \le R$, thus the total number of free prototypes on the *t*-th latent space is *H*_*free*_ = *ρ*×*S*.

Thus, we iteratively define:

11 $$\phi _h^{\left( t \right)} = \left\{ {\begin{array}{*{20}{c}} {{\boldsymbol{\theta }}_h^{\left( t \right)},h\,{\mathrm{is}}\,{\mathrm{a}}\,{\mathrm{free}}\,{\mathrm{prototype}}} \\ {\mathop {\sum }\limits_{i = 1}^H w_{\mathrm{{hi}}}^{\left( t \right)}\phi _i^{\left( {t - 1} \right)},h\,{\mathrm{is}}\,{\mathrm{a}}\,{\mathrm{convolving}}\,{\mathrm{prototype}}} \end{array}} \right.$$

where ${\boldsymbol{\theta }}_h^{\left( t \right)}$ represents the metagene coefficients of the free prototype *h* on the *t*-th latent space. The metagene coefficients of the convolving prototypes on the *t*-th latent space are deterministically computed as the convex combination of metagene coefficients of all prototypes on the (*t*-1)-th latent space:

12 $$w_{hi}^{\left( t \right)} = \frac{{{\mathrm{exp}}\left( { - \left\| {\frac{R}{{R - \rho }}{\boldsymbol{y}}_h^{\left( t \right)} - {\boldsymbol{y}}_i^{\left( {t - 1} \right)}} \right\|_2^2} \right)}}{{\mathop {\sum }\nolimits_{j = 1}^H {\mathrm{exp}}\left( { - \left\| {\frac{R}{{R - \rho }}{\boldsymbol{y}}_h^{\left( t \right)} - {\boldsymbol{y}}_j^{\left( {t - 1} \right)}} \right\|_2^2} \right)}}$$

where ${\boldsymbol{y}}_h^{\left( t \right)}$ is the coordinate of prototype *h* on the *t*-th latent space. We assume that all the prototypes are free prototypes for the first time point (*t*=1). Therefore, any convolving prototypes on the latent spaces can be represented as a linear function of all free prototypes.

After the fitting of TCM, every cell’s *m*^(*t*)^ from the *t*-th time point is assigned to the most similar prototype on the *t*-th latent space $\phi _h^{\left( t \right)}$ where $h = {\mathrm{argmax}}\,p\left( {z_{m^{\left( t \right)},h}^{\left( t \right)}} \right)$ and the 2D coordinates for prototype *h* is ${\boldsymbol{y}}_h^{\left( t \right)} = \left( {l_r^{\left( t \right)}cos\omega _s^{\left( t \right)},l_r^{\left( t \right)}sin\omega _s^{\left( t \right)}} \right)$. We define a single latent space to visualize cells from all time points together. The coordinates on such single latent space for ${\boldsymbol{y}}_h^{\left( t \right)}$ is represented as ${\boldsymbol{y}}_h^\prime = \left( {\ln \lambda _r^{\left( t \right)}cos\omega _s^{\left( t \right)},\ln \lambda _r^{\left( t \right)}sin\omega _s^{\left( t \right)}} \right)$, where $\ln \lambda _r^{\left( t \right)}$ is the new radius for prototype *h* on the single latent space (the polar angle remains the same). We recursively define $\lambda _r^{\left( t \right)} = \frac{R}{{R - \rho }}{\mathrm{max}}\left( {\lambda ^{\left( {t - 1} \right)}} \right)l_r^{\left( t \right)}$and $\lambda _r^{\left( 1 \right)} = l_r^{\left( 1 \right)}$ for the first time point.

### Human iPSC differentiation

To induce the hiPSC (PLZ) toward the cardiovascular fate, we added Activin A and small molecule CHIR-99021, an activator of the Wnt signaling pathway (GSK3 inhibitor) on differentiation day 0, followed by adding FGF2 and BMP4 on day 1 to induce the mesodermal specification^27^. On day 3, we added IWP4 (Wnt inhibitor) to block the accumulation of *β*-catenin, increasing CM differentiation efficiency^28^. A base medium containing RPMI 1640 (Hyclone) and B27 supplement without insulin (RPMI-) was used from day 0 until day 4 of the differentiation. From day 5 until collection, cells were cultured in RPMI 1640 and B27 supplement with insulin (RPMI+) until collection. The 324 single cells from differentiation day 0 (D0), 6, 10, 30, and 60 were captured by a Fluidigm 10-17μm integrated fluidics circuit (IFC), followed by viability screening, lysis and library amplification on a C1 Single-Cell Auto Prep System. All cells were collected by dissociation using TrypLE Express (Life Technologies) with aliquots taken for single cell capture, flow cytometry, immunohistochemistry, and Total RNA.

### Flow cytometry analysis for cTNT

Cell samples were fixed using 1% paraformaldehyde (PFA) in PBS at 37 °C for 10 min in a dark water bath and permeabilized in 90% methanol on ice for 30 min. A FACS buffer (PBS without Ca/Mg^2+^, 0.5% BSA, 0.1% NaN_3_, and 0.1% Triton X-100) was used to wash the cells and after centrifugation to dilute each sample in with primary antibody (cTNT, Thermo Scientific, clone 13-11) in 200 μL. Samples were incubated at 4 °C overnight in the dark. Cells were then washed in 1 mL of FACS buffer after centrifugation and the secondary antibody (Donkey α-mouse IgG with APC, Jackson ImmunoResearch), was applied diluted in FACS buffer with a final volume of 200 μL at a 1:500 dilution. Samples were incubated at room temperature for 30 min and then washed with FACS buffer. After centrifugation, the cells were resuspended in FACS buffer with propidium iodide (Life Technologies) diluted 1:2,000 for analysis. A FACSAria (BD) was used to collect data and analyzed using FlowJo (v10.0.8r1).

### Immunostaining

Cell samples were plated on glass coverslips coated with Matrigel on the day of capture and cultured for 24 h at 37 °C in RPMI + with Y-27632 (10 uM, ATCC). After 24 h, coverslips were washed with PBS and fixed using 4% PFA in PBS for 10 min at room temperature. Coverslips were then washed 3 times with PBS before staining.

### qRT-PCR

Cell samples were collected in a 1.5 mL Eppendorf tube and centrifuged at 200×*g* in a refrigerated microcentrifuge (Eppendorf). The supernatant was aspirated and 300 μL of lysis buffer (Invitrogen) with 1% 2-Mercaptoethanol (Sigma) was added to the tube.

### Single cell RNA-seq of differentiated human iPSCs

All libraries were sequenced using 75-bp paired end sequencing on MiSeq (Illuminia). The cells with less than 100 K paired reads were removed, resulting in 315 cells for analysis. The raw read counts for each gene were obtained with TopHat (v2.0.13) and HTSeq (v0.6.0) with default parameters^29,30^. The median mapping rate was 89.2%. The raw read counts were normalized by the size factor^31^.

### Code availability

TCM was optimized by a standard variational inference algorithm (Supplementary Note 4) or a fast stochastic variational inference (SVI) based method (Supplementary Note 7). The TCM R package is freely available under the MIT license at https://github.com/gongx030/tcm and as Supplementary Software.

### Data availability

The single cell RNA-seq data that support the findings of this study have been deposited in NCBI Sequence Read Archive (SRA) database with the project accession number PRJNA438778. The TCM software was freely available at https://github.com/gongx030/tcm. All other relevant data are available from the authors.

## Electronic supplementary material

Supplementary Information
