# Supplementary material for: TCM visualizes trajectories and cell populations from single cell data
Source: Nat Commun. 2018 Jul 16;9:2749. doi: 10.1038/s41467-018-05112-9 (PMC6048168; doi:10.1038/s41467-018-05112-9)
Supplement: Supplementary file 1 — Supplementary Information [file 41467_2018_5112_MOESM1_ESM.pdf]

Supplementary Notes for

TCM visualizes trajectories and cell populations from single cell data

Gong et al.

Supplementary Figure 1

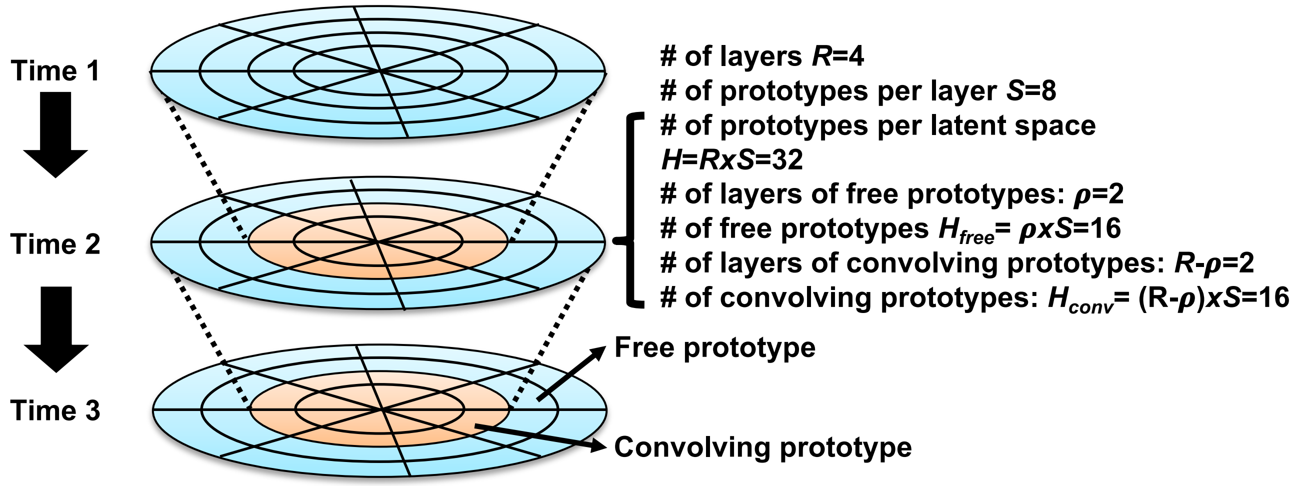

Supplementary Figure 1. Schematic representation of the Topographic Cell Map (TCM) model. In TCM, the cells from each time point are simultaneously mapped to multiple time point specific latent spaces, preventing the cells from the same time points from crowding together due to the high temporal variance typically present in the time series expression datasets. Specifically, TCM assumes that cells from time point t reside in separate low dimensional latent space, consisting of H units (prototypes). The prototypes form a specified $R \times S$ radial grid, where R represents the number of layers of prototypes and S represents the number of prototypes per layer on the t-th latent space. The total number of prototypes on the t-th latent space is therefore defined as $H = R \times S$. The free prototypes are defined as ρ outer layers of prototypes on the t-th latent space where $1 < \rho \leq R$, thus the total number of free prototypes on the t-th latent space is $H_{free} = \rho \times S$. The convolving prototypes serve to associate the latent spaces from the previous time points. The convolving prototypes are defined as $(R - \rho)$ inner layers of prototypes on the t-th latent space, thus the total number of convolving prototypes on the t-th latent space is $H_{conv} = (R - \rho) \times S$.

Supplementary Figure 2

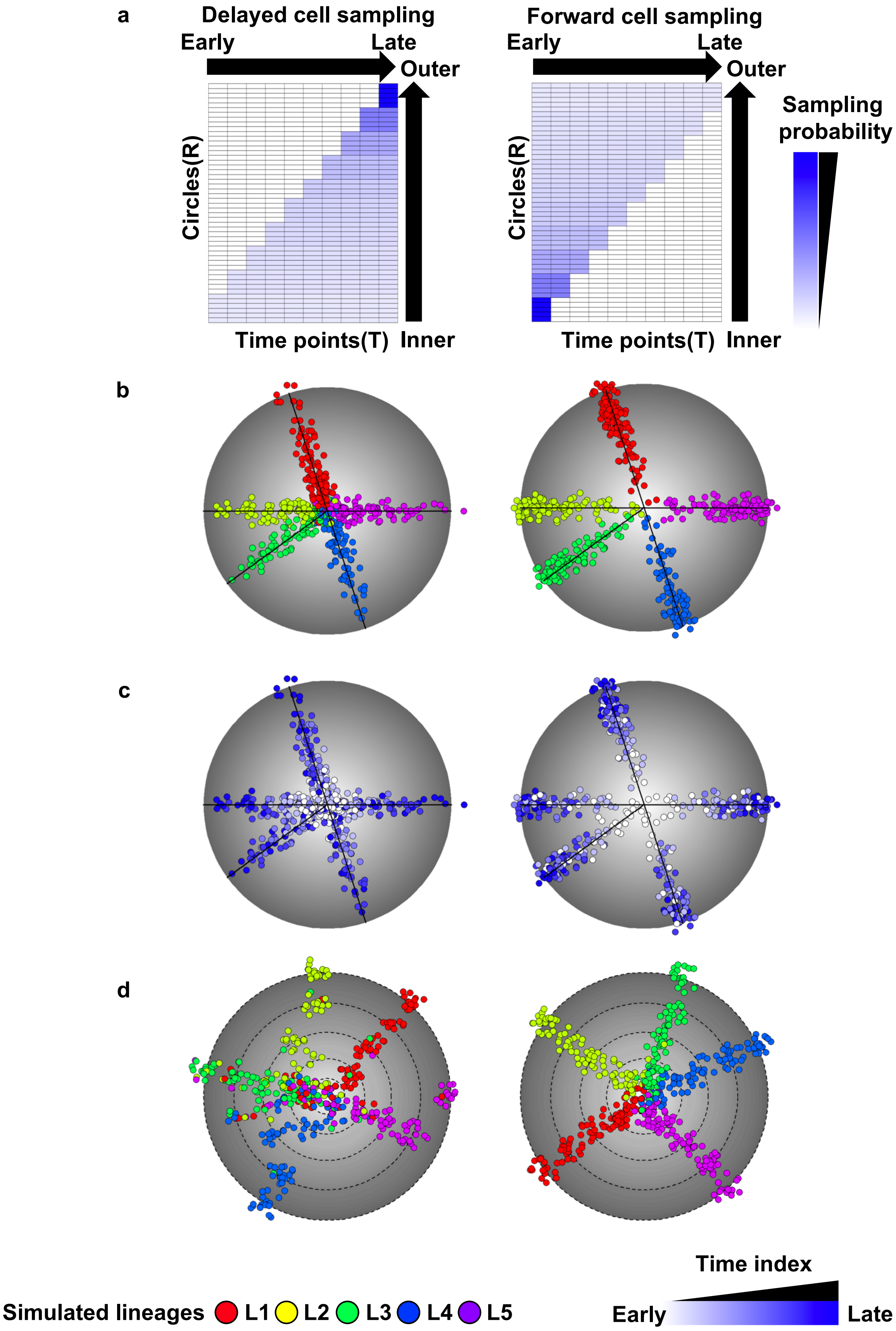

Supplementary Figure 2

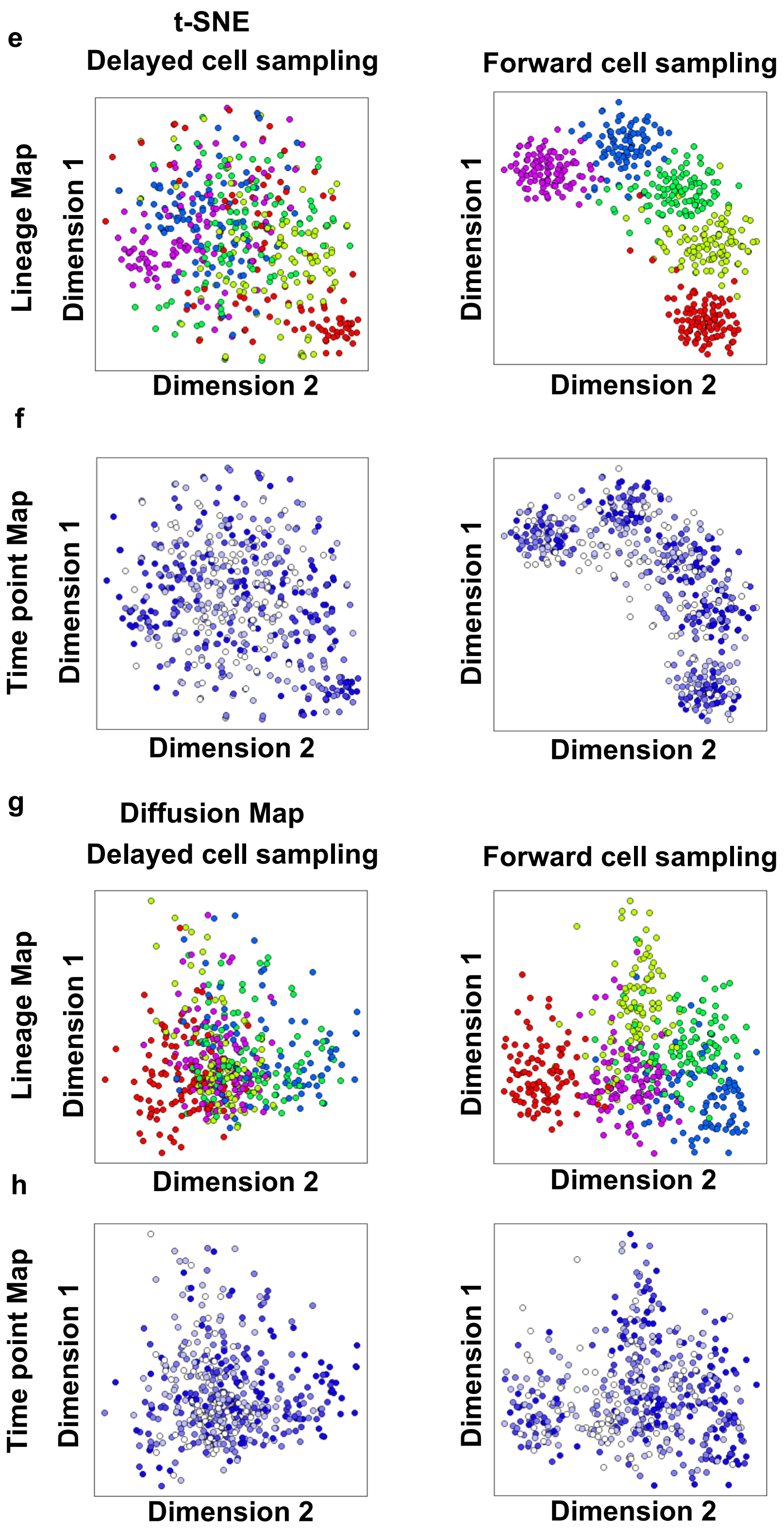

Supplementary Figure 2

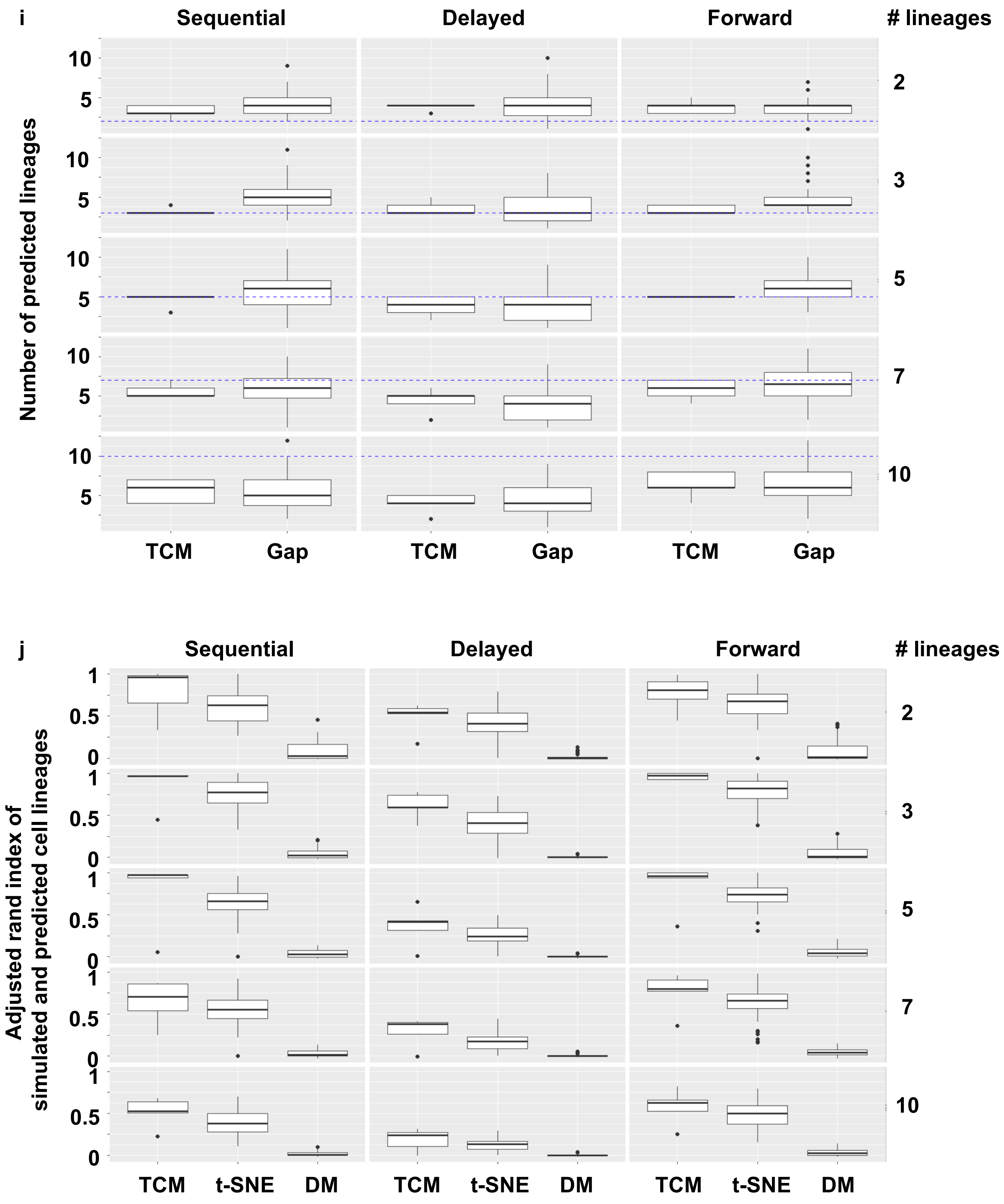

Supplementary Figure 2. TCM has improved performance for the detection of subpopulations of cells with three two asynchronized differentiation models: delayed and forward cell sampling. (a) The heatmap shows the sampling probabilities for three types of differentiation models. In the delayed cell sampling, the more primitive (progenitor) cells could be sampled at a later time point, while in the forward cell sampling, the more differentiated cells could be sampled at an earlier time point. **(b-c)** The simulated temporal scRNA-seq datasets with five lineages under three differentiation models ($N = 2{,}000$ genes and $M = 500$ cells, with an exponential decay model for the dropout noise), with the color indicating **(b)** the cell lineages or **(c)** time index. **(d)** TCM was able to successfully reveal the lineage trajectories for two asynchronized differentiation models. **(e-h)** The visualization of simulated temporal scRNA-seq datasets under two differentiation models by **(e-f)** t-SNE and **(g-h)** diffusion map. **(i)** TCM has better performance for the determination of the number of cell clusters than gap-statistics on simulated temporal scRNA-seq data with a different number of lineages ($\boldsymbol{L} = 2{,}3{,}5{,}7{,}10$) under three different differentiation models ($N = 2{,}000$ genes, $M = 500$ cells and $T = 5$ time points, with an exponential decay model for the dropout noise). For each parameter configuration, the experiments were repeated 20 times. **(j)** TCM has better performance for the separation of the cells from different lineages compared to t-SNE and diffusion map followed by *k*-means, on simulated temporal scRNA-seq data with different number of lineages under three different differentiation models. In boxplot, the upper whisker is located at the smaller of the maximum input value and Q_3+1.5*IQR, and the lower whisker is located at the larger of the smallest input value and Q_1-1.5*IQR, where Q_1 and Q_3 are the first and third quantile of the input data, and IQR = Q_3 - Q_1, the box length.

Supplementary Figure 3

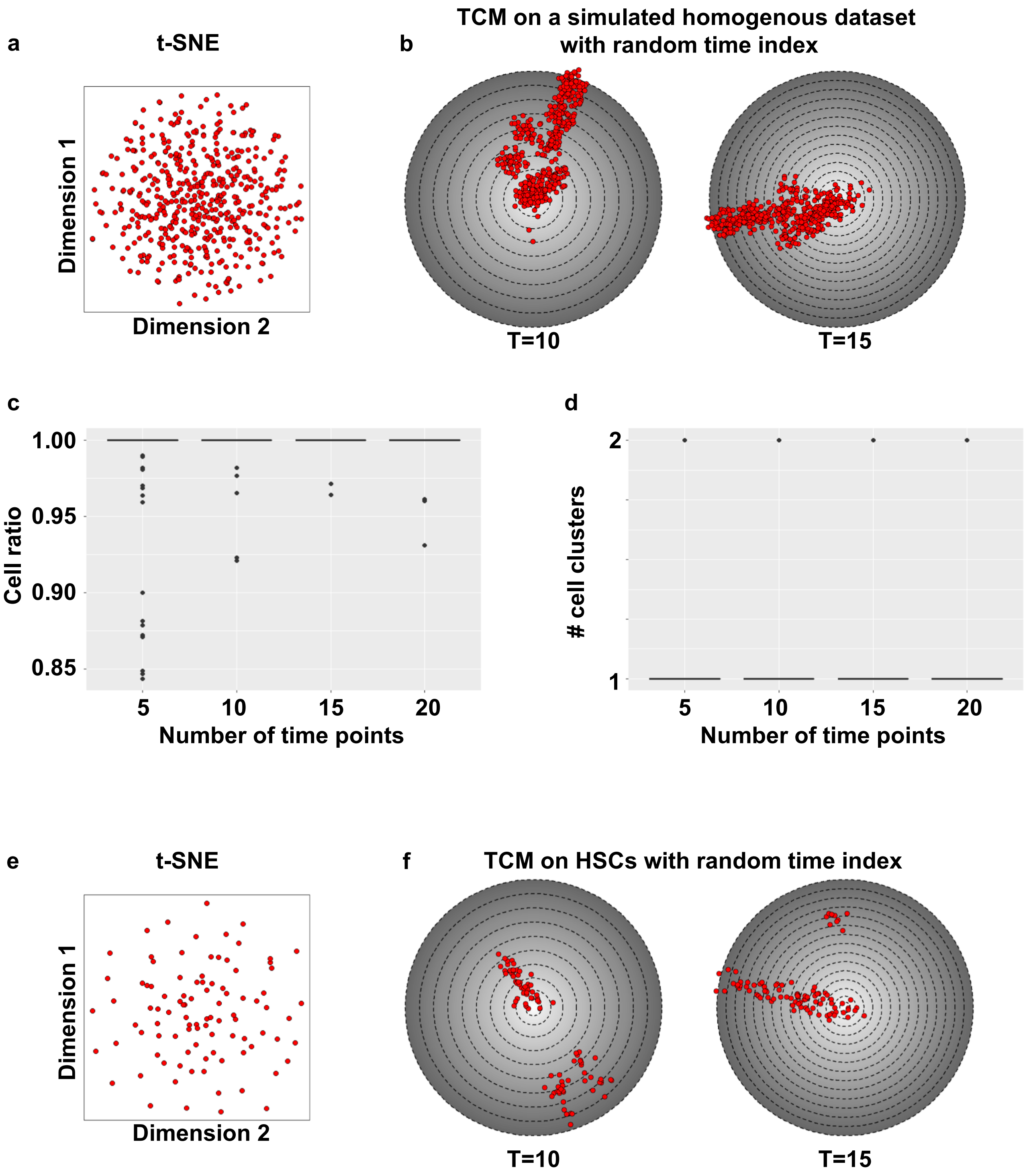

Supplementary Figure 3

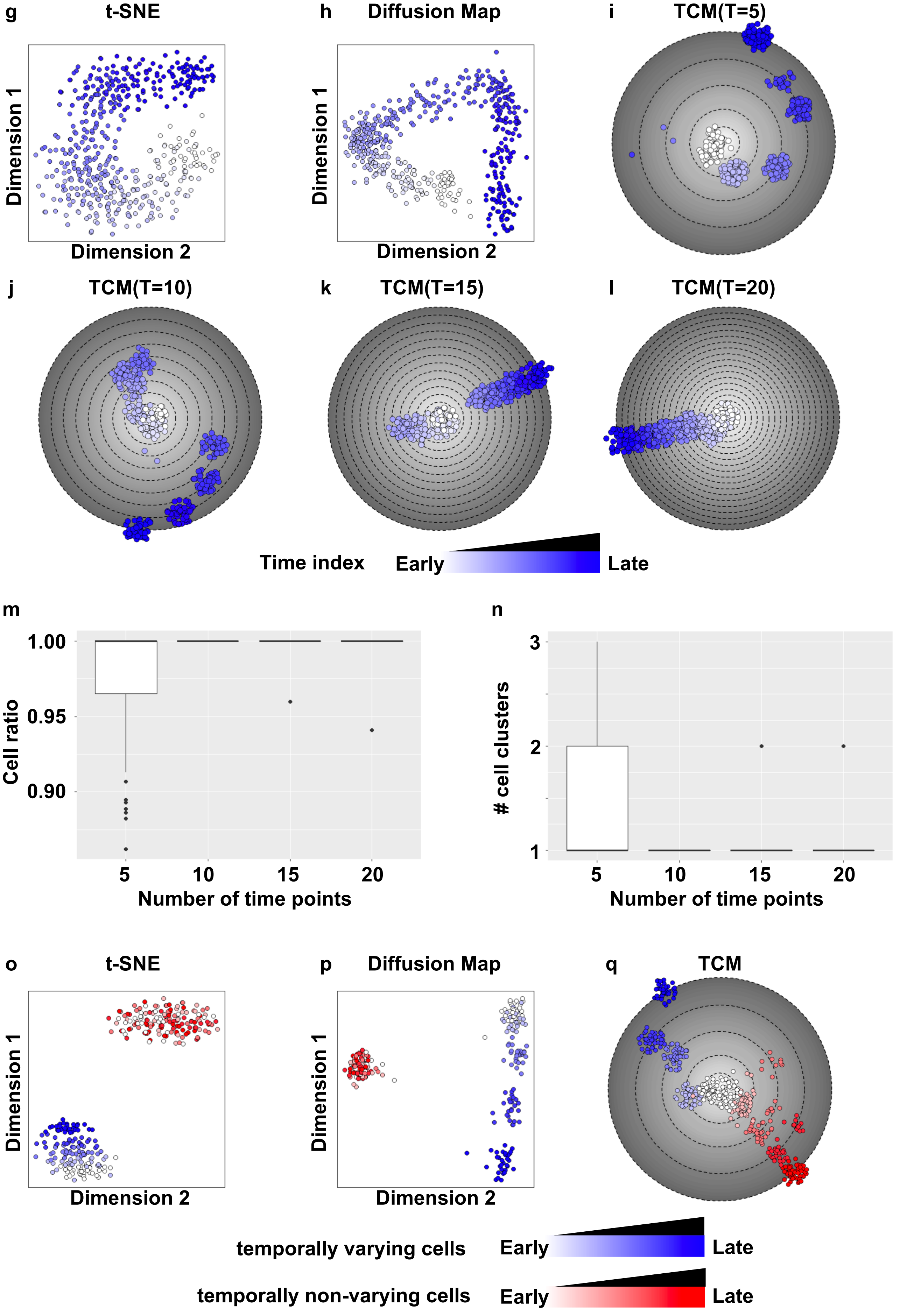

Supplementary Figure 3. TCM is unlikely to generate false positive lineages on homogenous scRNA-seq data and temporal scRNA-seq datasets from a single lineage. **(a)** The visualization of a simulated homogenous scRNA-seq dataset using the t-SNE algorithm ($N = 2{,}000$ genes and $M = 500$ cells). **(b)** TCM grouped the cells into a single trajectory from the central to the peripheral area. The homogenous scRNA-seq dataset was randomly split into 10 or 15 time points. **(c)** The proportions of cells that were assigned to the largest cluster and **(d)** the number of cell clusters that were generated using TCM on 20 simulated homogenous scRNA-seq datasets with arbitrary time index. The simulated homogenous scRNA-seq datasets were randomly split into $T = 5{,}10{,}15{,}20$ time points. **(e)** The visualization of a relatively homogenous hematopoietic stem cell (HSC) population using t-SNE. **(f)** TCM grouped the HSCs into a single trajectory from the central to the peripheral area. The HSC dataset was randomly split into 10 or 15 time points. **(g-h)** The visualization of simulated temporal scRNA-seq dataset of linear differentiation ($N = 2{,}000$ genes and $M = 500$ cells, with an exponential decay model for the dropout noise) by **(h)** t-SNE and **(h)** diffusion map. **(i-l)** The visualization of the simulated temporal scRNA-seq dataset of linear differentiation using t-SNE. The cells were sampled at $T = 5{,}10{,}15{,}20$ time points. The color indicates the time index associated with individual cells. **(m-n)** TCM is likely to produce a single trajectory from the simulated temporal scRNA-seq dataset of linear differentiation. The evaluation was performed at four different time points (5, 10, 15, 20) and by the metrics **(m)** the proportions of cells that were assigned to the largest cluster, and **(n)** the number of cell clusters that were generated, among the cells from the last time point. For each parameter configuration, the experiments were repeated 20 times. **(o)** TCM revealed two distinct major trajectories for simulated scRNA-seq data with temporally varying and non-varying cells. **(p-q)** Visualization of simulated scRNA-seq data by **(p)** t-SNE and **(q)** diffusion map. In boxplot, the upper whisker is located at the smaller of the maximum input value and Q_3+1.5*IQR, and the lower whisker is located at the larger of the smallest input

value and Q_1-1.5*IQR, where Q_1 and Q_3 are the first and third quantile of the input data, and IQR = Q_3 - Q_1, the box length.

Supplementary Figure 4

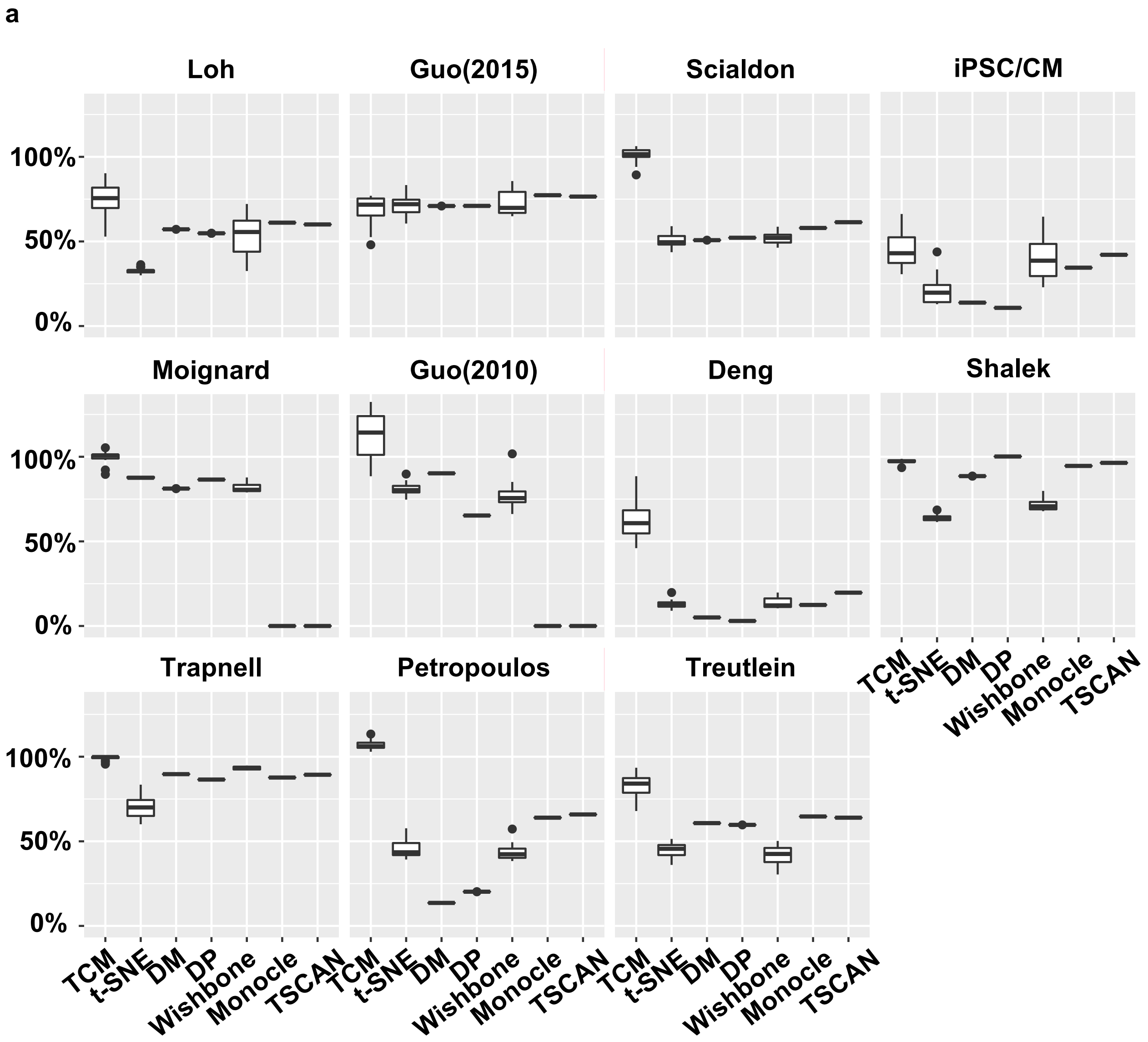

Supplementary Figure 4. TCM reduces the variance due to temporal factors on the latent space and preserves the global developmental trajectories for the visualization of temporal single cell expression data. (a) The boxplots indicate the percent of variance explained by non-temporal factors on the two dimensional latent space produced by TCM, t-SNE, diffusion map (DM), diffusion pseudotime (DP), Wishbone, Monocle and TSCAN on 11 examined single cell expression datasets. The lower percentage suggests the latent space is more dominated by the temporal variance. In boxplot, the upper whisker is located at the smaller of the maximum input value and Q_3+1.5*IQR, and the lower whisker is located at the larger of the smallest input value and Q_1-1.5*IQR, where Q_1 and Q_3 are the first and third quantile of the input data, and IQR = Q_3 - Q_1, the box length.

Supplementary Figure 5

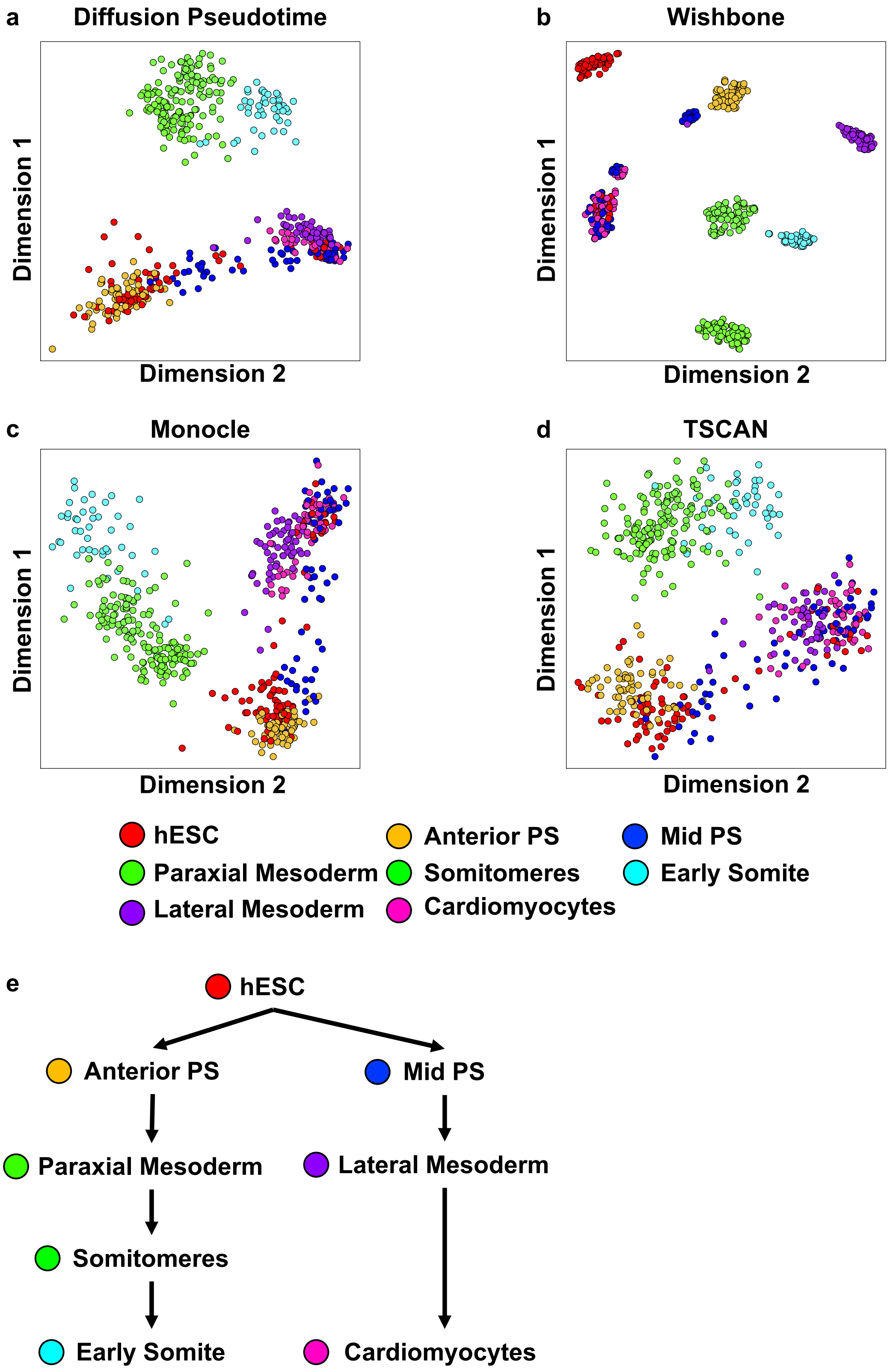

Supplementary Figure 5. Visualization of a scRNA-seq dataset of hESC derived mesodermal lineages by **(a)** Diffusion pseudotime, **(b)** Wishbone, **(c)** Monocle and **(d)** TSCAN. **(e)** A schematic of the differentiation process of hESC derived mesodermal lineages.

Supplementary Figure 6

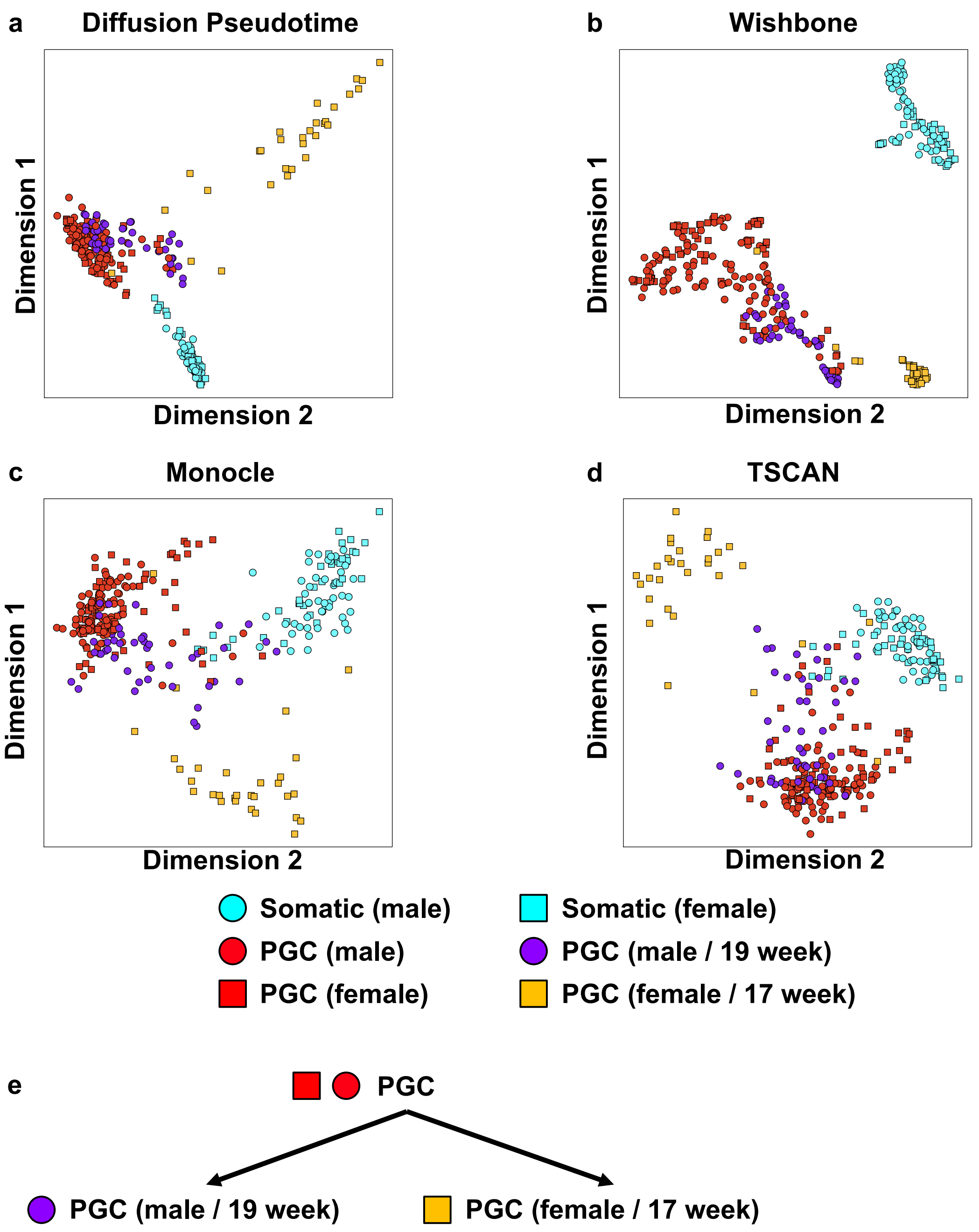

Supplementary Figure 6. Visualization of a scRNA-seq dataset of the development of human primordial germ cells (PGC) and neighboring somatic cells from weeks 4 to 19 post-gestation using **(a)** Diffusion pseudotime, **(b)** Wishbone, **(c)** Monocle and **(d)** TSCAN. **(e)** A schematic of the development of human primordial germ cells (PGC) and neighboring somatic cells from weeks 4 to 19 post-gestation.

Supplementary Figure 7

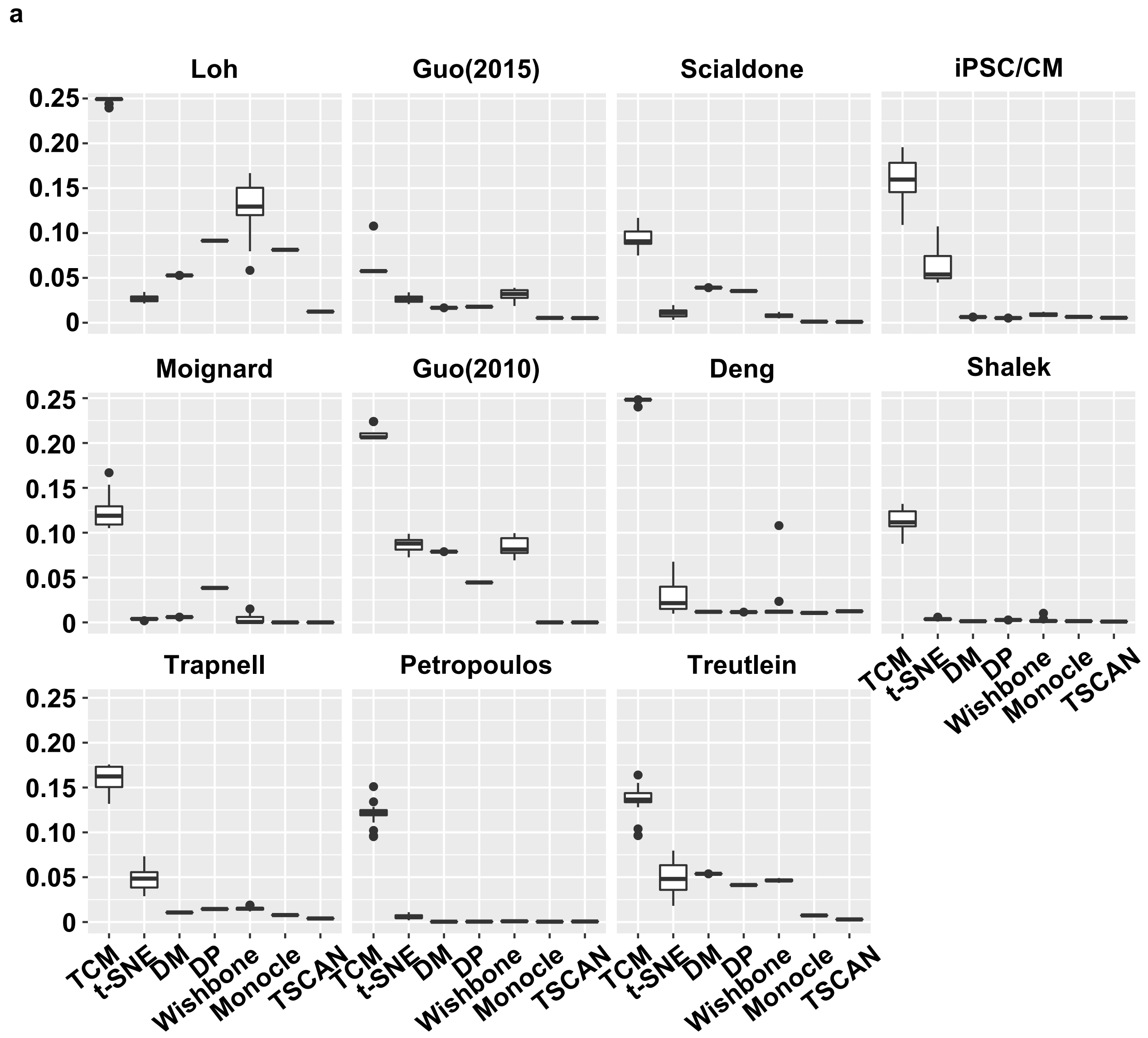

Supplementary Figure 7. TCM identifies subpopulations of cells from the last time point for the visualization of temporal single cell expression data. **(a)** The boxplots indicate the capability of separating subpopulations from the last time point on the two dimensional latent space produced by TCM, t-SNE, diffusion map (DM), diffusion pseudotime (DP), Wishbone, Monocle and TSCAN on 11 examined single cell expression datasets. The performance is quantitatively measured by Hartigan's Dip statistics using the cells' coordinates on the latent space. The high Dip score suggests the cells from the last time point are more separated on the latent space. In boxplot, the upper whisker is located at the smaller of the maximum input value and Q_3+1.5*IQR, and the lower whisker is located at the larger of the smallest input value and Q_1-1.5*IQR, where Q_1 and Q_3 are the first and third quantile of the input data, and IQR = Q_3 - Q_1, the box length.

Supplementary Figure 8

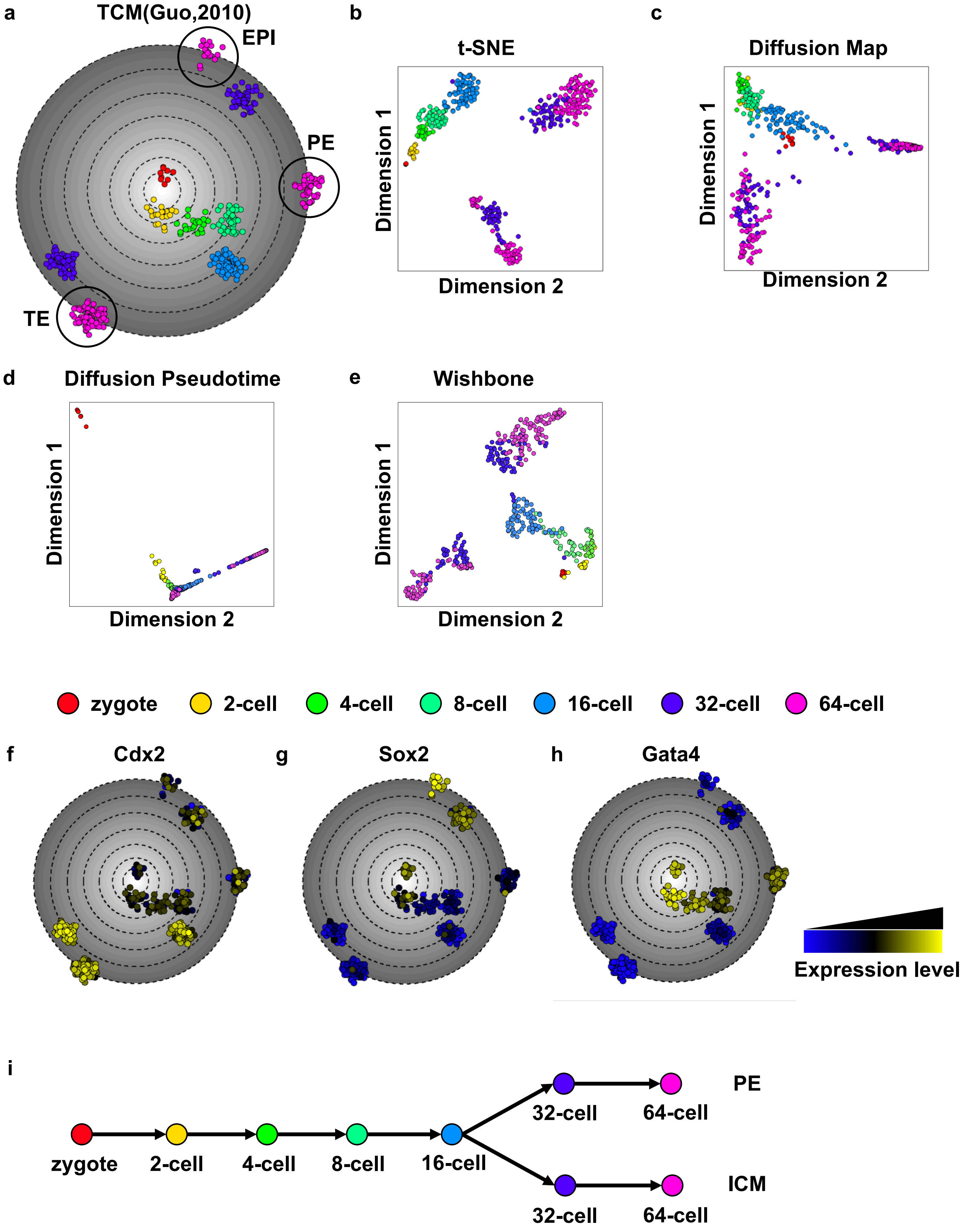

Supplementary Figure 8. TCM preserves the global developmental trajectories for the visualization of single cell PCR (scPCR) of early mouse embryonic development. **(a)** TCM is used to visualize the scPCR of mouse preimplantation embryonic development, where the expression pattern of 442 single cells were profiled from zygotes, 2-cell, 4-cell, 8-cell, 16-cell, 32-cell and 64-cell embryonic stages, respectively. TCM successfully identifies the bifurcation of the inner cell mass (ICM) and trophectoderm (TE) at the 32-cell stage, and the further bifurcation of the epiblast (EPI) and primitive ectoderm (PE) from the ICM cells at the 64-cell stage. In comparison, **(b)** t-SNE and **(e)** Wishbone recover the major trajectories and the two bifurcations, while **(c)** diffusion map and **(d)** diffusion pseudotime captures the bifurcation of ICM and TE at the 16-cell stage, but not the further bifurcation between the EPI and PE at the 32-cell stage. **(f-h)** The bifurcations of ICM/TE, and EPI/PE using TCM are supported by the expression pattern of **(f)** Cdx2 (TE marker), **(g)** Sox2 (EPI and ICM marker), and **(h)** Gata4 (PE marker). **(i)** A schematic of early mouse embryonic development.

Supplementary Figure 9

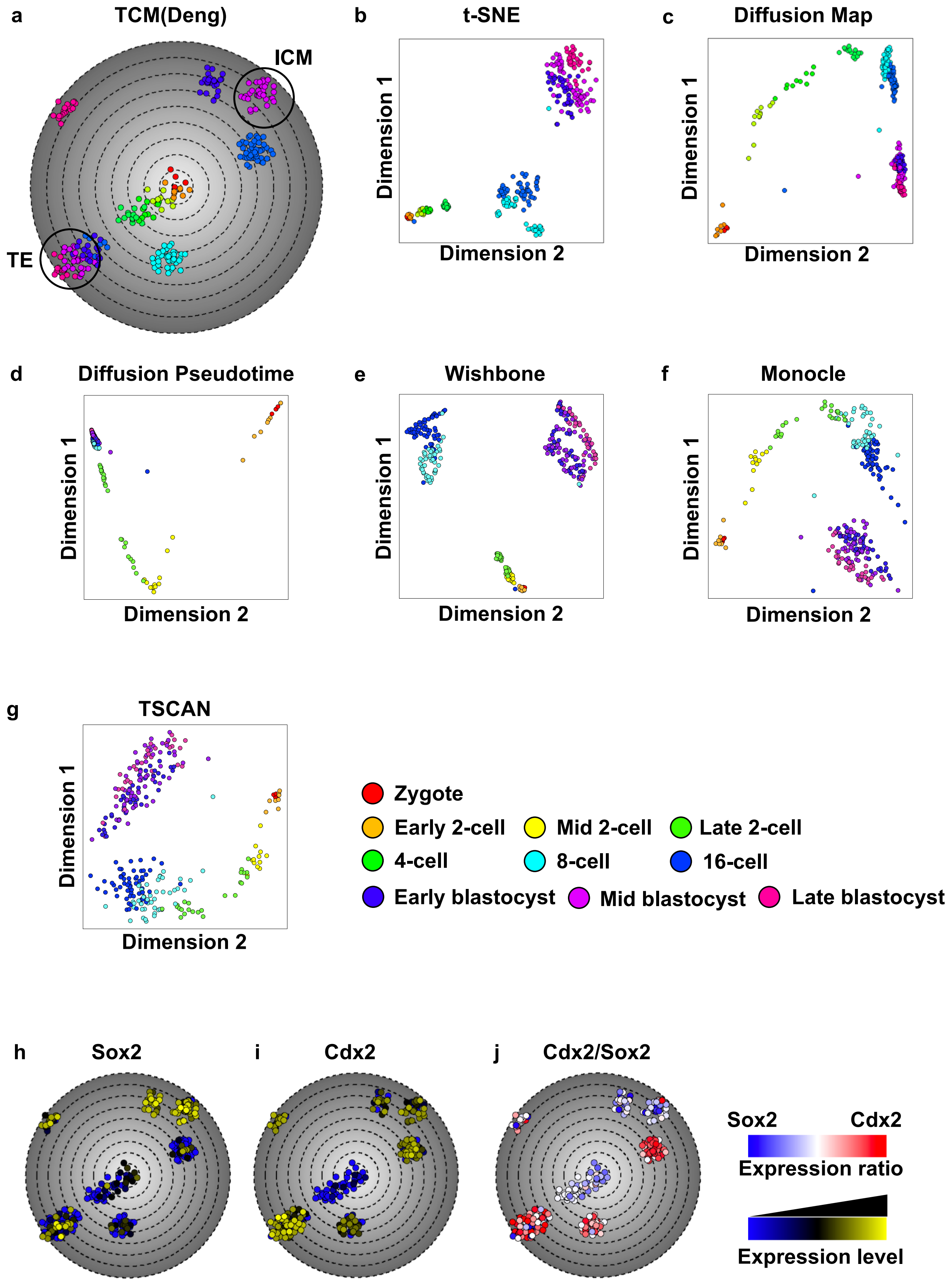

Supplementary Figure 9

k

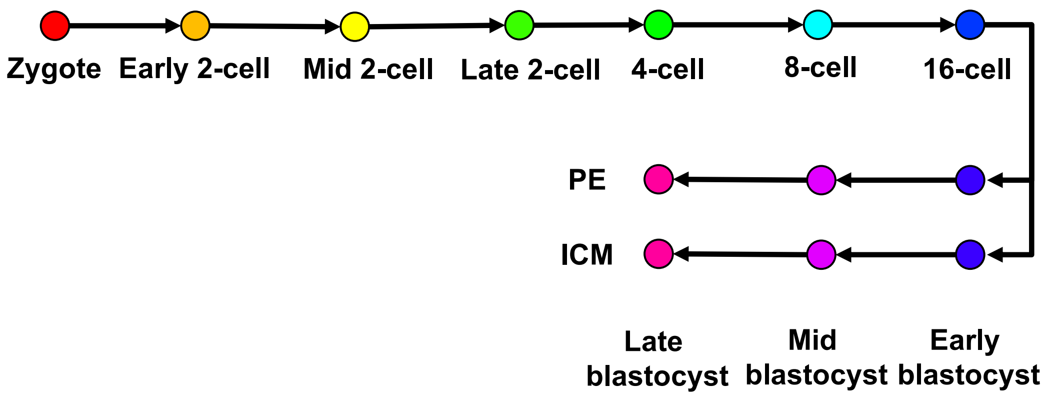

Supplementary Figure 9. TCM preserves the global developmental trajectories for the visualization of scRNA-seq of early mouse embryonic development. **(a)** TCM is used to visualize the scRNA-seq of mouse preimplantation embryonic development, where the expression pattern of 286 single cells were profiled from zygotes, early 2-cell, mid 2-cell, late 2-cell, 4-cell, 8-cell, 16-cell, early blastocyst, mid blastocyst and late blastocyst embryos, respectively. TCM successfully identifies the bifurcation of inner cell mass (ICM) and trophectoderm (TE) at the16-cell stage. In comparison, **(b-g)** all other methods fail to recover the bifurcation of ICM and TE. **(h-j)** The bifurcation of ICM/TE is supported by the expression pattern of **(h)** Sox2 (ICM marker), **(i)** Cdx2 (TE marker), and **(j)** the ratio of relative expression intensity between Cdx2 and Sox2. **(k)** A schematic of early mouse embryonic development.

Supplementary Figure 10

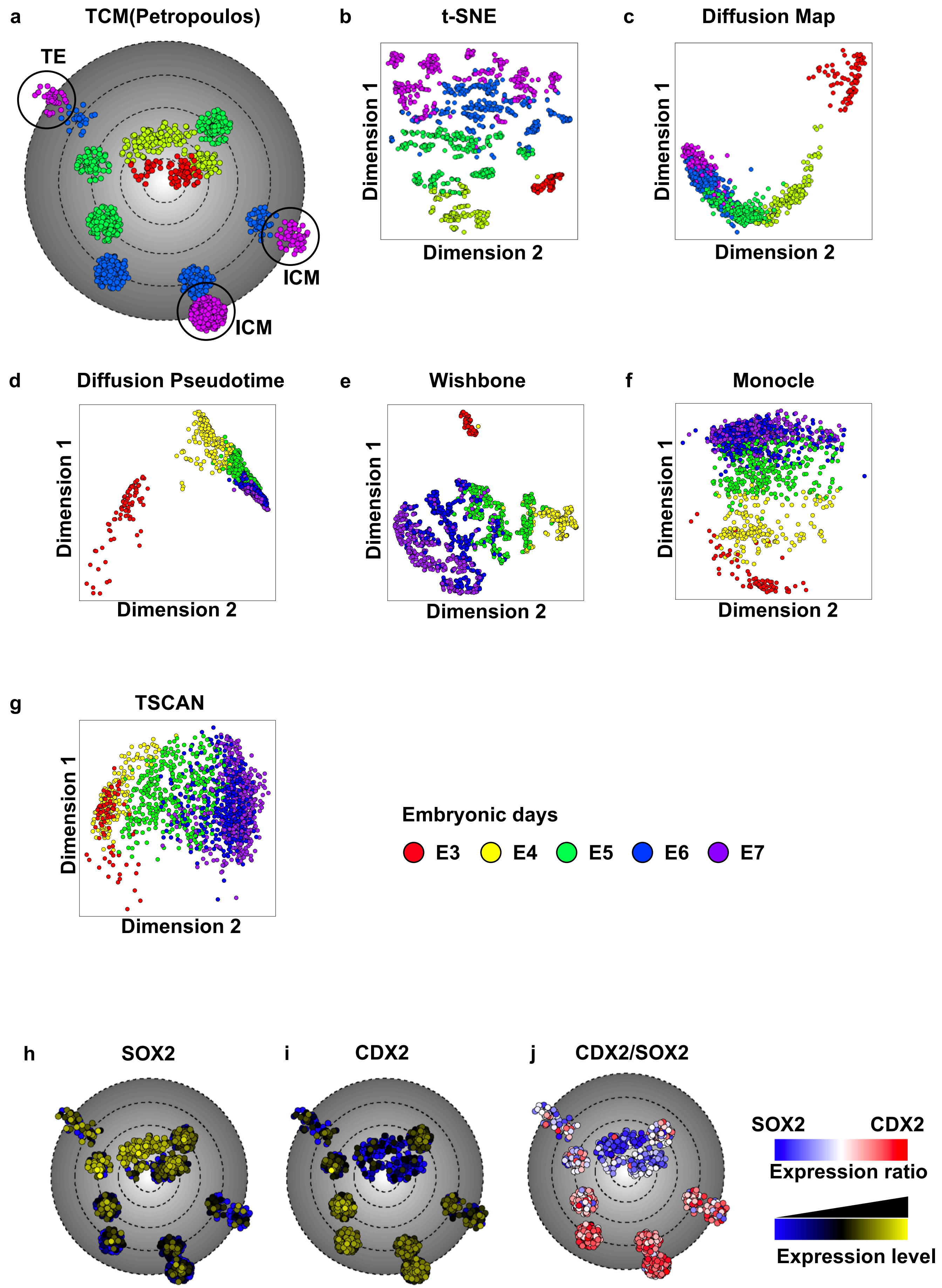

Supplementary Figure 10

k

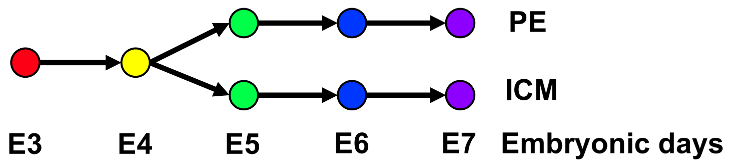

Supplementary Figure 10. TCM preserves the global developmental trajectories for the visualization of scRNA-seq of early human embryonic development. **(a)** TCM is used to visualize the scRNA-seq of human preimplantation embryonic development, where the expression pattern of 1,529 single cells were profiled from developmental day 3 (E3), day 4 (E4), day 5 (E5), day 6 (E6) and day 7 (E7) embryos, respectively. TCM successfully identifies the bifurcation of the inner cell mass (ICM) and trophectoderm (TE) at E5. In comparison, **(b-g)** all other methods fail to recover the bifurcation of ICM and TE. **(h-j)** The bifurcation of ICM/TE is supported by the expression pattern of **(h)** SOX2 (ICM marker), **(i)** CDX2 (TE marker), and **(j)** the ratio of relative expression intensity between CDX2 and SOX2. **(k)** A schematic of early human embryonic development.

Supplementary Figure 11

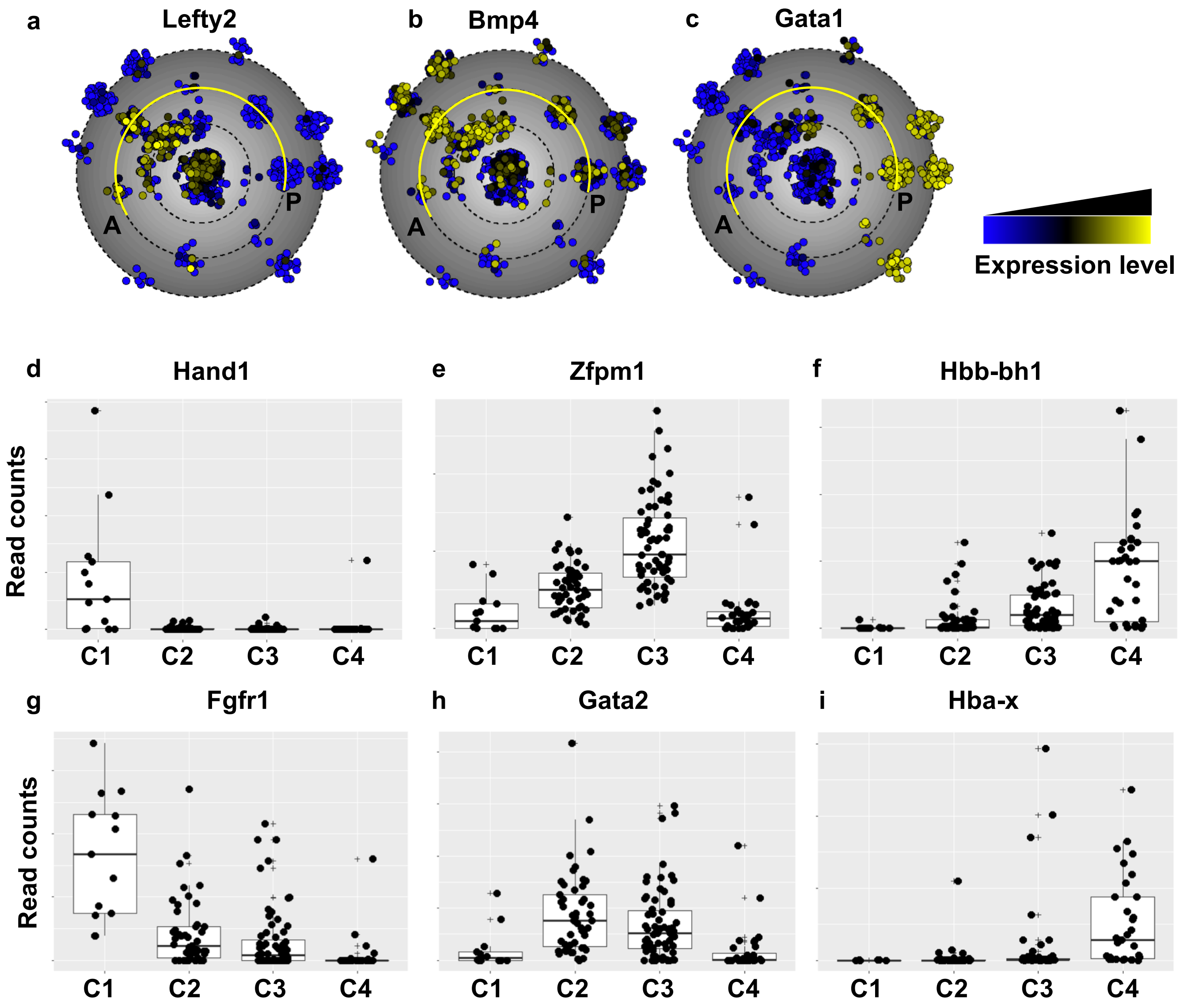

Supplementary Figure 11

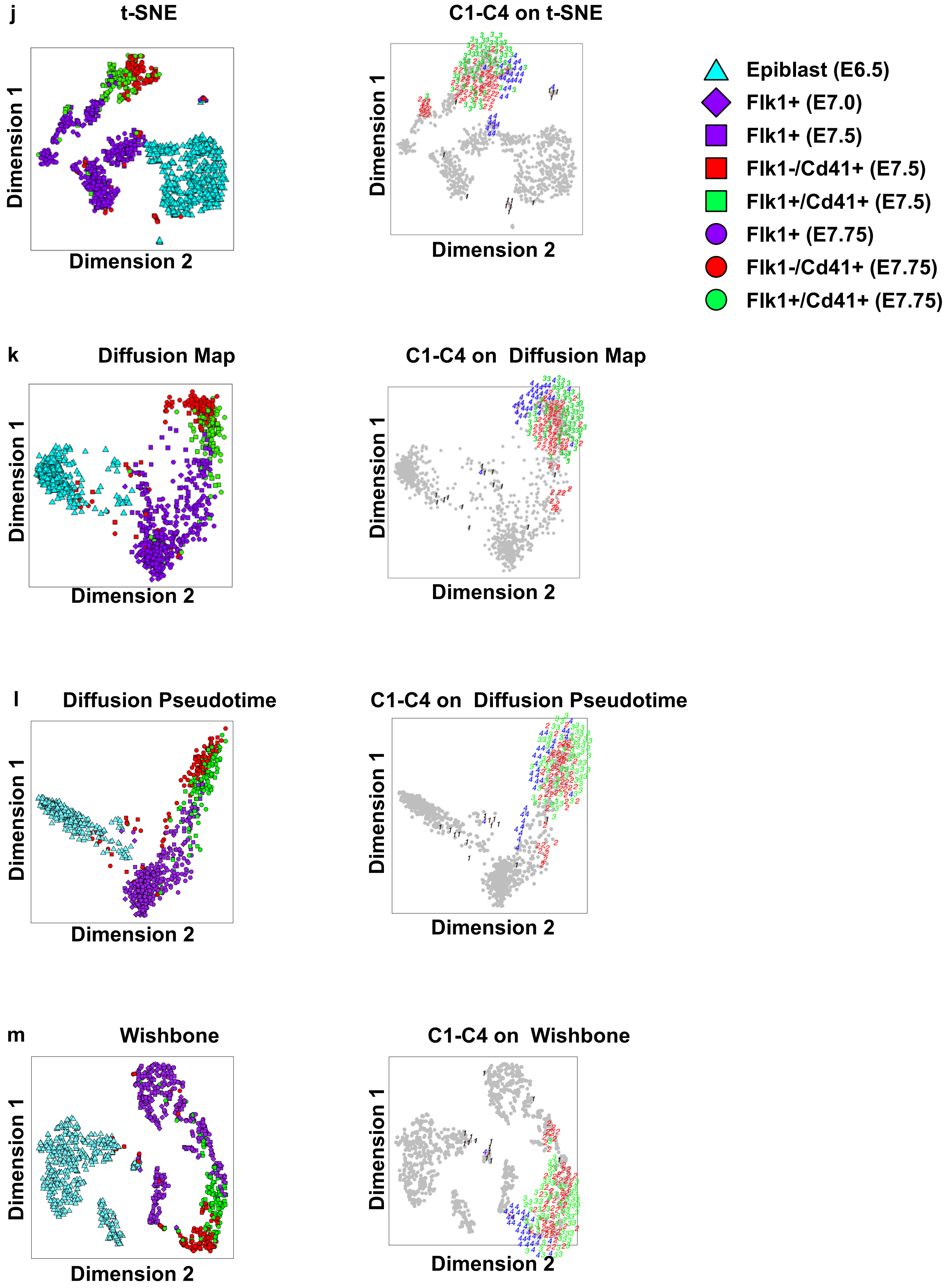

Supplementary Figure 11

n

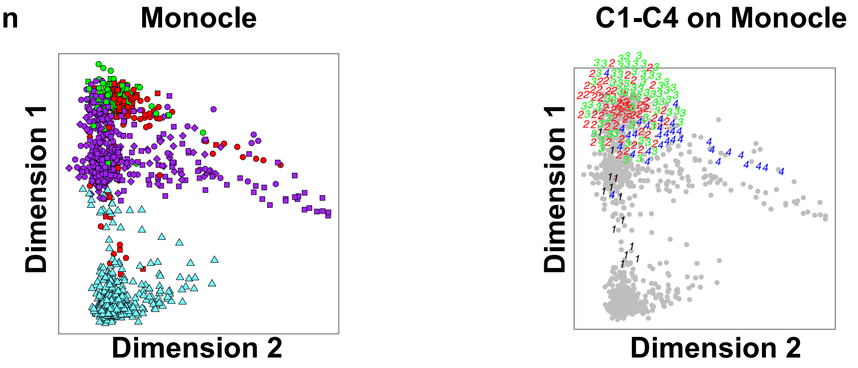

o

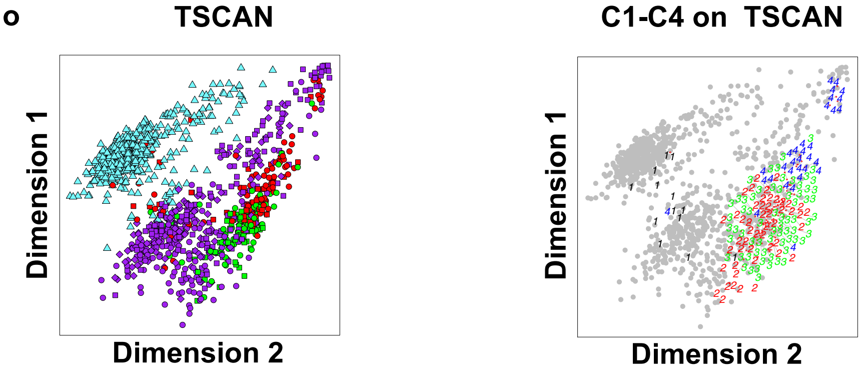

p

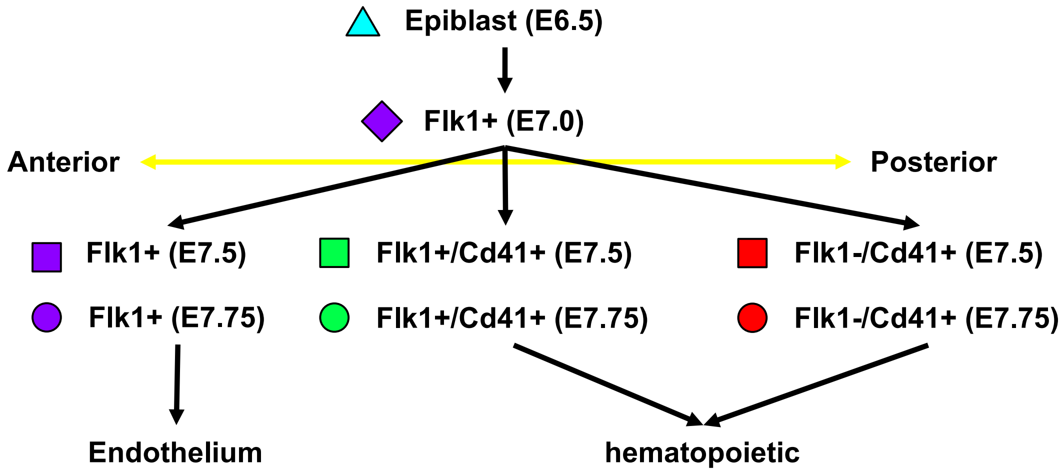

Supplementary Figure 11. TCM identifies four hematopoietic (Cd41$^+$) subpopulations of single cells from developmental day 7.75 (E7.75) of mouse mesodermal diversification (C1-C4). (a-c) On the latent space produced by TCM, the principal anterior-posterior axis is highlighted along the single cells captured at E7.5. The anterior-posterior axis is supported by the expression pattern of **(a)** the anterior marker Lefty2 and **(b)** the posterior marker Bmp4. **(c)** Erythroid-specific transcription factors Gata1 shows enriched expression in the posterior region. **(d-i)** The mesodermal gene Hand1/Fgfr1, the primitive erythrocyte marker Zfpm1/Gata2 and the hemoglobin gene Hbb-bh1/Hba-x show different expression patterns across four hematopoietic subpopulations (C1-C4), suggesting different developmental states and functional roles of the four subpopulations. **(j-o)** On the latent space produced by the other six methods, the four hematopoietic subpopulations from E7.75 are crowded together and indistinguishable due to the high temporal variance present in this dataset, which overwhelms the analysis. **(p)** A schematic of mouse mesodermal diversification from epiblast to endothelial and hematopoietic lineages. In boxplot, the upper whisker is located at the smaller of the maximum input value and Q_3+1.5*IQR, and the lower whisker is located at the larger of the smallest input value and Q_1-1.5*IQR, where Q_1 and Q_3 are the first and third quantile of the input data, and IQR = Q_3 - Q_1, the box length.

Supplementary Figure 12

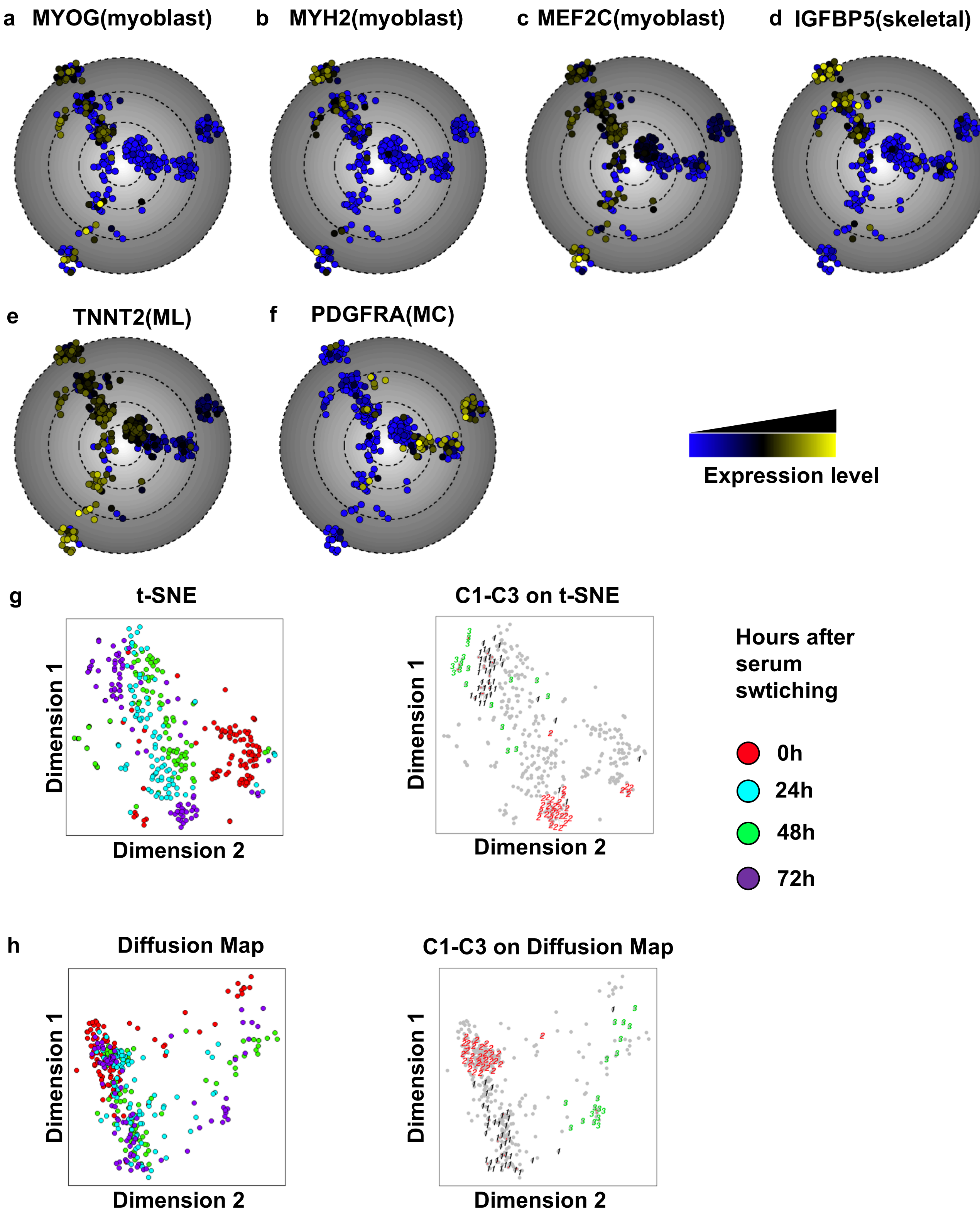

Supplementary Figure 12

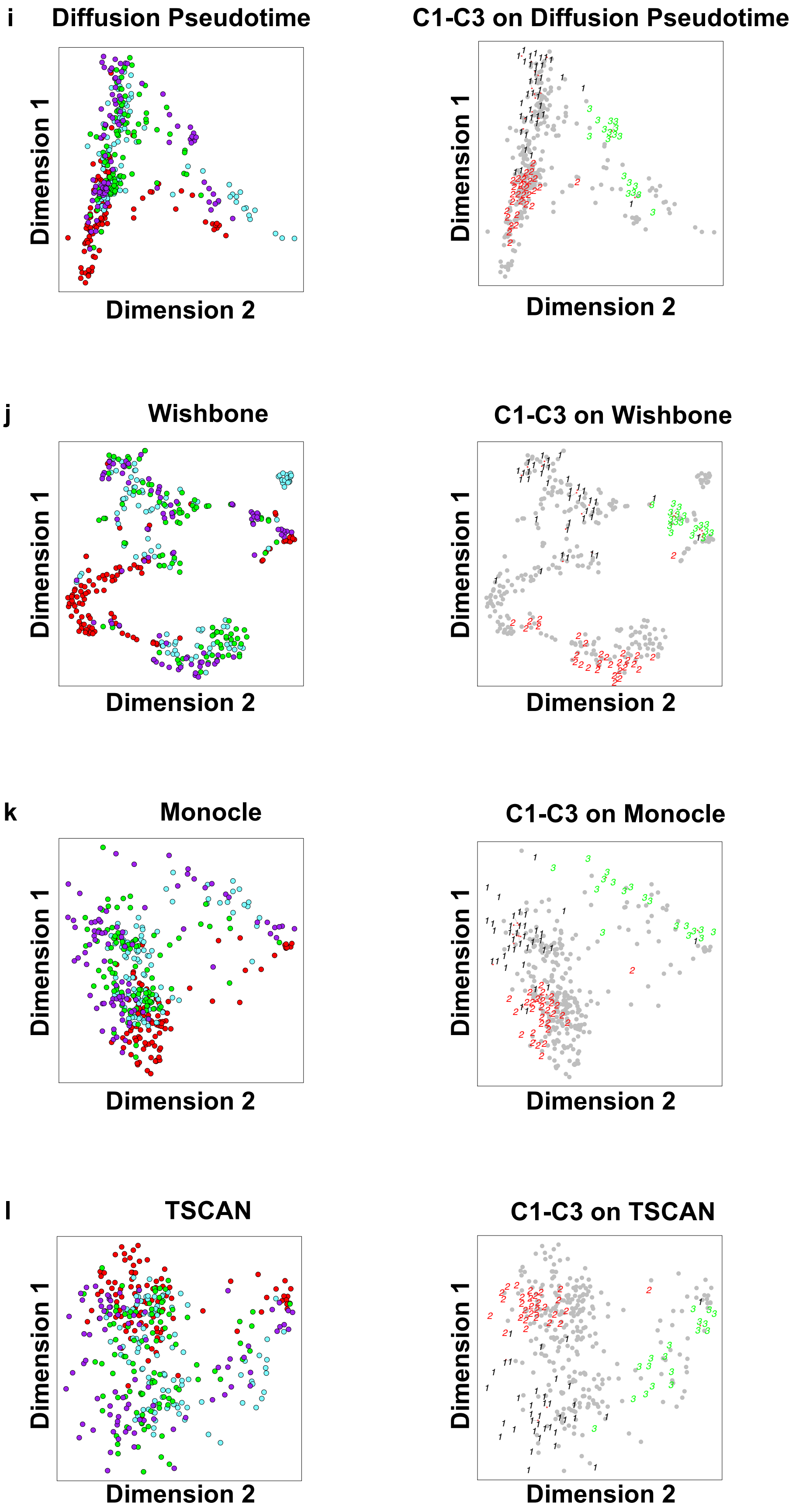

Supplementary Figure 12

m

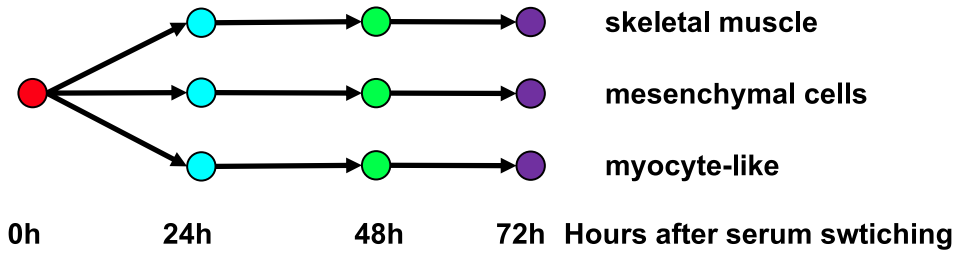

Supplementary Figure 12. TCM identifies three subpopulations from the 72 hour period following human myoblast differentiation. **(a-f)** TCM is used to visualize the single cell RNA-seq of the differentiation of human primary myoblasts, where the expression pattern of 372 single cells were profiled from 0, 24, 48, and 72 hours post-serum switching, respectively. TCM successfully identifies three distinct subpopulations from the last time point (72 hours). The expression pattern of lineage marker genes shows that the myoblast markers such as **(a)** MYOG, **(b)** MYH2 and **(c)** MEF2C are detected in both C1 and C3 populations, suggesting that both C1 and C3 represent the differentiated myoblast lineages from the 72 hour period. However, the **(d)** skeletal marker IGFBP5 and the **(e)** cardiac marker TNNT2 are differentially enriched in C1 and C3, respectively, suggesting that C1 and C3 represent two types of myoblast cell populations. The C2 population is the interstitial mesenchymal cells, due to the expression of **(f)** PDGFRA and the lack of expression of myogenic markers such as **(a)** MYOG and **(b)** MYH2. In comparison, **(g-l)** other methods either fail to identify C3 as a separate cell population, or to clearly separate the cells from different time points. Neither method preserves the lineage trajectories toward the three major terminal lineages at the 72 hour post-differentiation period. **(m)** A schematic of *in vitro* human myoblast differentiation.

Supplementary Figure 13

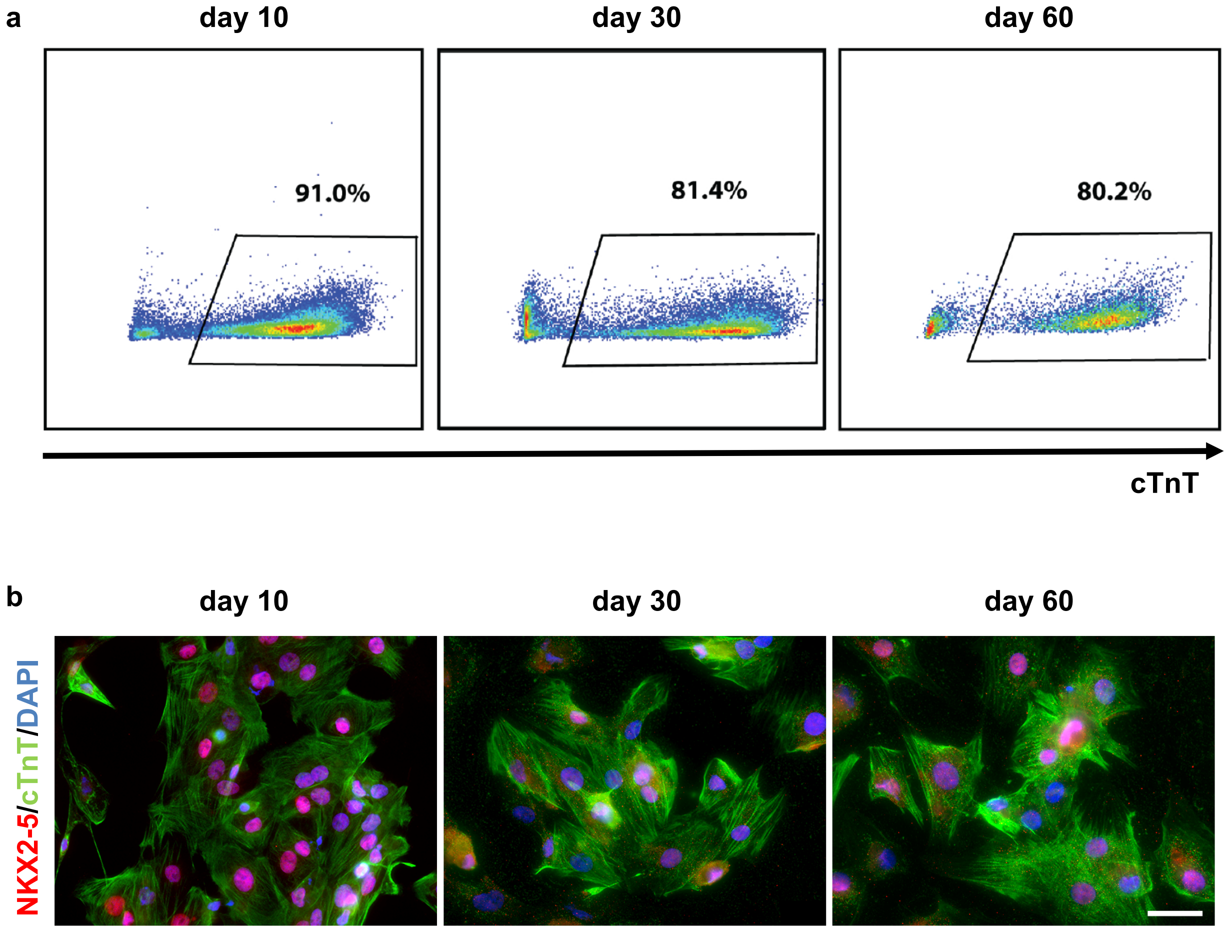

Supplementary Figure 13. The profiling of the global expression pattern of single cells from cardiomyocytes derived from human iPSC at differentiation day 0, 6, 10, 30 and 60. (a) The differentiation of hiPSCs towards the cardiomyocyte lineage was confirmed by FACS analysis using the cTnT antibody at days 10, 30 and 60. **(b)** Fluorescent immunohistochemistry of hiPSCs using NKX2-5 (red) and cTnT (green) antibodies. Nuclei are counterstained using DAPI (blue). Note that the cardiomyocyte markers, NKX2-5 and cTnT, are expressed from day 10 in the majority of cells, however cells continue to mature as seen by the changes in morphology. Scale bar: 200 μm.

Supplementary Figure 14

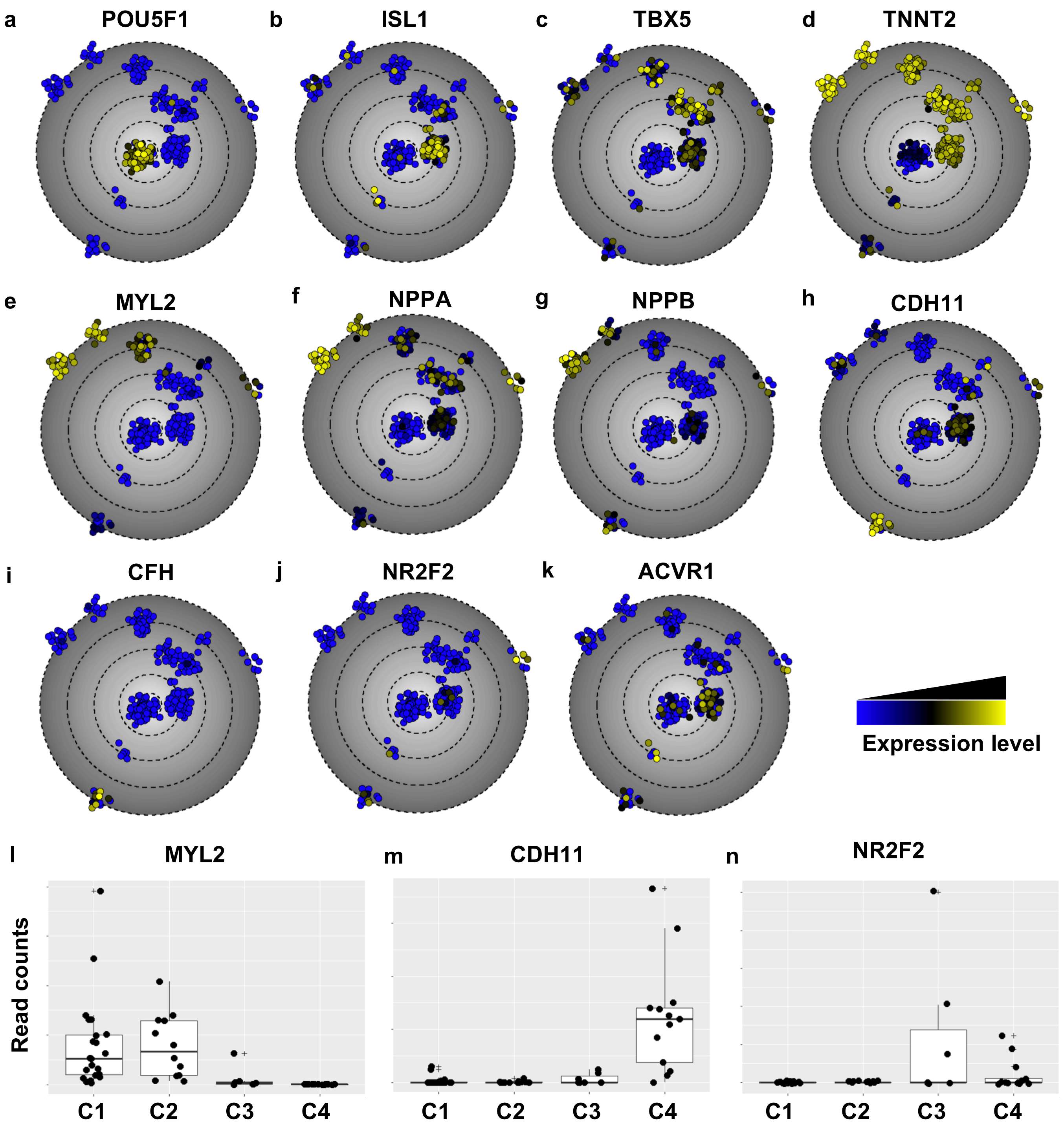

Supplementary Figure 14

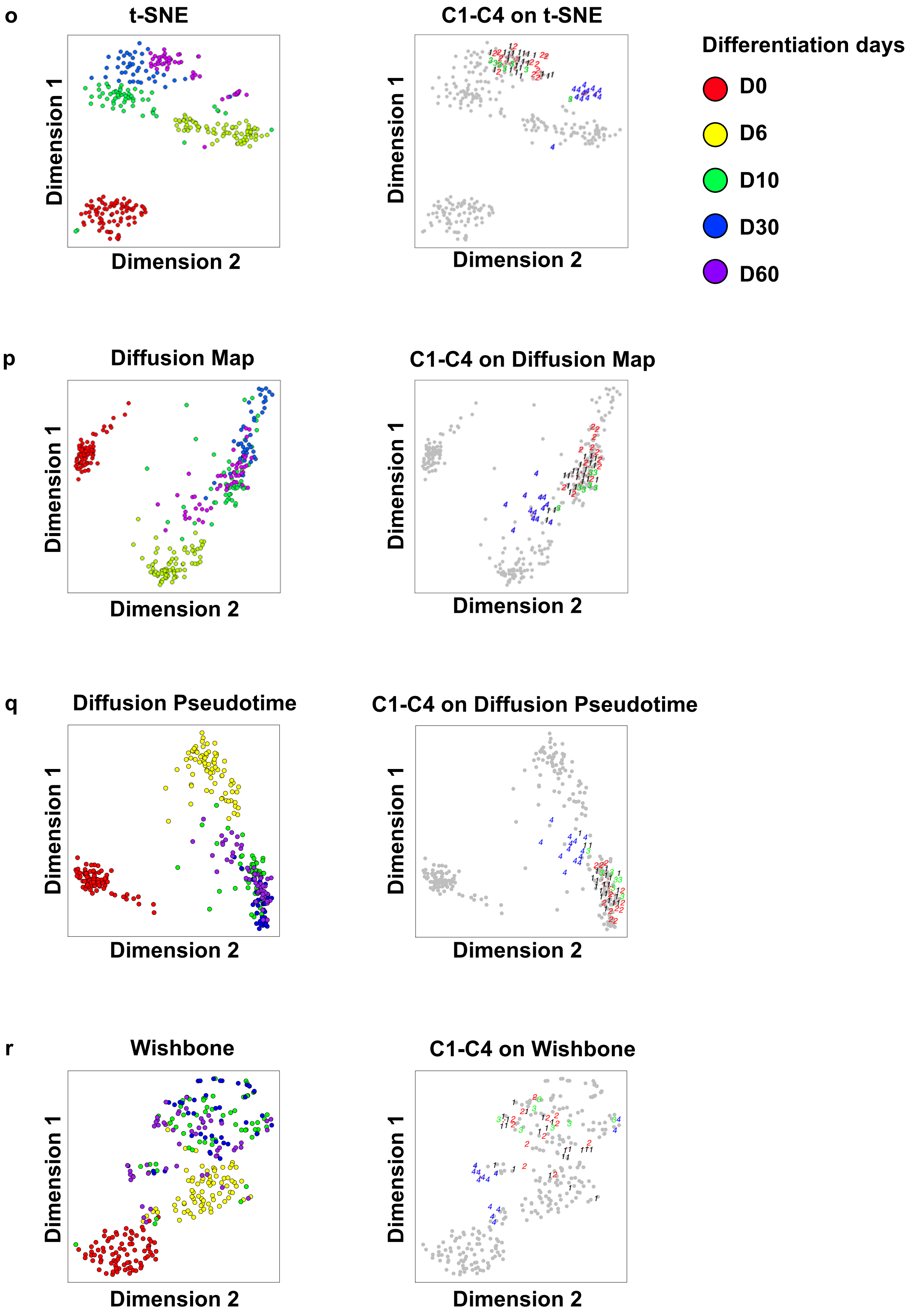

Supplementary Figure 14

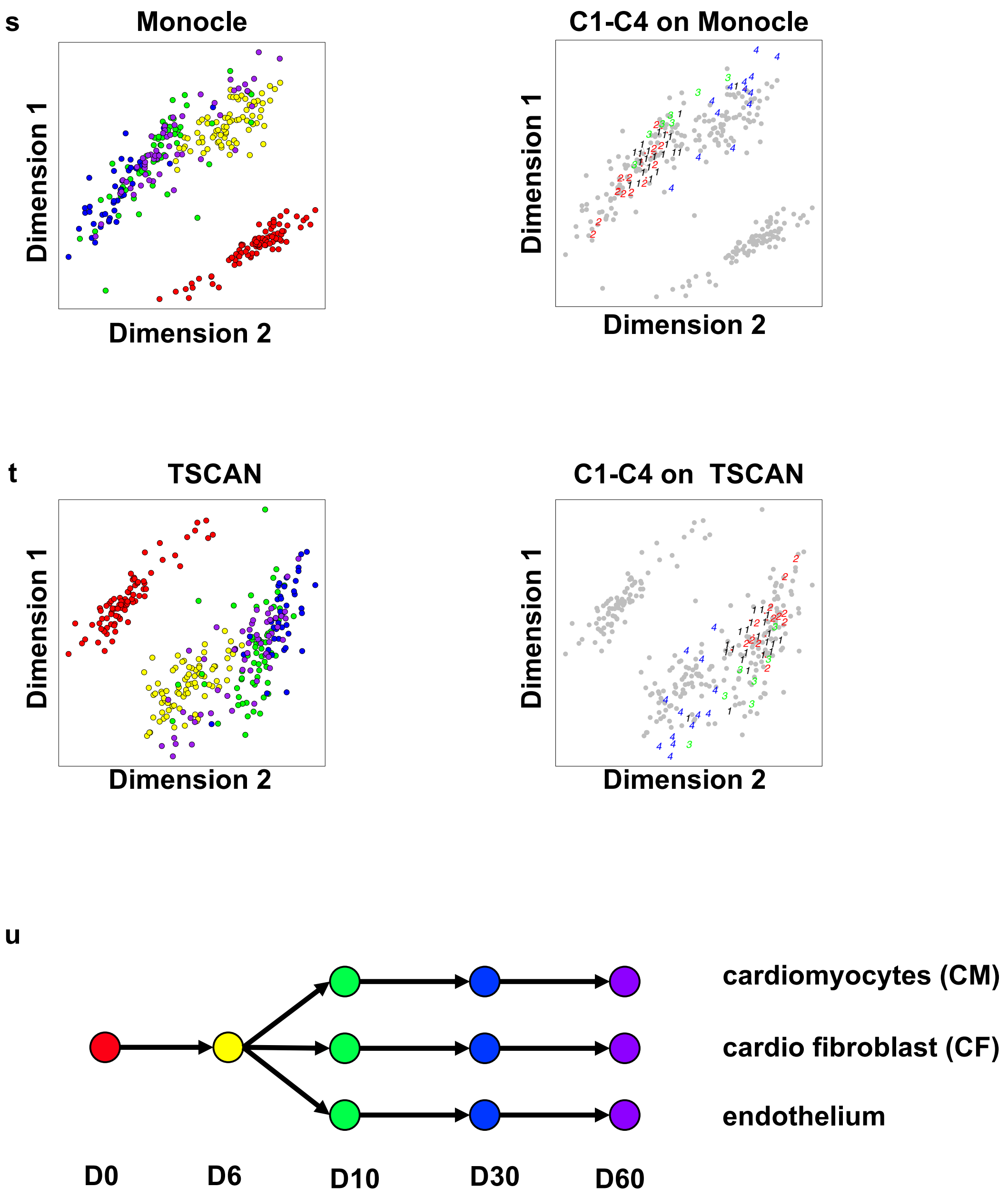

Supplementary Figure 14. TCM identifies four terminal subpopulations of single cells from differentiated human iPSC derived-cardiomyocytes (CM) (C1-C4) at Day 60. **(a-n)** The pluripotency marker (POU5F1), early cardiac development marker **(b)** ISL1 and **(c)** Tbx5 show dynamic expression pattern along the developmental trajectories on the latent space produced by TCM. **(d)** The cardiomyocyte marker TNNT2 shows elevated expression levels in both CM (C1 and C2) and endothelial populations (C3), while **(e and l)** MYL2 shows a more specific expression pattern in the dedicated CM populations (C1 and C2). Atrial genes such as **(f)** NPPA and **(g)** NPPB have higher expression levels in C1 than C2, suggesting diversification of CMs at day 60. The CF markers such as **(h and m)** CDH11 and **(i)** CFH show specific expression in the C4 population. The lymphatic endothelial markers such as **(j and n)** NR2F2 and **(k)** AVR1 show specific expression in the C3 population. **(o-t)** On the latent space produced by other methods, the three terminal subpopulations (C1- C3) from day 60 are crowded together and indistinguishable due to the high temporal variance present in this dataset. Though the C4 population (cardio-fibroblast) is separated from the remaining day 60 cells, there is no clear indication of the bifurcation of cardio-fibroblast (CF) cell populations from the CM (C1 and C2) and endothelial (C3) populations. **(u)** A schematic of human iPSC derived-cardiomyocyte (CM) differentiation. In boxplot, the upper whisker is located at the smaller of the maximum input value and Q_3+1.5*IQR, and the lower whisker is located at the larger of the smallest input value and Q_1-1.5*IQR, where Q_1 and Q_3 are the first and third quantile of the input data, and IQR = Q_3 - Q_1, the box length.

Supplementary Figure 15

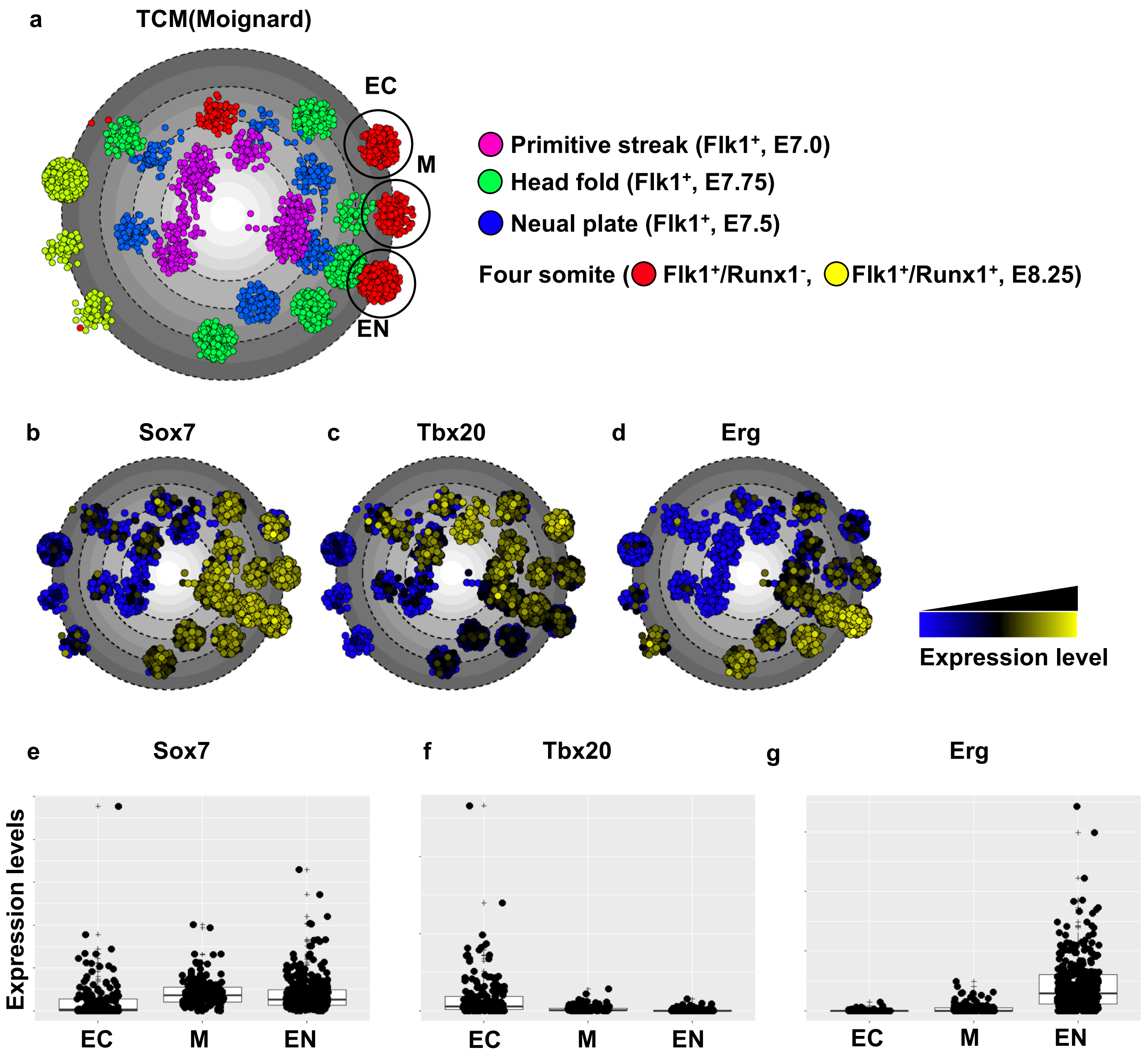

Supplementary Figure 15

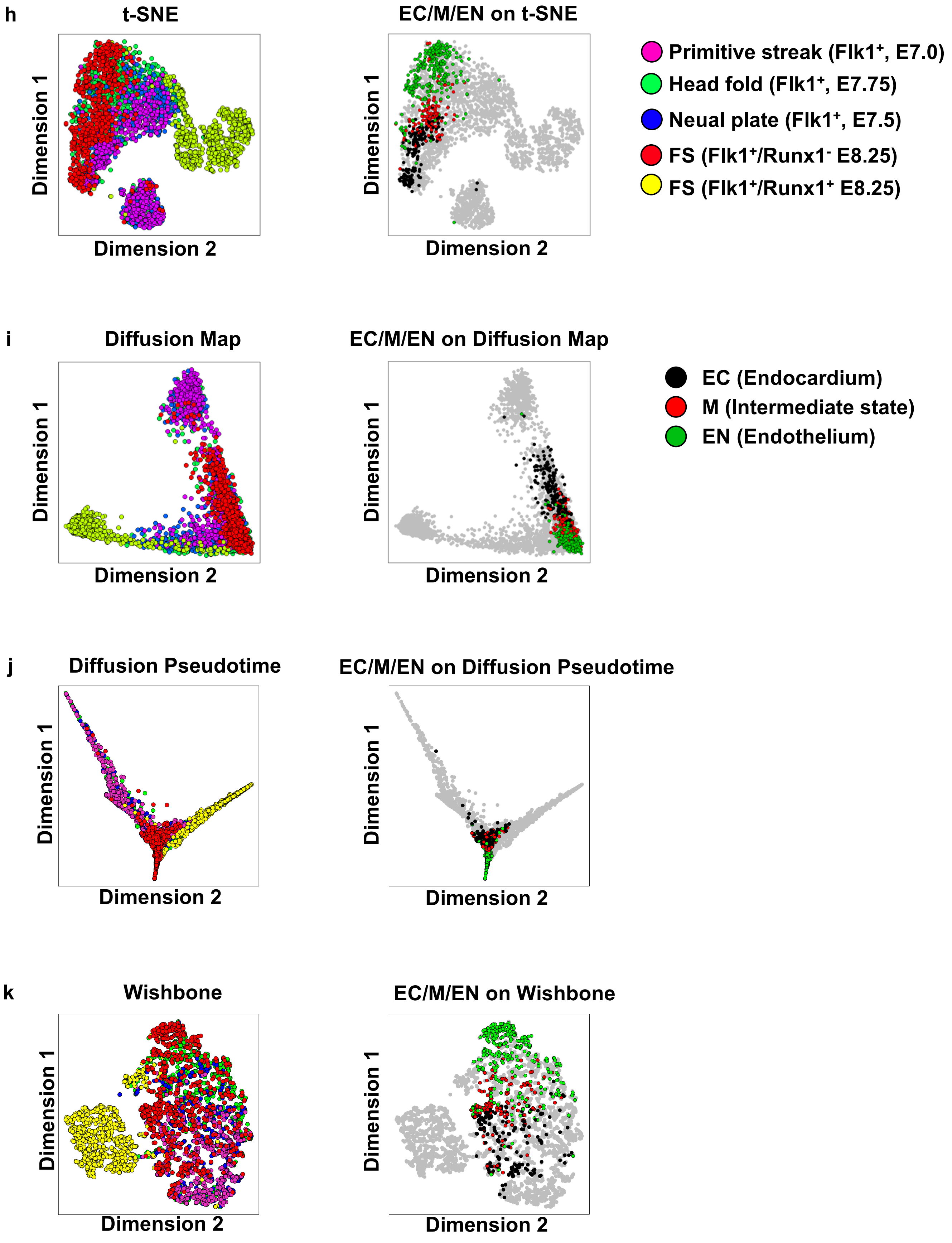

Supplementary Figure 15

I

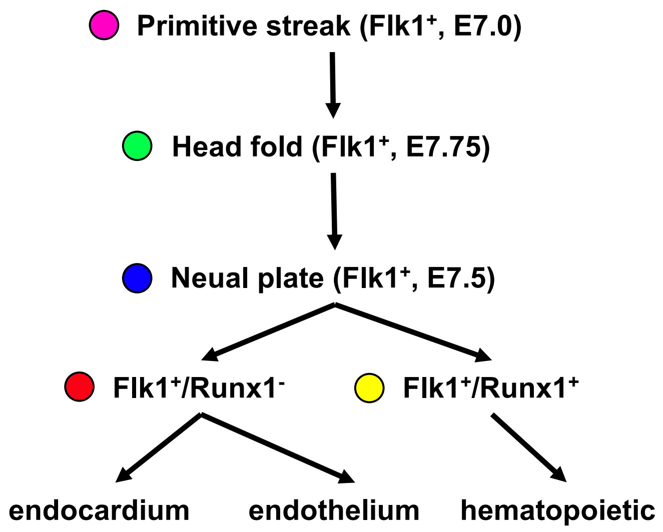

Supplementary Figure 15. TCM identifies three subpopulations from developmental day 8.25 (E8.25) of mouse mesodermal diversification. **(a)** TCM is used to visualize the single cell PCR of mouse mesodermal diversification, where the expression pattern of 3,934 single cells were profiled from the following developmental stages: primitive streak ($Flk1^+$, E7.0), neural plate ($Flk1^+$, E7.5), head fold ($Flk1^+$, E7.75) and four somite ($Flk1^+/Runx1^-$ and $Flk1^+/ Runx1^-$, E8.25) stage(s), respectively. TCM successfully identifies the major bifurcation of hematopoietic ($Flk1^+/Runx1^+$) and non-hematopoietic ($Flk1^+/Runx1^-$) lineages, and especially the subpopulations of endothelium (EN), endocardium (EC) and an intermediate state (M) that characterizes the expression signature of both EN and EC, from the non-hematopoietic $Flk1^+/Runx1^-$ cells from the terminal time point E8.25 (red cells). **(b and e)** The early endothelial lineage marker Sox7 is detected in both EN and EC subpopulations, but has the highest expression in the intermediate state (M). The identification of EC and EN subpopulations are supported by the expression pattern of the **(c and f)** endocardial lineage marker Tbx20 and **(d and g)** endothelial lineage marker Erg. In comparison, **(h-k)** other methods fail to visually separate these three populations. **(l)** A schematic of mouse mesodermal diversification. In boxplot, the upper whisker is located at the smaller of the maximum input value and Q_3+1.5*IQR, and the lower whisker is located at the larger of the smallest input value and Q_1-1.5*IQR, where Q_1 and Q_3 are the first and third quantile of the input data, and IQR = Q_3 - Q_1, the box length.

Supplementary Figure 16

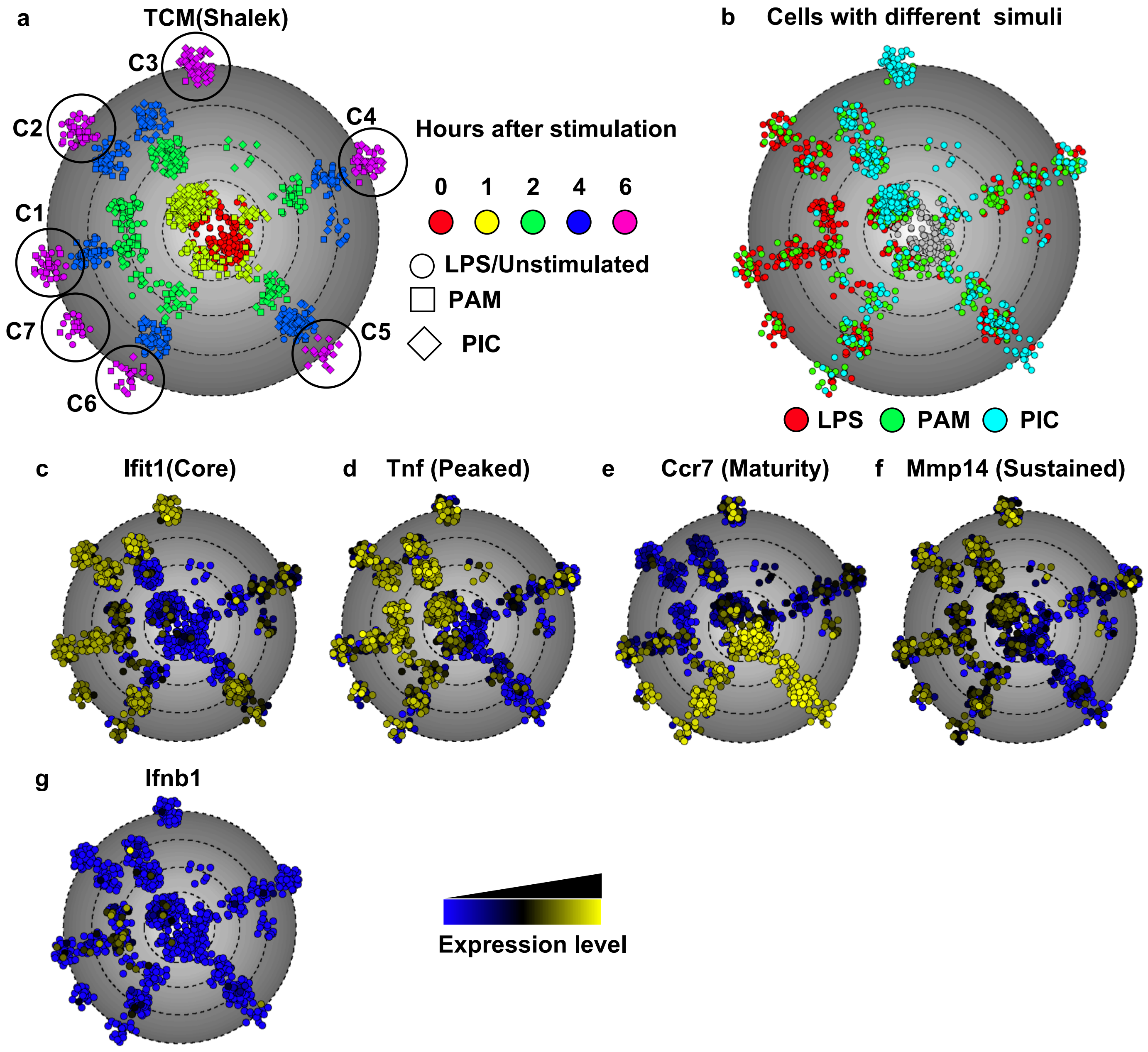

Supplementary Figure 16

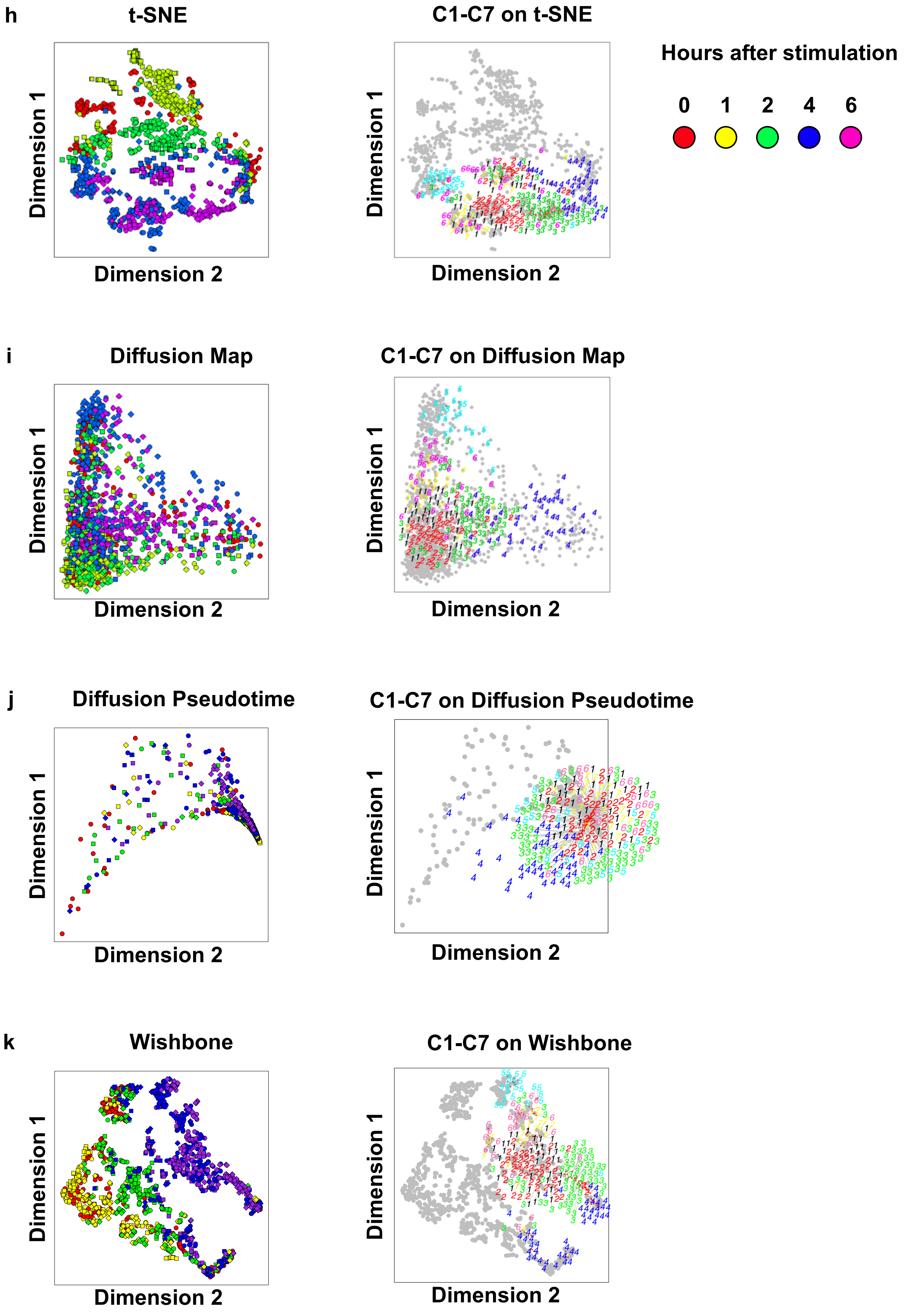

Supplementary Figure 16

l

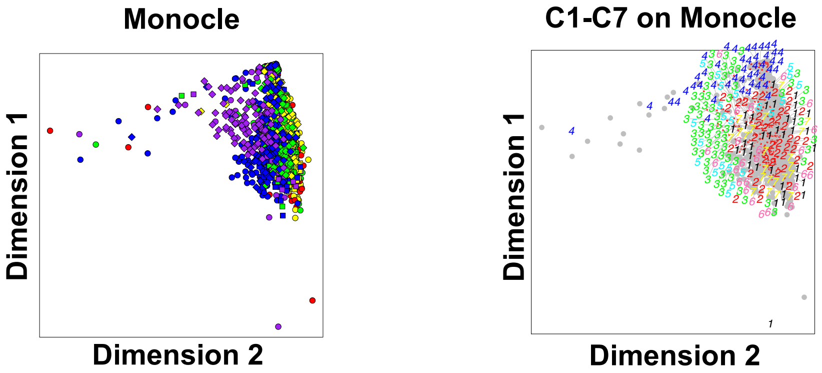

m

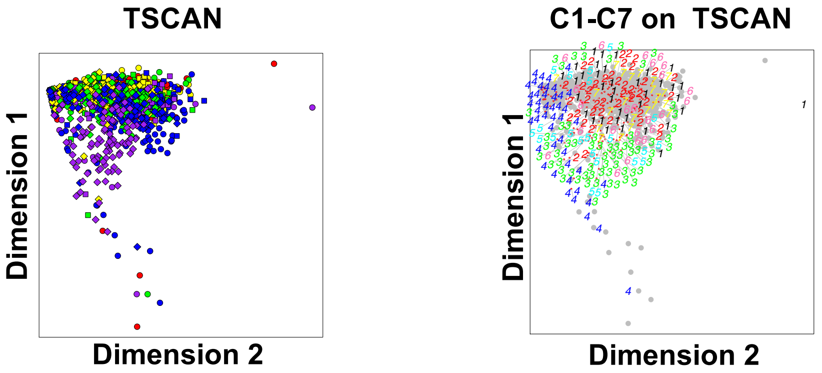

n

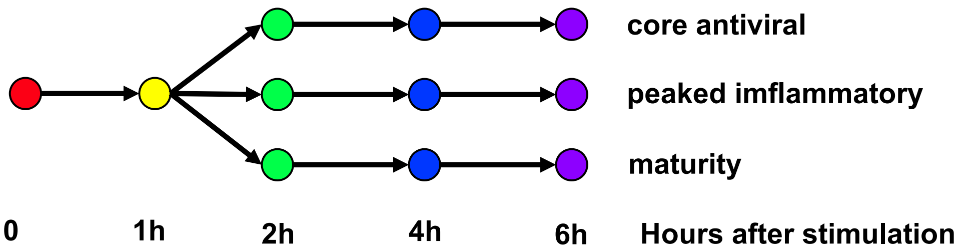

Supplementary Figure 16. TCM identifies seven subpopulations from six hours post stimulation of primary mouse bone-marrow-derived dendritic cells. (a and b) TCM is used to visualize the scRNA-seq of dendritic cells stimulated with three pathogenic components: LPS, PIC (viral-like double-stranded RNA), and PAM (synthetic mimic of bacterial lipopeptides), where the expression pattern of 1,378 single cells were profiled from 0, 1, 2, 4, 6 hours post-stimulation. TCM successfully identifies the subpopulations where **(c)** the core antiviral gene Ifit1 (enriched in all sub-populations), **(d)** peaked inflammatory gene Tnf (down-regulated in C5), **(e)** maturity gene Ccr7 (down-regulated in C2 and C4), **(f)** sustained inflammatory gene Mmp14 (down-regulated in C5), and **(g)** Ifnb1 (absent in all terminal subpopulations) show dynamic expression pattern over seven terminal populations. In comparison, **(h-m)** other methods fail to reveal the major developmental trajectories and fail to separate the terminal subpopulations. **(n)** A schematic of differentiation of primary mouse bone-marrow-derived dendritic cells.

Supplementary Figure 17

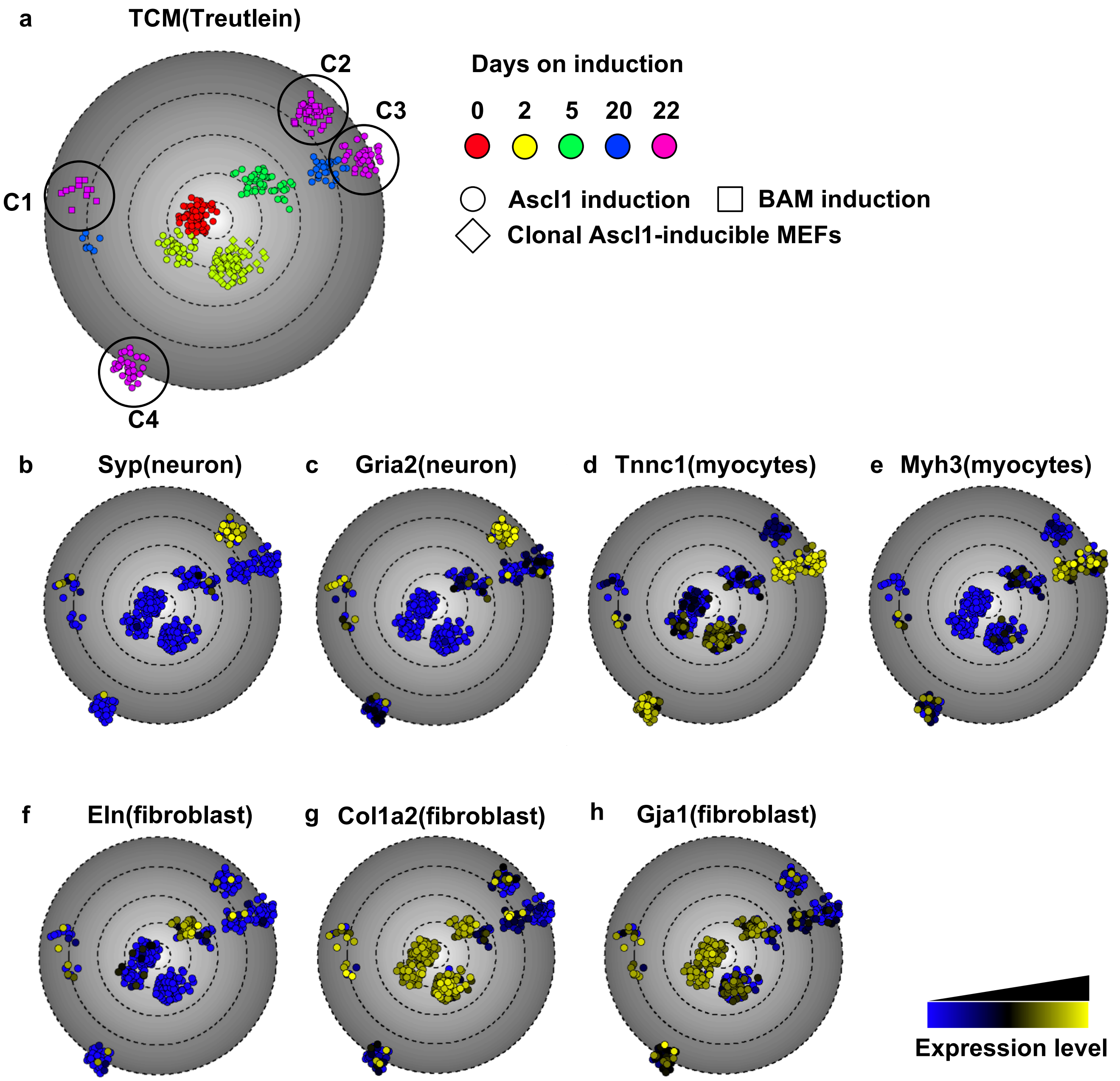

Supplementary Figure 17

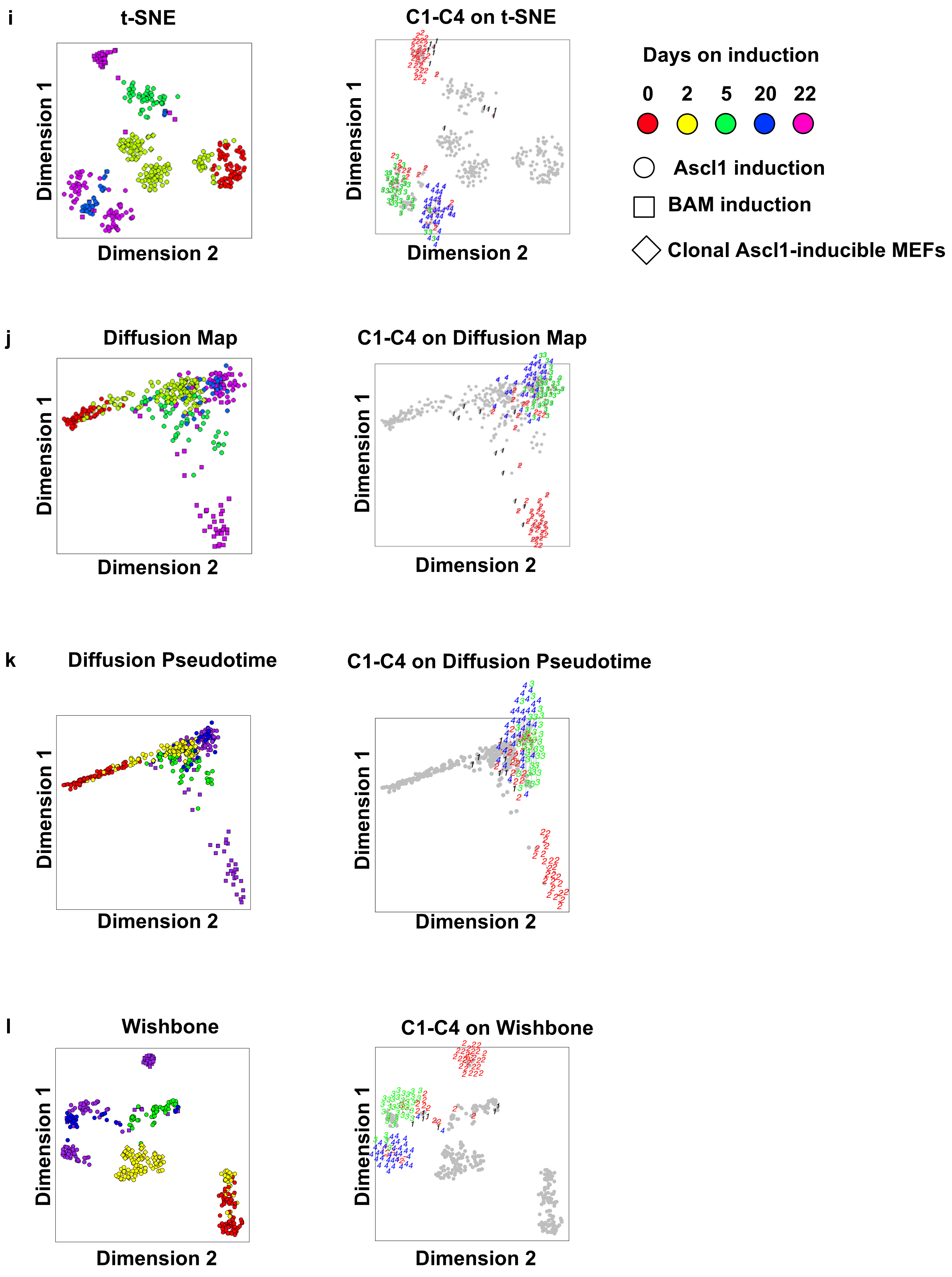

Supplementary Figure 17

m

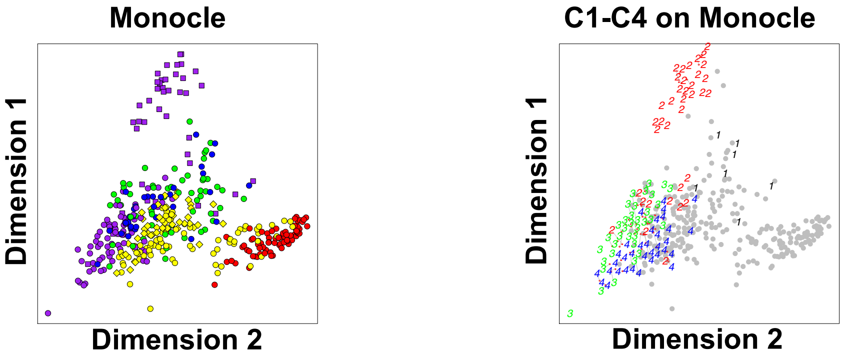

n

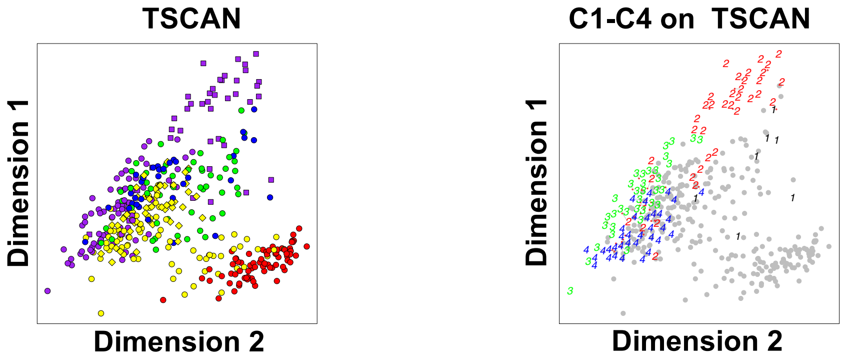

o

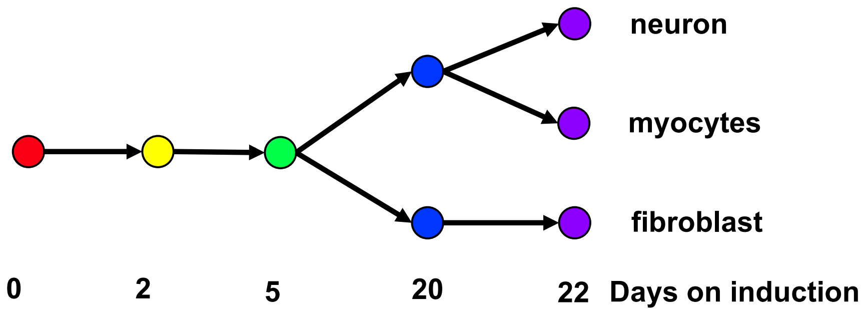

Supplementary Figure 17. TCM identifies four subpopulations of cells following the direct reprogramming of mouse fibroblasts to neurons (at day 22). **(a)** TCM is used to visualize the single cell RNA-seq of direct reprogramming from mouse fibroblasts to neurons, where the expression pattern of 405 single cells were profiled from 0, 2, 5, 20, and 22 days post reprogramming, respectively. TCM successfully identifies four subpopulations of day 22 cells (C1-C4). The expression pattern of lineage marker genes shows that neuron marker genes **(b)** Syp and **(c)** Gria2 are strongly expressed in C2, suggesting that C2 represents the dedicated neural lineage. Myocyte markers show more dynamic expression patterns: **(d)** Tnnc1 is enriched in both C3 and C4, while myocyte markers such as **(e)** Myh3 is more enriched in C3. In contrast, the fibroblast markers such as **(f)** Eln, **(g)** Col1a2 and **(h)** Gja1 are more enriched in C4. These results suggest that C3 is committed to the myocyte lineage while C4 is in an intermediate state where both myocyte and fibroblast genes are co-expressed. The majority of cells from the C1 population come from the cells of BAM mediated reprogramming, and co-express neuronal (Gria2) and fibroblast markers (Col1a2 and Gja1), suggesting C1 is also a cell population in an intermediate cellular state. In comparison, **(i-n)** other methods fail to recover the major developmental trajectories during the reprogramming process, and fail to visually identify the C1 population where fibroblast and neuro markers are co-expressed. **(o)** A schematic of direct reprogramming of mouse fibroblasts to neurons.

Supplementary Figure 18

a

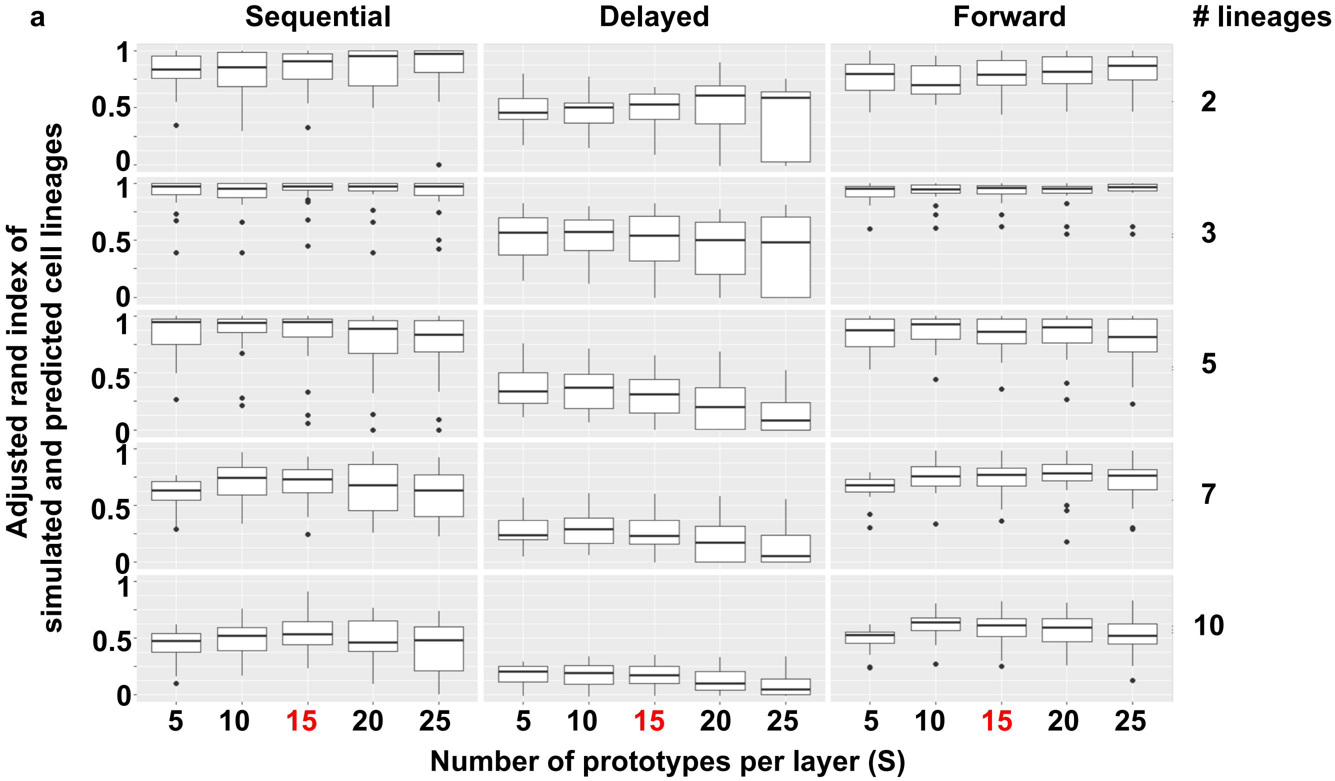

b

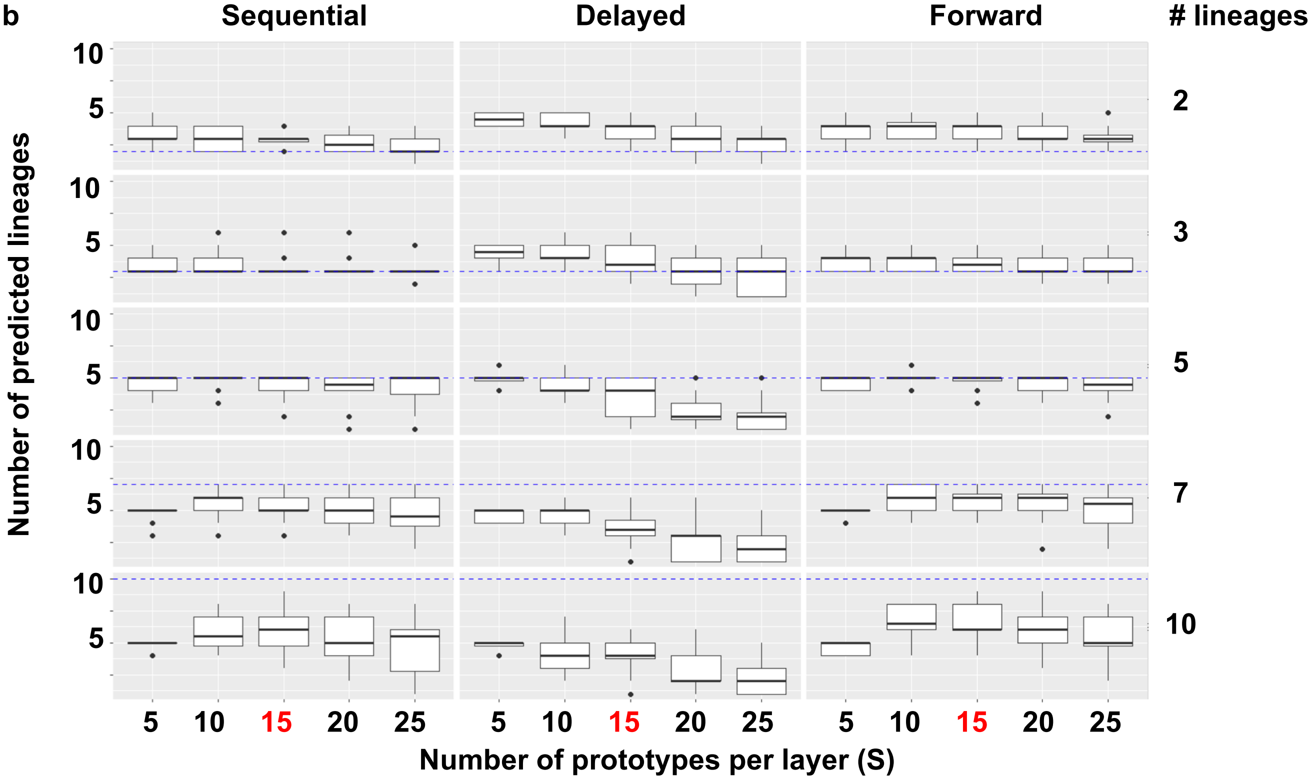

Supplementary Figure 18

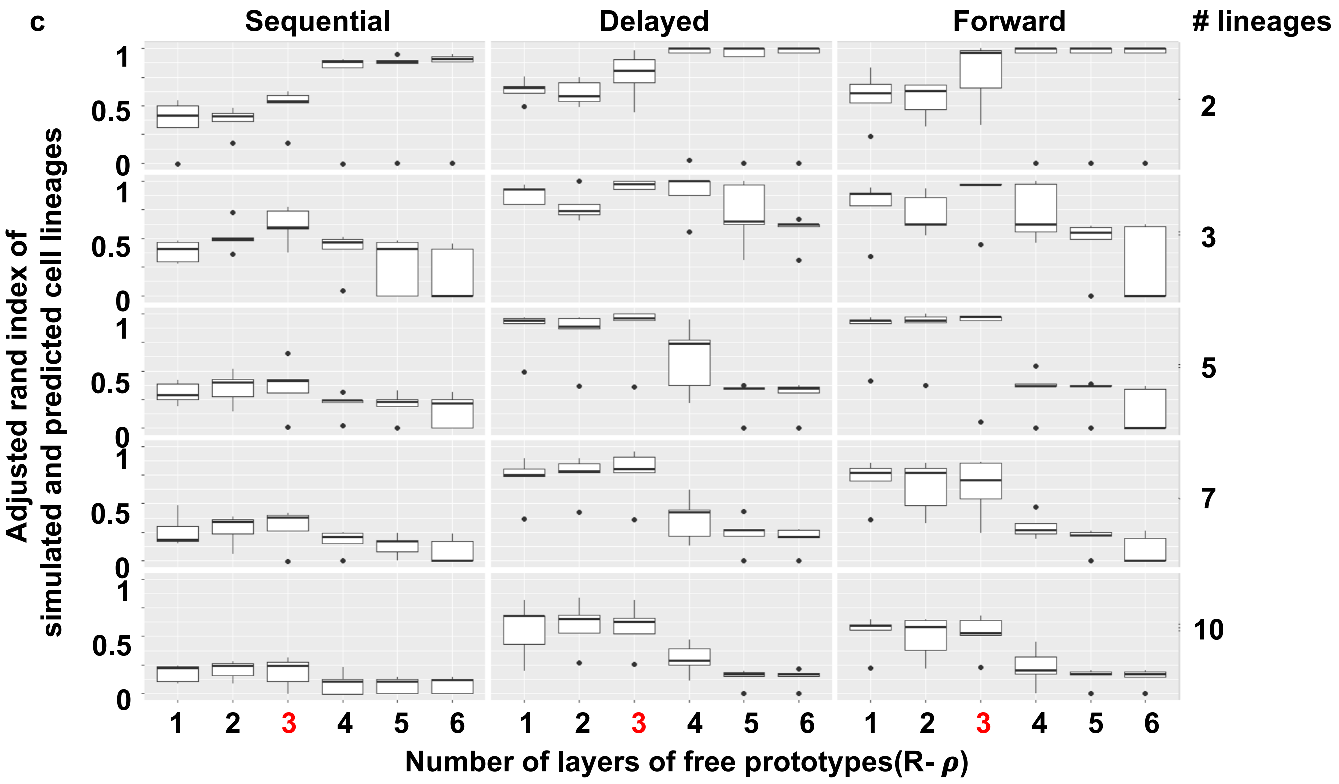

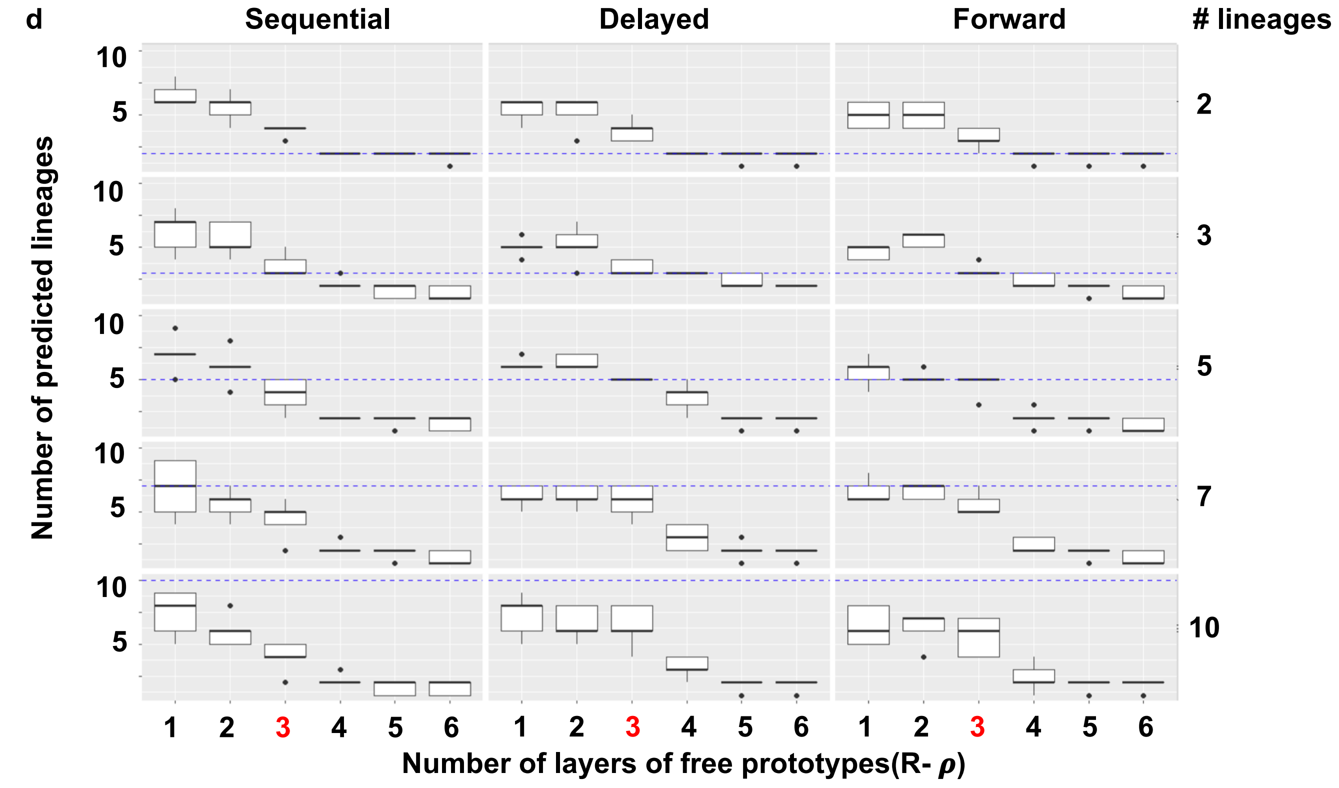

Supplementary Figure 18

e

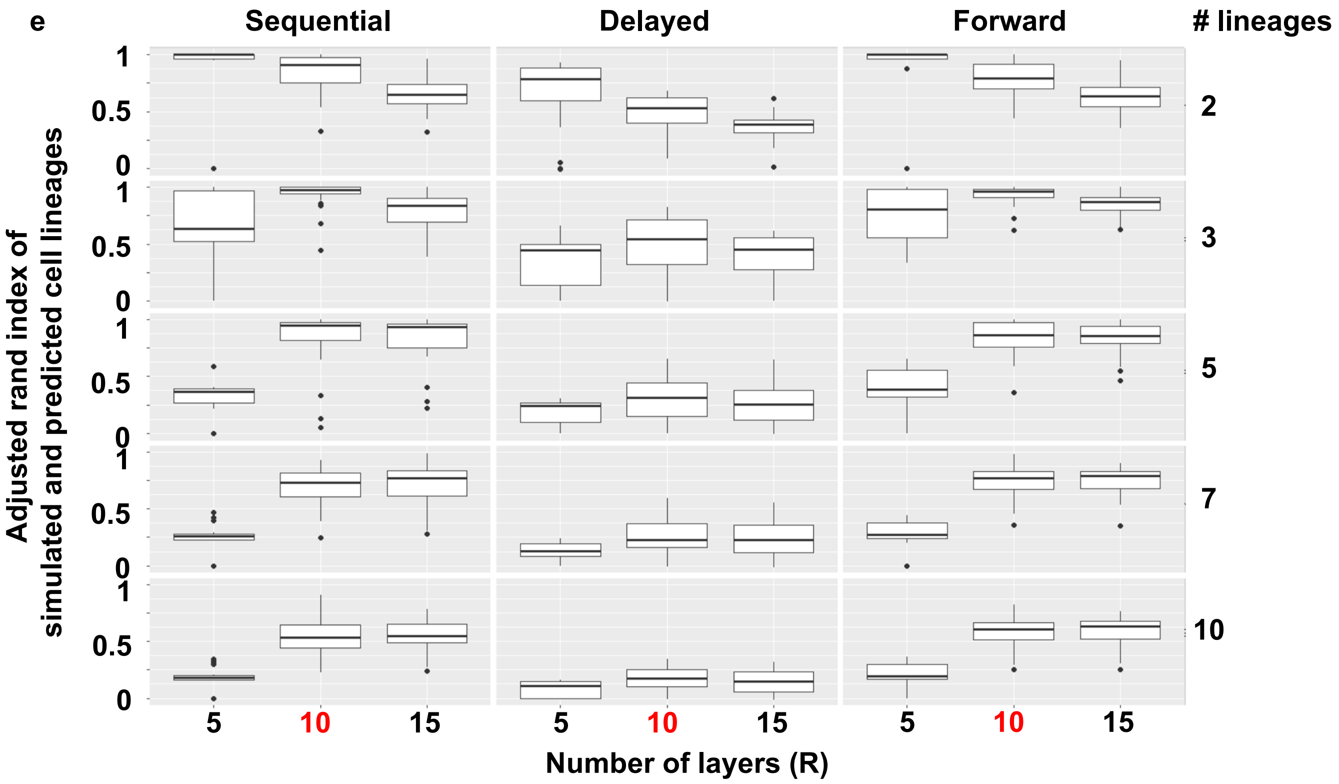

f

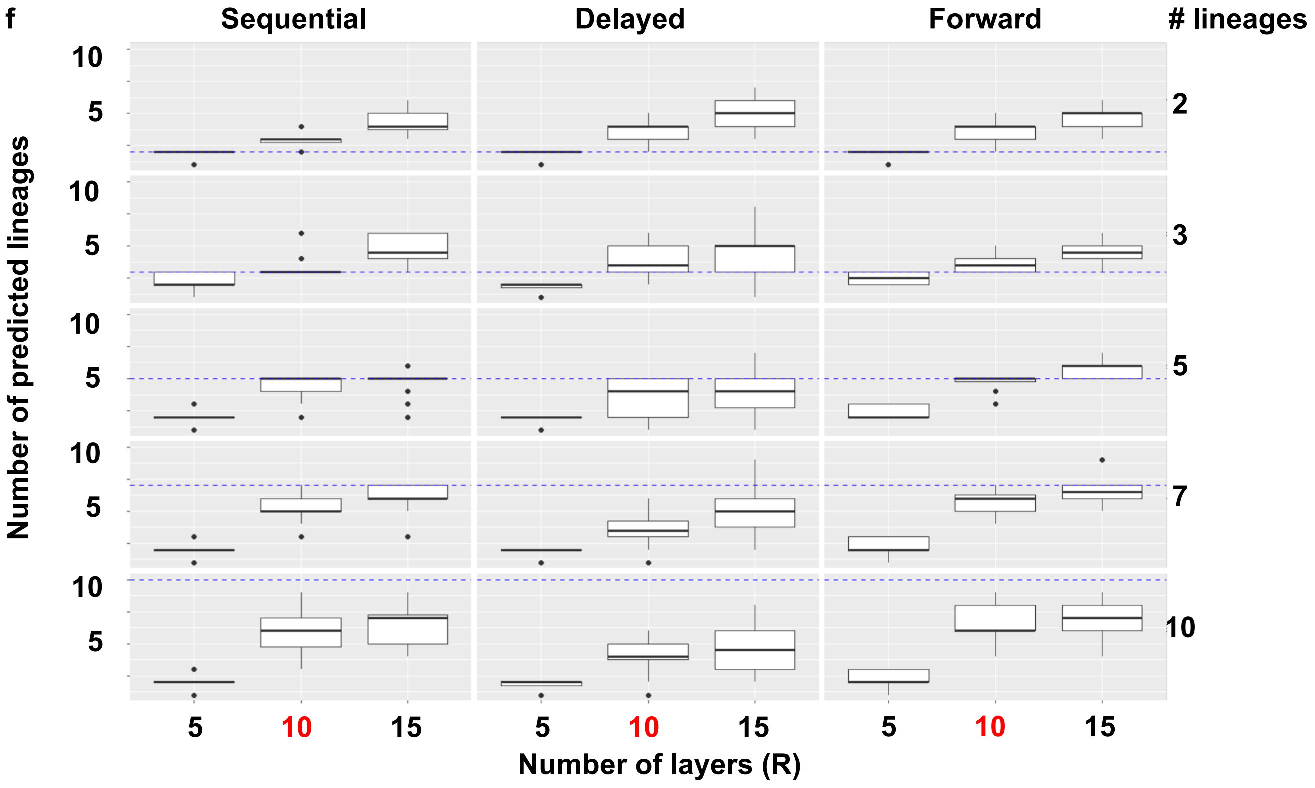

Supplementary Figure 18. The simulation studies determined the optimal prototypes per layer ($\boldsymbol{S = 15}$), the number of layers of free prototypes ($\boldsymbol{\rho}$), the convolving prototypes ($\boldsymbol{R - \rho = 3}$), and the number of layers ($\boldsymbol{R = 10}$). **(a-b)** 15 prototypes per layer ($S = 15$), three layers of convolving prototypes ($\mathrm{R} - \rho = 3$) and 10 layers per time points ($\mathrm{R} = 10$) had generally the best performance for the visualization of the simulated temporal scRNA-seq dataset with three different differentiation models (sequential, delayed and forward cell sampling) and multiple lineages (2, 3, 5, 7 and 10). The performance was evaluated at two metrics **(a,c and e)** including the adjusted Rand Index (ARI) between simulated and predicted cell lineages, and **(b,d and f)** the number of predicted lineages (among the cells from the last time point). In boxplot, the upper whisker is located at the smaller of the maximum input value and Q_3+1.5*IQR, and the lower whisker is located at the larger of the smallest input value and Q_1-1.5*IQR, where Q_1 and Q_3 are the first and third quantile of the input data, and IQR = Q_3 - Q_1, the box length.

Supplementary Figure 19

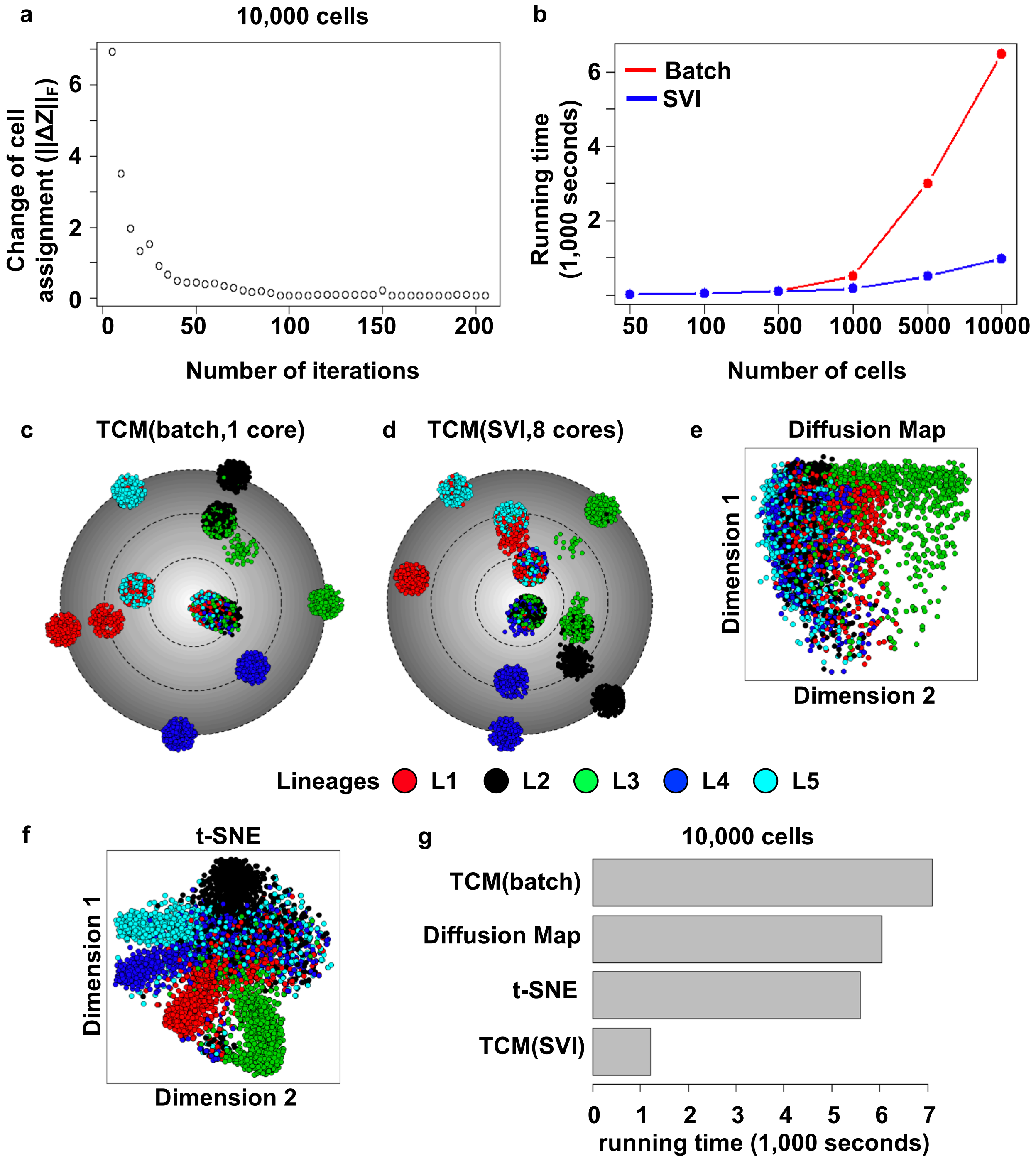

Supplementary Figure 19. TCM visualized large scale temporal scRNA-seq using stochastic variational inference (SVI) based optimization. (a) The convergence of the change of cell assignment to the prototypes ($\|\mathbf{\Delta Z}\|_F$) using TCM on a simulated temporal scRNA-seq dataset of 10,000 cells. **(b)** The computational time for running TCM on simulated datasets ($N = 10{,}000, S = 15, R - \rho = 3, R = 10$), using batch and SVI optimization, respectively. **(c-d)** TCM with SVI optimization achieved a similar performance regarding the separation of lineages compared to TCM with batch optimization ($N = 10{,}000, M = 10{,}000$). **(e-f)** The performance of diffusion map and t-SNE of the synthetic scRNA-seq datasets of 10,000 cells. **(g)** The running time of TCM with batch optimization, TCM with SVI optimization, t-SNE (*Rtsne* in R *Rtsne* package) and diffusion map (*diffuse* in R *diffusionMap* package) on synthetic datasets with 10,000 cells.

Supplementary Figure 20

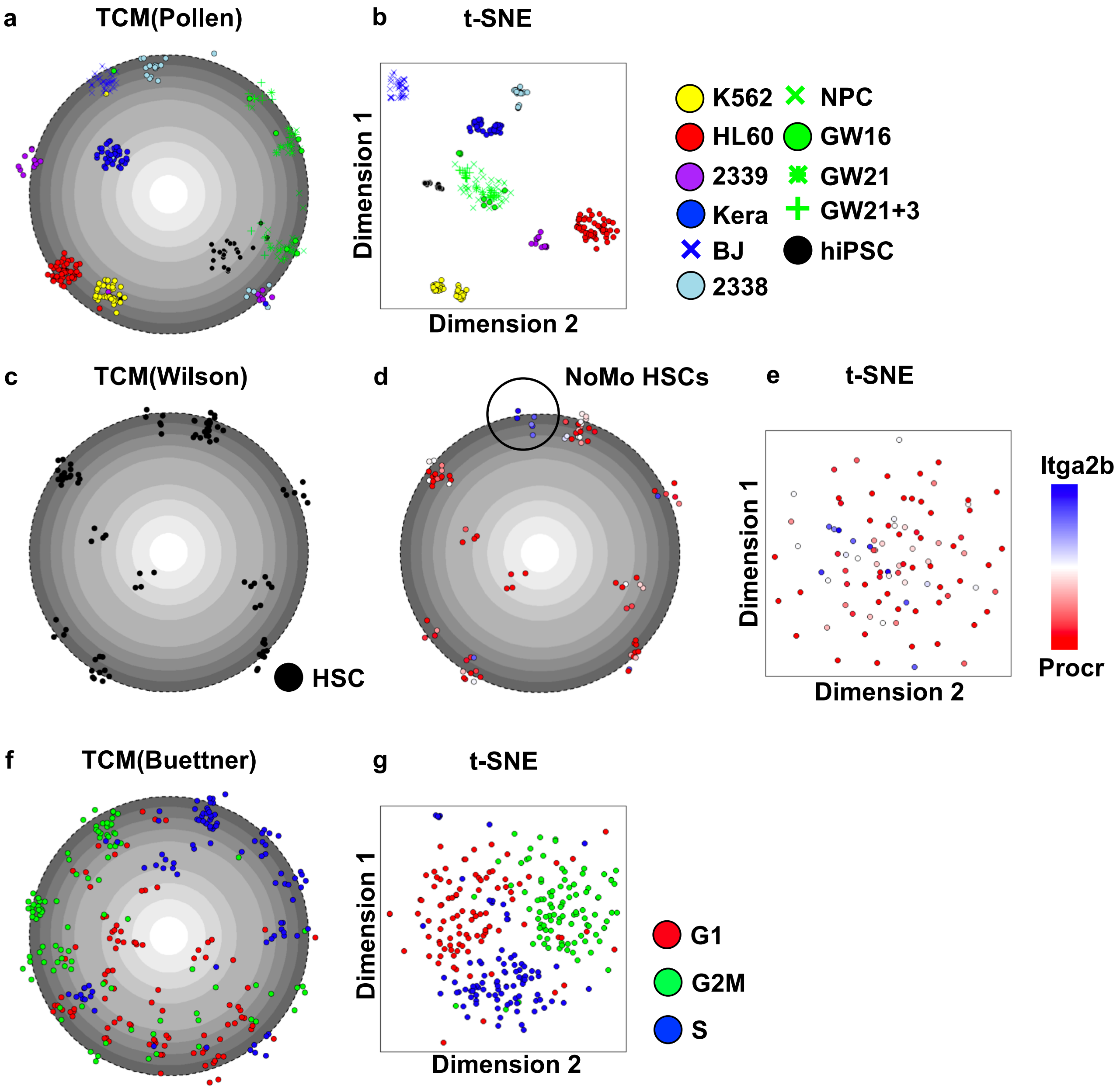

Supplementary Figure 20. Non-temporal TCM (nt-TCM) was able to reveal subpopulations of cells using non-temporal scRNA-seq datasets. The visualization of scRNA-seq datasets with 11 independent cell types using **(a)** nt-TCM and **(b)** t-SNE algorithms. The visualization of hematopoietic stem cells (HSCs) using **(c-d)** nt-TCM and **(e)** t-SNE. **(d)** nt-TCM was able to identify a separate sub-population of cells showing a relatively high expression of Itga2b and a low expression of Procr compared with the remaining cells. The visualization of mESCs with three different cell cycle stages (G1, S and G2M) using **(f)** nt-TCM and **(g)** t-SNE. Cells from different G1, G2M and S stages were colored red, green and blue, respectively.

Supplementary Figure 21

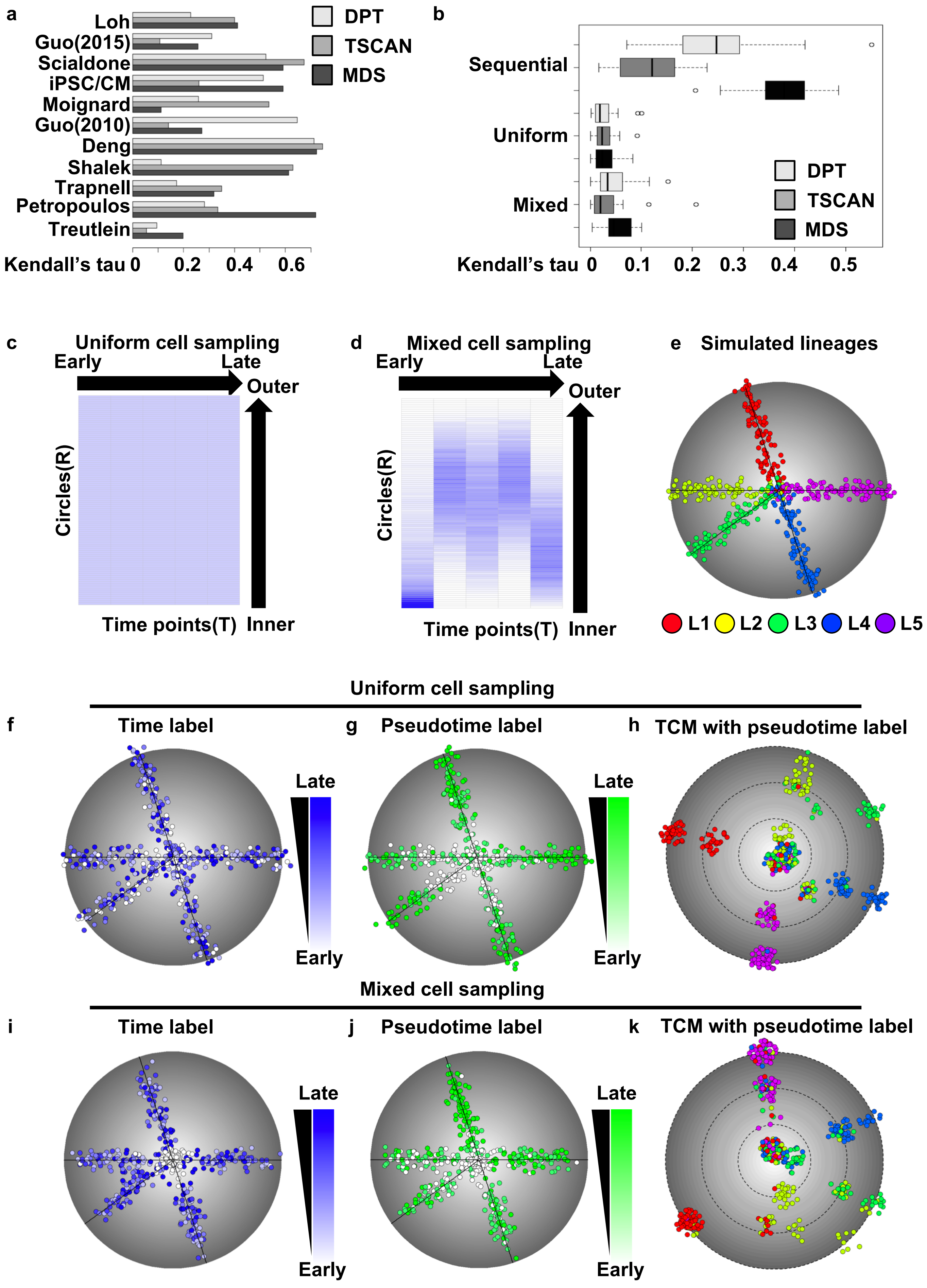

Supplementary Figure 21

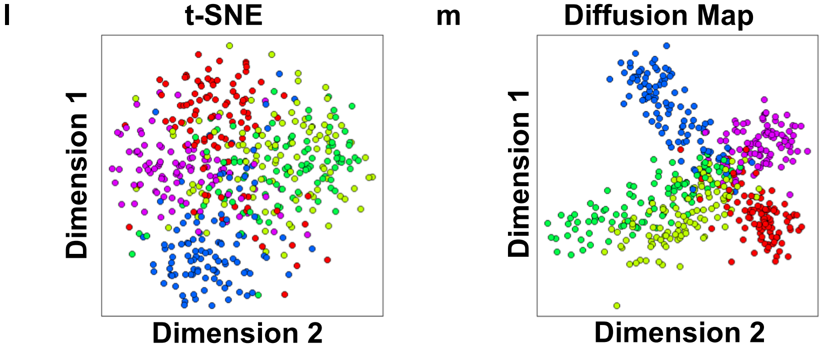

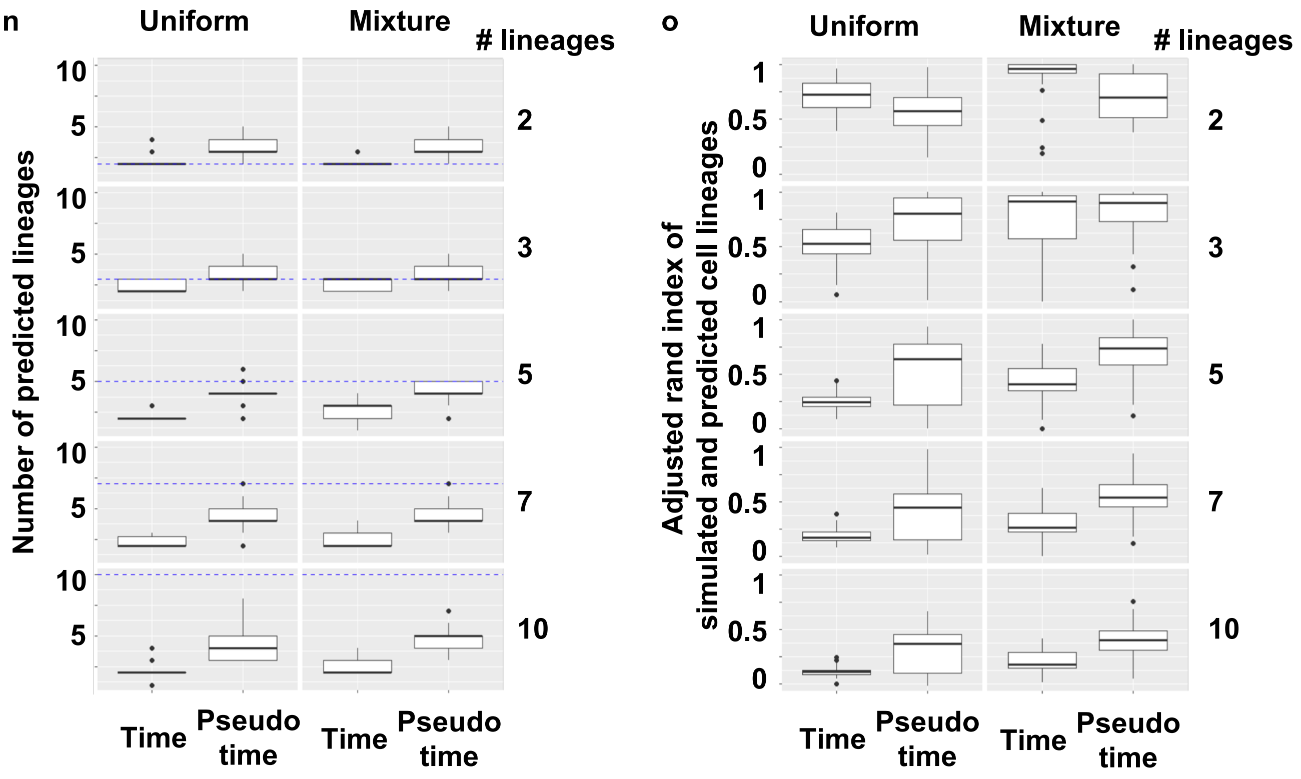

Supplementary Figure 21. TCM visualized highly asynchronized or random scRNA-seq datasets using pseudotime labels. **(a)** The Kendall rank correlation coefficients between the known time labels and the inferred pseudotime for 11 real temporal scRNA-seq datasets. The pseudotime indices were inferred using diffusion pseudotime (DPT), TSCAN and a MDS based method. **(b)** The mean Kendall correlation coefficients between time labels and pseudotime for three types of synthetic temporal scRNA-seq data: sequential cell sampling, uniform cell sampling and mixed cell sampling models. **(c-d)** The heatmap shows the sampling probabilities for two types of high asynchronized cell differentiation models. In the random cell sampling, the time labels were uniformly sampled. In the mixed cell sampling, the sampling process was independent of time points. **(e)** The simulated temporal scRNA-seq datasets with five lineages ($N = 2{,}000$ genes and $M = 500$ cells, with an exponential decay model for the dropout noise), with the color indicating the cell lineages. **(f-k)** The distribution of simulated time index, pseudotime index inferred by the MDS method, and the TCM visualization of single cells for **(f-h)** uniform cell sampling and **(i-k)** mixed cell sampling. Note that for both differentiation models, the pseudotime labels reflected developmental speed better than the original time labels. **(l-m)** The visualization of simulated scRNA-seq datasets using t-SNE and diffusion map. **(n-o)** For random and highly asynchronized temporal scRNA-seq data (random and mixed cell sampling), the use of pseudotime labels had significantly better performance for the discovery of lineages and the separation of cell lineages from the last pseudotime points in our simulation studies. In boxplot, the upper whisker is located at the smaller of the maximum input value and Q_3+1.5*IQR, and the lower whisker is located at the larger of the smallest input value and Q_1-1.5*IQR, where Q_1 and Q_3 are the first and third quantile of the input data, and IQR = Q_3 - Q_1, the box length.

Supplementary Figure 22

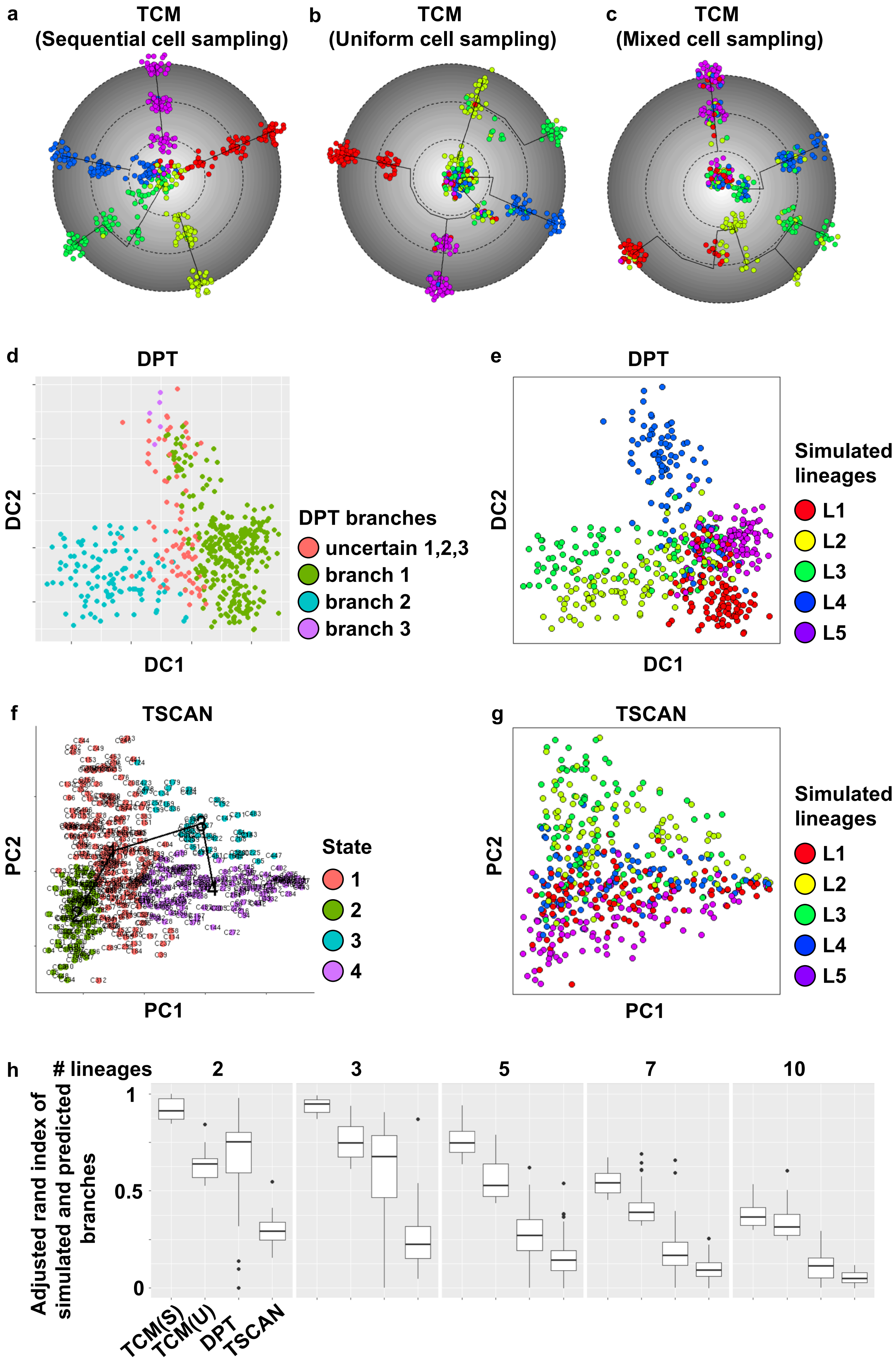

Supplementary Figure 22

i

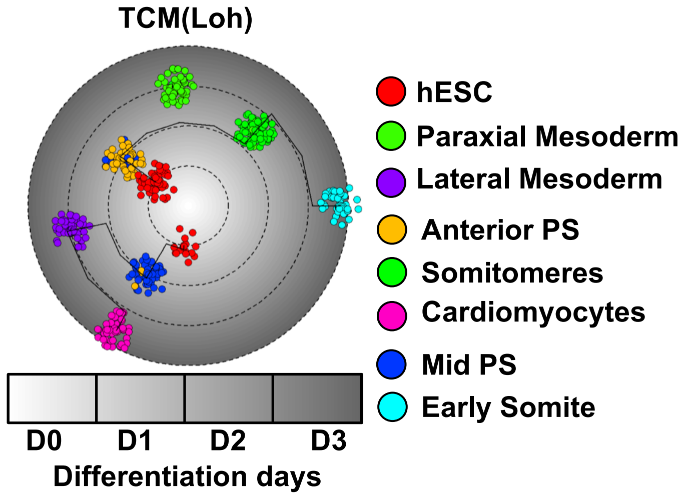

Supplementary Figure 22. Inferring the developmental trajectories from TCM representations. (a-c) TCM recovered the simulated lineages under three cell differentiation models (sequential, uniform and mixed cell sampling) ($N = 2{,}000, M = 500$). The pseudotime labels were used for the uniform and mixed cell sampling. **(d-g)** The trajectory inferences of simulated scRNA-seq datasets using diffusion pseudotime (DPT) and TSCAN. Note that both DPT and TSCAN were unable to separate closely related lineages (e.g. simulated lineages L2 and L3 for DPT). **(h)** The performance of trajectory inference between TCM (both sequential cell sampling using time labels and uniform cell sampling using pseudotime labels), DPT and TSCAN, under 2, 3, 5, 7, and 10 simulated lineages ($N = 2{,}000, M = 500$). The performance was measured by the adjusted Rand Index between simulated and predicted lineages. TCM(S): TCM on sequential cell sampling; TCM(U): TCM on uniform cell sampling. **(i)** The trajectory inference using the Loh et al. dataset on hESC differentiation after merging the D2.25 somitomere cells with D2 cells. The inferred trajectory generally reflected the bifurcation of paraxial and lateral mesodermal lineages. In boxplot, the upper whisker is located at the smaller of the maximum input value and Q_3+1.5*IQR, and the lower whisker is located at the larger of the smallest input value and Q_1-1.5*IQR, where Q_1 and Q_3 are the first and third quantile of the input data, and IQR = Q_3 - Q_1, the box length.

Supplementary Figure 23

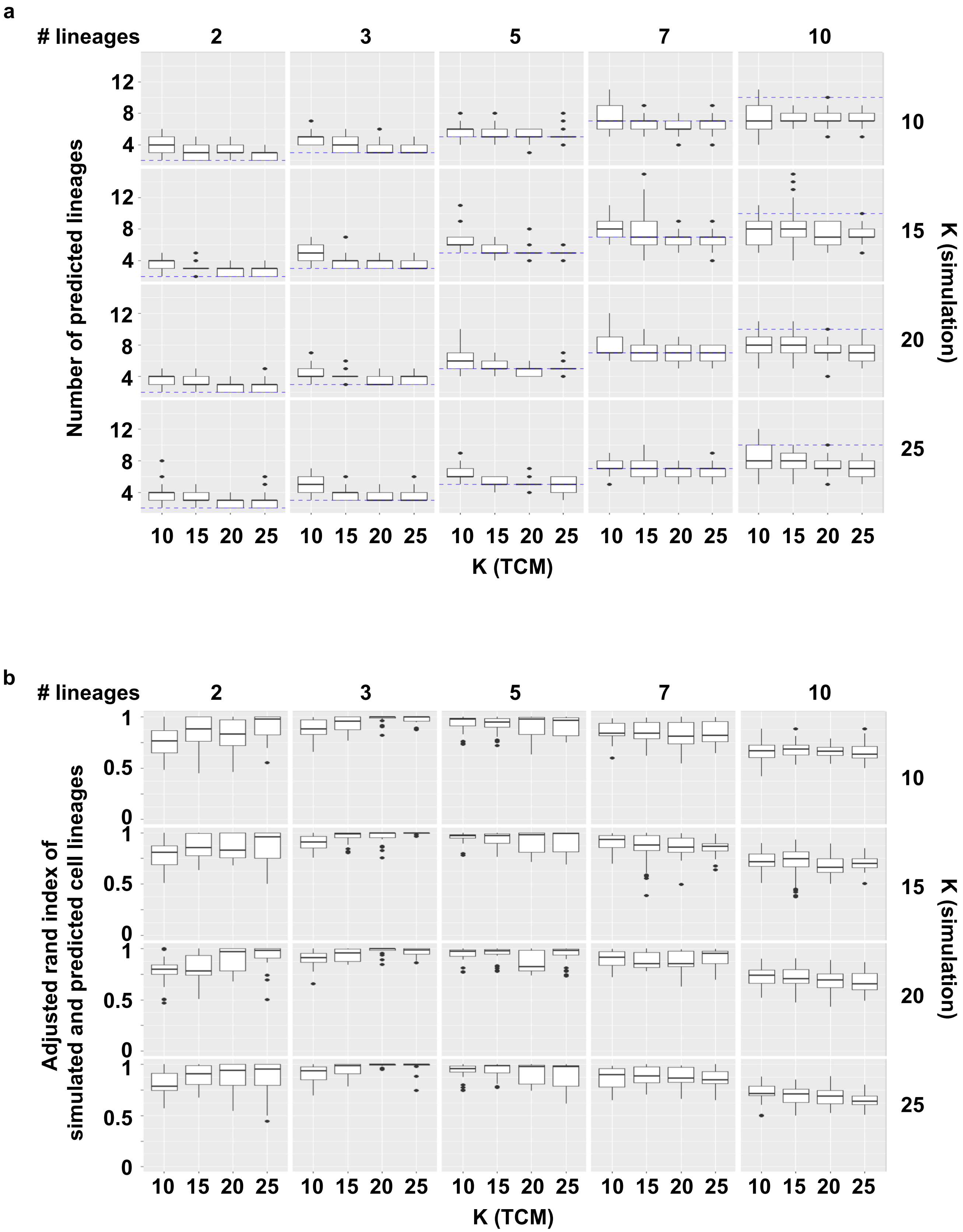

Supplementary Figure 23. The performance of TCM was robust using different choices of K's. We evaluated the performance of TCM with multiple K's ($K = 10,15,20,25$) on simulated temporal scRNA-seq data with multiple K's ($K = 10,15,20,25$). The performance was measured by **(a)** the number of estimated lineages from the last time point and **(b)** the adjusted Rand index between the estimated cell lineages and simulated cell lineages. In boxplot, the upper whisker is located at the smaller of the maximum input value and Q_3+1.5*IQR, and the lower whisker is located at the larger of the smallest input value and Q_1-1.5*IQR, where Q_1 and Q_3 are the first and third quantile of the input data, and IQR = Q_3 - Q_1, the box length.

Supplementary Figure 24

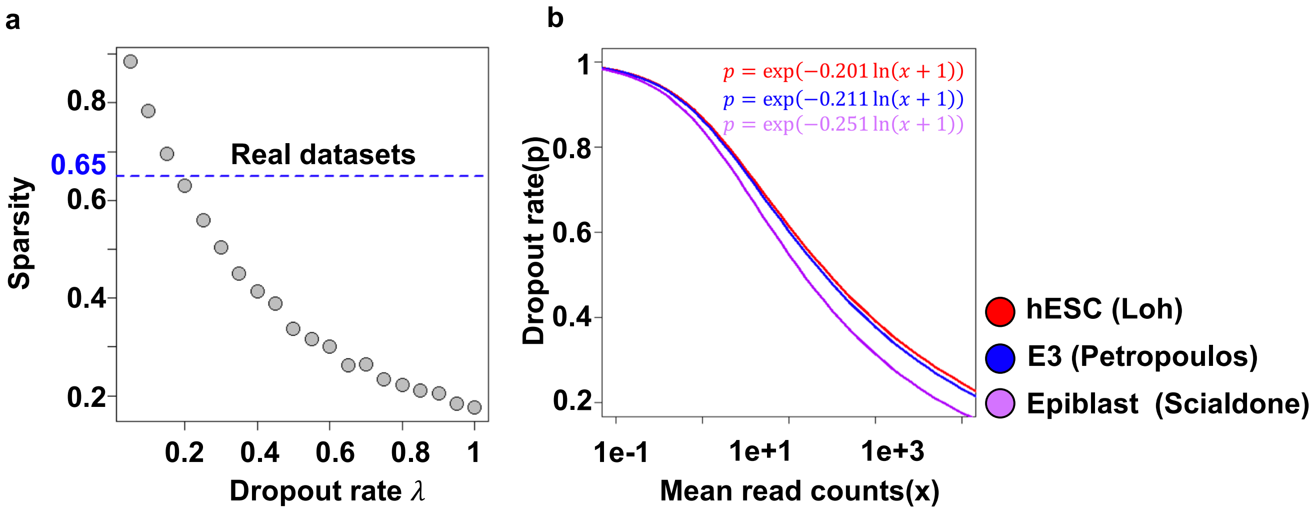

Supplementary Figure 24. The choice of dropout rate for simulating scRNA-seq data. (a) The sparsity (the ratio of zero entries over all entries) of simulated scRNA-seq data ($N = 20{,}000$, library size between 10^3 and 10^6) was most similar to the sparsity observed in nine real scRNA datasets when $\lambda = 0.25$. **(b)** We inferred the λ values from three relatively homogenous scRNA-seq subsets among all the scRNA-seq datasets examined in these studies (hESC, developing human embryo at E3 and mouse D6.5 epiblast cells), which did not have very distinct subpopulations of cells. For each dataset, we selected the genes that were detectable in at least one cell (raw read count >1), and for each gene n, calculated the observed probability of dropout p_n (number of non-detectable cells divided by the total cells), as well as the mean read counts in detectable cells x_n, followed by the estimation of λ by minimizing a cross-entropy loss between observed and predicted dropout rate using R's one dimensional optimization function *optimize()*: $-\sum_{n=1}^{N}\left[p_n(-\lambda \ln x_n) + (1 - p_n)\ln\left(1 - \exp(-\lambda \ln x_n)\right)\right]$. The inferred λ values were 0.201, 0.211 and 0.251, respectively.

Supplementary Note 1

Simulation of temporal scRNA-seq datasets

In a single cell RNA-seq dataset with N genes and M cells, let $\hat{x}_{nm}$ be *expected* number of reads of the gene n in the cell m, where $n = 1, \cdots, N$ and $m = 1, \cdots, M$. We randomly sampled the expected read counts $\hat{x}_{nm}$ from a multinomial distribution where the total counts (library size of cell m) is ls_m and the probability that gene m being drawn is p_{nm} ($\sum_{i=1}^{N} p_{im} = 1$).

$$(\hat{x}_{1m}, \cdots, \hat{x}_{nm}, \cdots, \hat{x}_{Nm}) \sim \text{Multinom}(ls_m, p_{1m}, \cdots, p_{nm}, \cdots, p_{Nm}) \quad (1)$$

$$ls_m \sim \text{Uniform}(10^3, 10^5) \quad (2)$$

where the library size was randomly sampled from a uniform distribution between 10^3 and 10^5 total reads. The probability p_{nm} was defined as the product of two parts:

$$p_{nm} = \sum_{k=1}^{K} u_{nk} \sigma(v_{km}) \quad (3)$$

where K is the number of metagenes (see Online Methods for the definitions of metagenes), and σ is the softmax function. The cell independent metagene basis, u_{nk}, represents the expression probability of gene x in the k-th metagene, and we randomly sampled it from a Dirichlet distribution:

$$u_{1:N,k} \sim \text{Dir}(\alpha_0) \quad (4)$$

The metagene coefficient, v_{km}, is a real variable, indicating the contribution of the k-th metagene for cell m. We sampled $\boldsymbol{v}_m$ from a defined latent space $\boldsymbol{\Phi}$ with H prototypes where each prototype is represented by a unique metagene coefficient $\boldsymbol{\phi}_h (h = 1, \cdots, H)$.

The 2D coordinate of the h-th prototype on latent space is represented as $\boldsymbol{y}_h = (l_r cos\omega_s, l_r sin\omega_s)$, assuming that the prototype locates at the r-th layer with the polar angle ω_s, where $r \in [1, \cdots, R]$, $s \in [1, \cdots, S]$, $\omega_s = \frac{s}{S} 2\pi$ and $l_r = \frac{r}{R}$. Thus, the

total number of prototypes on the latent space is $H = SR$. We defined the covariance matrix between each pairs of prototypes as:

$$B_{ij} = \exp\left(-\frac{\left\|\boldsymbol{y}_i - \boldsymbol{y}_j\right\|_2^2}{2s_0}\right) \tag{5}$$

where $i, j \in [1, \cdots, H]$. Then, the metagene coefficients of each prototype were randomly sampled from a centered multivariate normal distribution:

$$\boldsymbol{\theta}_{k,1:H} \sim \mathcal{N}\left(\boldsymbol{\theta}_{k,1:H} | \mathbf{0}, \mathbf{B}\right) \tag{6}$$

Case 1: homogenous scRNA-seq data

For simulating homogenous scRNA-seq data (e.g. when investigating the performance of TCM using a homogenous scRNA-seq dataset with arbitrary time index), we simply randomly select any prototype h, and let $\boldsymbol{v}_m = \boldsymbol{\theta}_h$.

Case 2: temporal scRNA-seq dataset with one (linear differentiation) or multiple lineages

For simulating temporal scRNA-seq datasets with L lineages, we first randomly selected L prototypes from the outer layer (the R-th layer) as the terminal prototype for each simulated lineage, and constructed the shortest pathways from the origin to each terminal prototype. The metagene coefficient of cell m was randomly sampled as one of the prototypes on the L shortest paths. The scRNA-seq data with linear differentiation can be viewed as a temporal scRNA-seq data with one lineage. It should be noted that since each cell m was sampled from a specific prototype h, the layer where the prototype h was located indicated the differentiation status of the prototype h, and therefore the cell m. For example, the cells sampled from the peripheral area of the latent space had more diversified metagene coefficients, due to the covariance matrix $\mathbf{B}$ we defined to constraint the metagene coefficients of each prototype on the latent space. We defined $g_m \in [1, \cdots, R]$ as the layer index associated with each sampled cell and applied them to construct different cell differentiation models.

Thus, the layer index g_m can be also viewed as the developmental speed for the cell m.

The dropout noise

Once $\hat{x}_{nm}$ was sampled as the expected read counts of the gene n in the cell m, we further applied an exponential decay function to determine the probability of the dropout of the gene n in the cell m[1,2], q_{nm}, that is,

$$q_{nm} = \exp(-\lambda \ln \hat{x}_{nm}) \tag{7}$$

$$\xi_{nm} = \mathrm{Bern}(q_{nm}) \tag{8}$$

$$x_{nm} = \begin{cases} \hat{x}_{nm} & \xi_{nm} = 0 \\ 0 & \xi_{nm} = 1 \end{cases} \tag{9}$$

where ξ_{nm} represents whether or not the dropout event occurs for gene n in the cell m, and x_{nm} is the *observed* read counts of the gene n in the cell m used for the following analysis.

In our simulations, we used $K = 15$, $R = 100$, $S = 15$, $\lambda = 0.25$ and $\alpha_0 = 0.1$. We found the performance of TCM was robust with different choices of K's used in simulation (Supplementary Figure 23), and $\lambda = 0.25$ gave the realistic sparsity and appropriate fitting of real homogenous scRNA-seq data (Supplementary Figure 24). Additionally, we have implemented the simulation process as a standalone function *simulate.rnaseq.ts* in our TCM R package.

Supplementary Note 2

Differentiation models and assignment of time index to simulated temporal scRNA-seq dataset

For simulated temporal scRNA-seq datasets with $\boldsymbol{T}$ time points (see Appendix R1 for the details of simulating temporal scRNA-seq datasets), the differentiation models defined how cells were sampled at each time point. To systematically characterize the performance using TCM on complex developmental processes, we investigated three different temporal cell differentiation models: (1) sequential cell sampling, (2) delayed cell sampling and (3) forward cell sampling, and two random cell differentiation models: (4) uniform sampling and (5) mixed sampling.

Let p_{rt} be the probability of assigning the time index t to a cell from the r-th layer ($\sum_{i=1}^{R} p_{it} = 1$). We defined the time index when the cell was sampled as $\tau_m \in [1, \cdots, T]$, and the developmental speed for cell m as $g_m \in [1, \cdots, R]$. Thus, assigning time index to single cells can be viewed as the following sampling process:

$$\tau_m \sim \mathrm{Cat}\left(T, \frac{1}{T}\right) \tag{1}$$

$$g_m \sim \mathrm{Cat}\left(R, p_{1,\tau_m}, \cdots, p_{r,\tau_m}, \cdots, p_{R,\tau_m}\right) \tag{2}$$

where Cat represents the Categorical distribution.

Among three temporal cell differentiation models, in the *sequential cell sampling*, the sampling time τ_m is positively correlated to the developmental speed g_m (Supplementary Fig. 2a, left panel). In the *delayed cell sampling*, the more primitive (progenitor) cells with smaller g_m could be sampled at a later time point (Supplementary Fig. 2a, mid panel), and while in the *forward cell sampling*, the more differentiated cells with larger g_m could be sampled at an earlier time point (Supplementary Fig. 2a, right panel).

Among two random cell differentiation models, in the uniform cell sampling, the probability of assigning the time index t to a cell from the r-th layer is a constant $\frac{1}{R}$ (i.e. $p_{rt} = \frac{1}{R}$, Supplementary Figure 21c), while in the mixed cell sampling, the developmental speed distribution $p_{r,1:T}$ is independent of the time points (Supplementary Figure 21d).

Additionally, we have implemented five differentiation models as the argument *types* (*sequential*, *delayed*, *forward*, *uniform* and *mixed*) in the standalone function *sim.rnaseq.ts* in our TCM R package.

Supplementary Note 3

The performance of TCM on simulated temporal scRNA-seq datasets

We examined the performance of TCM on four types of synthetic temporal scRNA-seq datasets: (1) asynchronized temporal scRNA-seq datasets, (2) A homogenous scRNA-seq dataset with arbitrary time index, (3) A temporal scRNA-seq with a single lineage, and (4) A temporal scRNA-seq dataset with a mixture of temporally varying and non-varying cells.

Case 1: Asynchronized temporal scRNA-seq datasets

The real temporal scRNA-seq data usually include the cells from asynchronized developmental or differentiation process, that is, cells from any time point may come from multiple lineages with heterogeneous developmental speed.

To systematically characterize the performance of TCM on such complex developmental processes, we simulated temporal scRNA-seq datasets with three different cell differentiation models: (1) sequential cell sampling, (2) delayed cell sampling and (3) forward cell sampling (see Supplementary Note 1 for the details of simulating the temporal scRNA-seq dataset and Supplementary Note 2 for three cell differentiation models) (Supplementary Fig. 2a). Before applying the differentiation model, we first simulated synchronized differentiating cell populations with a certain number of lineages in a ring-shaped latent space with $\boldsymbol{R}$ layers, where these cells resided from the inner (more progenitor states) to the peripheral area (more differentiated states). For temporal scRNA-seq datasets with $\boldsymbol{T}$ time points, the differentiation models defined how these simulated cells were sampled at each time point. In the *sequential cell sampling*, the sampling time is positively correlated to the developmental speed. In the *delayed cell*

sampling, the more primitive (progenitor) cells could be sampled at a later time point, while in the *forward cell sampling*, the more differentiated cells could be sampled at an earlier time point[3,4].

Supplementary Fig. 2b showed the simulated temporal scRNA-seq datasets with five lineages under three differentiation models ($N = 2{,}000$ genes and $M = 500$ cells, with an exponential decay model for the dropout noise). As expected, in the sequential cell sampling, the sampled cells were relatively uniformly distributed across the ring (latent space), while in delayed and forward cell sampling models, more progenitor cells (located in the inner area) and differentiated cells (located in the peripheral area) were sampled, respectively (Supplementary Fig. 2c).

We found that TCM was able to successfully reveal the lineage trajectories for all three differentiation models. However, the performance was visually better in sequential and forward cell sampling models, as in these two models there existed a relatively higher number of more differentiated cell populations with relatively stronger expression distinctions. TCM relied on the global expression distinctions of different lineages across neighboring time points to map the cells onto the latent space. Similarly, we found that it was also easier for t-SNE and diffusion map to reveal distinct lineages for sequential and forward cells sampling models than delayed cell sampling model (Supplementary Fig. 2e-2h).

We quantitatively compared the performance for the determination of the number of clusters of cells from the last time points between TCM and gap statistics[5,6] on simulated datasets (20 random seeds) with different number of lineages ($\boldsymbol{L} = 2,3,5,7,10$) under three different differentiation models ($N = 2{,}000$ genes, $M = 500$ cells and $T = 5$ time points, with an exponential decay model for the dropout noise). We found that TCM has an overall better performance for the determination of the correct number of lineages existing in the cell populations. Finally, we quantitatively compared the performance for the clustering of cells

from the last time points using adjusted Rand index between the true cell lineage labels and the cell cluster labels determined by TCM, t-SNE followed by k-means, and diffusion map followed by *k*-means. For both t-SNE and diffusion map, the number of clusters (the argument required for *k*-means clustering) was determined by gap-statistics. The results suggested TCM has overall better performance than t-SNE and diffusion map followed *k*-means for correctly separating the cells from different lineages (Supplementary Fig. 2j).

Collectively, these simulation studies suggested that TCM was able to reveal the lineage trajectories under three differentiation models. TCM has the best performance for the determination of the number of cell clusters and the separation of the cells from different lineages on all three differentiation models compared to other tested methods, such as gap-statistics, t-SNE and diffusion map.

Case 2: A homogenous scRNA-seq dataset with arbitrary time index

We simulated a homogenous scRNA-seq dataset with $N = 2{,}000$ genes and $M = 500$ cells, having an exponential decay model for the dropout noise (see Supplementary Note 1 for the details of simulating the temporal scRNA-seq dataset and Supplementary Note 2 for three cell differentiation models)[1]. Cells were randomly split into $T = 5{,}10{,}15{,}20$ time points. As expected, the visualization of the homogenous scRNA-seq data using t-SNE did not identify any distinct subpopulations (Supplementary Fig. 3a). In contrast, TCM tended to group the cells into a single trajectory from the central to the peripheral area, as these cells, though randomly assigned to different time points, all had a similar expression pattern (Supplementary Fig. 3b). We repeated the above simulations 20 times, and measured, among the cells from the last time point, the proportions of cells that were assigned to the largest cluster (Supplementary Fig. 3c) and determined the number of cell clusters that were generated (Supplementary Fig. 3d). These results suggested that TCM tended to generate a single trajectory

using a simulated homogenous scRNA-seq data with random arbitrary time index and was unlikely to generate false positive branches. We also investigated the performance of TCM using a scRNA-seq dataset of a relatively homogenous hematopoietic stem cell (HSC) population with a random arbitrary time index[7]. In a similar fashion (compared to the simulation study), we observed that t-SNE did not produce any distinct subpopulations of HSCs (Supplementary Fig. 3e), while TCM generated single trajectories for HSCs with arbitrary time index (Supplementary Fig. 3f). Taken together, these results supported the notion that TCM was likely to generate a single trajectory of homogenous scRNA-seq dataset with randomly assigned time index and did not produce false positive lineage and branches.

Case 3. A temporal scRNA-seq dataset with a single lineage

First, to investigate the performance of TCM using the temporal scRNA-seq dataset without any branching events, we simulated scRNA-seq datasets ($N = 2{,}000$ genes and $M = 500$ cells, with an exponential decay model for the dropout noise) of linear differentiation, and randomly sampled the cells at $T = 5{,}10{,}15{,}20$ time points (see Supplementary Note 1 for the details of simulating the temporal scRNA-seq dataset and Supplementary Note 2 for three cell differentiation models). As expected, t-SNE and diffusion map successfully captured dominant temporal expression differences and produced a linear trajectory of cells (Supplementary Fig. 3g and 3h). TCM was also likely to produce a single trajectory of cells. When the number of time points increased, TCM produced a straight line-like projection of cells on the latent space (Supplementary Fig. 3i-3l). We repeated the above simulations 20 times, and measured, among the cells from the last time point, the proportion of cells that were assigned to the largest cluster (Supplementary Fig. 3m) and determined the number of cell clusters that were generated (Supplementary Fig. 3n). In summary, we determined that using the TCM algorithm on a simulated temporal scRNA-seq dataset with linear

differentiation predominantly produced a single trajectory of cells, and was unlikely to produce false positive branches.

Case 4. A temporal scRNA-seq dataset having a mixture of temporally varying and non-varying cells

By using the scRNA-seq simulation methods described in Supplementary Note 1, we simulated 250 temporally varying cells with linear differentiation and 250 homogenous temporally non-varying cells ($N = 2{,}000$ genes and $T = 5$ time points). The temporally non-varying cells were randomly split into five time points. As expected, t-SNE and diffusion map grouped the cells into two distinct populations, and diffusion map was able to reveal the differentiation trajectory among the simulated temporally varying cells (Supplementary Fig. 3o and 3p). Similarly, TCM also produced two distinct major trajectories for two subpopulations (Supplementary Fig. 3q). As our previous analysis of Case 2 (a homogenous scRNA-seq dataset with arbitrary time index) and Case 3 (a temporal scRNA-seq with one single lineage) suggested, TCM is likely to produce a linear trajectory for both temporally varying and non-varying scRNA-seq data. When mixing these two distinct types temporal scRNA-seq data together, TCM produced two separate trajectories for each subpopulations.

Supplementary Note 4

A brief introduction to the mean-field variance inference

We consider a generic model with observations $\mathbf{X}$ and all hidden variables $\mathbf{Z}$,

$$p(\mathbf{X},\mathbf{Z}) = p(\mathbf{X}|\mathbf{Z})p(\mathbf{Z}) \tag{1}$$

The inference problem is to compute the posterior,

$$p(\mathbf{Z}|\mathbf{X}) = \frac{p(\mathbf{X},\mathbf{Z})}{\int p(\mathbf{X},\mathbf{Z})d\mathbf{Z}} \tag{2}$$

This posterior for many models is intractable because the denominator is usually difficult to compute in a closed form. In the mean-field variational inference, the posterior is approximated by positing a fully factorized variational family of distributions over latent variables sets $\mathbf{Z}_i$**,** where $i = 1,\cdots,M$, so that:

$$q(\mathbf{Z}) = \prod_{i=1}^{M} q_i(\mathbf{Z}_i) \tag{3}$$

In this family of distributions, the variables are independent and each is governed by its own distribution. In the standard variational theory, minimizing the KL divergence between $q(\mathbf{Z})$ and the posterior $p(\mathbf{Z}|\mathbf{X})$ is equivalent to maximizing a lower bound of the log marginal likelihood of the observed data $\mathbf{X}$. This lower bound can be observed by using Jensen's inequality:

$$\ln p(\mathbf{X}) = \int p(\mathbf{X},\mathbf{Z})d\mathbf{Z} \geq E_q[p(\mathbf{X},\mathbf{Z})] - E_q[q(\mathbf{Z})] \triangleq \mathcal{L}(q) \tag{4}$$

where E_q is the expectation taken with respect to the variational distribution q and the second term is the entropy of q. The term $\mathcal{L}(q)$ is defined as the variational objective.

Setting $\partial\mathcal{L}(q)/\partial q = 0$ shows that the optimal solution satisfies the following,

$$q_i^*(\mathbf{Z}_i) \propto \exp\{E_{i\neq j}[p(\mathbf{Z})]\} \tag{5}$$

where $E_{i\neq j}[\cdots]$ denotes an expectation with respect to the q distributions over all variables $\mathbf{Z}_i$ for $i \neq j$. These conditions led to the coordinate ascent algorithm for

variational inference. This converges to a local optimum of the variational objective[8].

Inference and parameter estimation of TCM

The key inferential problem we need to solve in order to use TCM is that of computing the posterior distribution of the hidden variables given $\mathbf{X}$. Unfortunately, this distribution is intractable to compute in general. We adopted variational and Laplace approximation algorithms for inference in TCM[8-10]. The variational inference is to make use of Jensen's inequality to obtain an adjustable lower bound on the log likelihood and the variational parameters are chosen by an optimization procedure that attempts to find the tightest lower bound.

The variational objective function for TCM is given below:

$$\mathcal{L} = E_q[\ln p(\mathbf{X}|\mathbf{S})] + E_q[\ln p(\mathbf{S}|\mathbf{U},\mathbf{V})] + E_q[\ln p(\mathbf{U})] + E_q[\ln p(\mathbf{V}|\boldsymbol{\Theta},\mathbf{Z},\beta)] + E_q[\ln p(\mathbf{Z}|\boldsymbol{\pi})] + E_q[\ln p(\boldsymbol{\pi})] + \ln p(\boldsymbol{\Theta}) + H[Q] \quad (6)$$

and $E_q[\cdot]$ represents the expected values with respect to the variational distributions, and the log probability distributions of TCM variables are:

$$\ln p(\mathbf{X}|\mathbf{S}) = \sum_{t=1}^{T}\sum_{n=1}^{N}\sum_{m^{(t)}=1}^{M^{(t)}} \ln \delta\left(x_{n,m^{(t)}} - \sum_{k=1}^{K} s_{n,k,m^{(t)}}\right) \quad (7)$$

$$\ln p(\mathbf{S}|\mathbf{U},\mathbf{V}) = \sum_{t=1}^{T}\sum_{n=1}^{N}\sum_{k=1}^{K}\sum_{m^{(t)}=1}^{M^{(t)}} \ln \mathrm{Pois}\left(s_{n,k,m^{(t)}}|u_{nk}\exp\left(a_{m^{(t)}} + v_{k,m^{(t)}}\right)\right) \quad (8)$$

$$\ln p(\mathbf{U}) = \sum_{n=1}^{N}\sum_{k}^{K} \mathrm{Gamma}(u_{nk}|c_0,d_0) \quad (9)$$

$$\ln p(\mathbf{V}|\boldsymbol{\Theta},\mathbf{Z},\beta) = \sum_{t=1}^{T}\sum_{m^{(t)}=1}^{M^{(t)}}\sum_{h=1}^{H} z_{m^{(t)},h} \ln \mathcal{N}\left(\boldsymbol{v}_{m^{(t)}}|\boldsymbol{\Theta}\boldsymbol{g}_h^{(t)},(\beta\mathbf{I})^{-1}\right) \quad (10)$$

$$\ln p(\boldsymbol{\Theta}) = -\frac{1}{2}\mathrm{tr}(\boldsymbol{\Theta}\mathbf{B}^{-1}\boldsymbol{\Theta}^{\mathrm{T}}) - \frac{DK}{2}\ln(2\pi) - \frac{D}{2}\ln|\mathbf{B}| \quad (11)$$

$$\ln p(\mathbf{Z}|\boldsymbol{\pi}) = \sum_{t=1}^{T} \sum_{m^{(t)}=1}^{M^{(t)}} \sum_{h=1}^{H} z_{m^{(t)},h} \ln \pi_h^{(t)} \tag{12}$$

$$\ln p(\boldsymbol{\pi}) = \sum_{t=1}^{T} \ln \mathrm{Dir}\left(\boldsymbol{\pi}^{(t)} | \alpha_0\right) \tag{13}$$

The equations (7) - (13) correspond to the equations (1) - (9) in the Online Methods section.

Parameter update for $q(\boldsymbol{S})$

$q(\mathbf{S})$

$$= \prod_{t=1}^{T} \prod_{n=1}^{N} \prod_{m^{(t)}=1}^{M^{(t)}} \exp\left(E_q \left[\sum_{k=1}^{K} \left(s_{n,k,m^{(t)}} \left(\ln u_{nk} + v_{k,m^{(t)}} \right) - \Gamma\left(s_{n,k,m^{(t)}} + 1 \right) \right) \right.\right.$$

$$\left.\left. + \ln \delta \left(\sum_{k=1}^{K} s_{n,k,m^{(t)}} - x_{n,m^{(t)}} \right) \right] \right)$$

$$= \prod_{t=1}^{T} \prod_{n=1}^{N} \prod_{m^{(t)}=1}^{M^{(t)}} \mathrm{Multinom}\left(s_{n,1:K,m^{(t)}};\ x_{n,m^{(t)}}, \rho_{n,1:K,m^{(t)}} \right) \tag{14}$$

where

$$\rho_{n,k,m^{(t)}} = \frac{\exp\left(E_q[\ln u_{nk}] + E_q\left[v_{k,m^{(t)}} \right] \right)}{\sum_{j=1}^{K} \exp\left(E_q\left[\ln u_{nj} \right] + E_q\left[v_{j,m^{(t)}} \right] \right)} \tag{15}$$

Thus,

$$E_q\left[s_{n,1:K,m^{(t)}} \right] = x_{n,m^{(t)}}\, \rho_{n,1:K,m^{(t)}} \tag{16}$$

Parameter update for metagene basis $q(\boldsymbol{U})$

$q(\boldsymbol{U})$

$$\propto \prod_{n=1}^{N} \prod_{k=1}^{K} \exp\left(E_q \left[\sum_{t=1}^{T} \sum_{m^{(t)}=1}^{M^{(t)}} s_{n,1:K,m^{(t)}} \ln u_{nk} - \sum_{t=1}^{T} \sum_{m^{(t)}=1}^{M^{(t)}} u_{nk} \exp\left(a_{m^{(t)}} + v_{k,m^{(t)}} \right) \right.\right.$$

$$\left.\left. + (c_0 - 1) \ln u_{nk} - d_0 u_{nk} \right] \right)$$

$$= \prod_{n=1}^{N} \prod_{k=1}^{K} \text{Gamma}(u_{nk} | c_{nk}, d_{nk}) \tag{17}$$

where

$$c_{nk} = \sum_{t=1}^{T} \sum_{m^{(t)}=1}^{M^{(t)}} E_q\left[s_{n,1:K,m^{(t)}}\right] + c_0 \tag{18}$$

$$d_{nk} = \sum_{t=1}^{T} \sum_{m=1}^{M} \exp\left(a_{m^{(t)}}\right) E_q\left[\exp\left(v_{k,m^{(t)}}\right)\right] + d_0 \tag{19}$$

Thus,

$$E_q[u_{nk}] = \frac{c_{nk}}{d_{nk}} \tag{20}$$

$$E_q[\ln u_{nk}] = \psi(c_{nk}) - \ln d_{nk} \tag{21}$$

where $\psi(a) = \frac{d}{da} \ln \Gamma(a)$ is the *digamma* function and $\Gamma(a)$ is the *gamma* function.

Parameter update for metagene coefficients $q(\boldsymbol{V})$

Since $\boldsymbol{V}$ is conditionally non-conjugate, we used Laplace variance inference to approximate the variational distribution of $\mathbf{V}$. Laplace approximation use a Taylor approximation around the maximum a posterior (MAP) point to construct a Gaussian proxy for the posterior.

$$q(\boldsymbol{V})$$

$$\propto \prod_{t=1}^{T} \prod_{m^{(t)}=1}^{M^{(t)}} \exp\left(\sum_{n=1}^{N} \sum_{k=1}^{K} \left(E_q\left[s_{n,k,m^{(t)}}\right] v_{km} - E_q[u_{nk}] \exp\left(a_{m^{(t)}} + v_{km}\right)\right)\right.$$

$$\left. + \sum_{h=1}^{H} E_q\left[z_{m^{(t)},h}\right] \left(-\frac{1}{2}\left(\left(\boldsymbol{v}_{m^{(t)}}\right)^T \boldsymbol{v}_{m^{(t)}} - 2\left(\boldsymbol{v}_{m^{(t)}}\right)^T \boldsymbol{\Theta} \boldsymbol{g}_h^{(t)}\right)\right)\right)$$

$$= \prod_{t=1}^{T} \prod_{m^{(t)}=1}^{M^{(t)}} \mathcal{N}\left(\widehat{\boldsymbol{\vartheta}}_{m^{(t)}}, -\nabla^2 f\left(\widehat{\boldsymbol{\vartheta}}_{m^{(t)}}\right)^{-1}\right) \tag{22}$$

where

$$f\left(\boldsymbol{v}_{m^{(t)}}\right) = \mathcal{L}_{\left[\boldsymbol{v}_{m^{(t)}}\right]}$$

$$= \sum_{n=1}^{N} \sum_{k=1}^{K} \left(E_q\left[s_{n,k,m^{(t)}}\right] v_{k,m^{(t)}} - E_q[u_{nk}] \exp\left(a_{m^{(t)}} + v_{k,m^{(t)}}\right)\right)$$

$$-\frac{1}{2}\sum_{h=1}^{H} E_q\left[z_{m^{(t)},h}\right] \left(\left(\boldsymbol{v}_{m^{(t)}}\right)^T \boldsymbol{v}_{m^{(t)}} - 2\left(\boldsymbol{v}_{m^{(t)}}\right)^T \boldsymbol{\Theta} \boldsymbol{g}_h^{(t)}\right) \quad (23)$$

$\widehat{\boldsymbol{\vartheta}}_{m^{(t)}}$ is the value that maximize $f\left(\boldsymbol{v}_{m^{(t)}}\right)$ and $\nabla^2 f\left(\widehat{\boldsymbol{\vartheta}}_{m^{(t)}}\right)$ is the Hessian matrix evaluated at $\widehat{\boldsymbol{\vartheta}}_{m^{(t)}}$. Note that we did not assume $\boldsymbol{v}_{m^{(t)}}$ is Gaussian. The Gaussian form in equation (21) comes from the Taylor approximation. Thus,

$$E_q\left[\boldsymbol{v}_{m^{(t)}}\right] \approx \widehat{\boldsymbol{\vartheta}}_{m^{(t)}} \quad (24)$$

$$E_q\left[\exp\left(\boldsymbol{v}_{m^{(t)}}\right)\right] \approx \exp\left(\widehat{\boldsymbol{\vartheta}}_{m^{(t)}} + \frac{1}{2}\operatorname{diag}\left(-\nabla^2 f\left(\widehat{\boldsymbol{\vartheta}}_{m^{(t)}}\right)^{-1}\right)\right) \quad (25)$$

Parameter update for $q(Z)$

$q(\mathbf{Z})$

$$= \prod_{t=1}^{T} \prod_{m^{(t)}=1}^{M^{(t)}} \prod_{h=1}^{H} \exp\left(z_{m^{(t)},h} \left(-\frac{1}{2} E_q\left[\left(\boldsymbol{v}_{m^{(t)}} - \boldsymbol{\Theta}\boldsymbol{g}_h^{(t)}\right)^T \left(\boldsymbol{v}_{m^{(t)}} - \boldsymbol{\Theta}\boldsymbol{g}_h^{(t)}\right)\right] - \frac{1}{2} K \ln(2\pi)\right.\right.$$

$$\left.\left. + E_q\left[\ln \pi_h^{(t)}\right]\right)\right)$$

$$= \prod_{t=1}^{T} \prod_{m^{(t)}=1}^{M^{(t)}} \prod_{h=1}^{H} \left(\frac{\gamma_{m^{(t)},h}}{\sum_{i=1}^{H} \gamma_{m^{(t)},i}} \right)^{z_{m^{(t)},h}} \quad (26)$$

where

$$\ln \gamma_{m^{(t)},h} = -\frac{\beta}{2} E_q\left[\left(\boldsymbol{v}_{m^{(t)}} - \boldsymbol{\Theta}\boldsymbol{g}_h^{(t)}\right)^T \left(\boldsymbol{v}_{m^{(t)}} - \boldsymbol{\Theta}\boldsymbol{g}_h^{(t)}\right)\right] - \frac{K}{2}\ln(2\pi) + E_q\left[\ln \pi_h^{(t)}\right] \quad (27)$$

Thus,

$$E_q\left[z_{m^{(t)},h}\right] = \gamma_{m^{(t)},h} \quad (28)$$

Parameter update for $q(\boldsymbol{\pi})$

$q(\boldsymbol{\pi})$

$$= \prod_{t=1}^{T} \exp\left(\sum_{h=1}^{H}\left(\sum_{m^{(t)}=1}^{M^{(t)}} E_q\left[z_{m^{(t)},h}\right] + \alpha_0 - 1\right)\ln \pi_h^{(t)}\right)$$

$$= \prod_{t=1}^{T} \mathrm{Dir}\left(\kappa_{1:H}^{(t)}\right) \tag{29}$$

where

$$\kappa_h^{(t)} = \sum_{m^{(t)}=1}^{M^{(t)}} E_q\left[z_{m^{(t)},h}\right] + \alpha_0 - 1 \tag{30}$$

Thus,

$$E_q\left[\ln \pi_h^{(t)}\right] = \psi\left(\kappa_h^{(t)}\right) - \psi\left(\sum_{i=1}^{H} \kappa_i^{(t)}\right) \tag{31}$$

Parameter update for $\boldsymbol{a}$

The variational objective function with respect to $a_{m^{(t)}}$is

$$\mathcal{L}_{\left[a_{m^{(t)}}\right]} = \sum_{n=1}^{N}\sum_{k=1}^{K}\left(E_q\left[s_{n,k,m^{(t)}}\right]a_{m^{(t)}} - E_q[u_{nk}]\exp\left(a_{m^{(t)}}\right)E_q\left[\exp\left(v_{k,m^{(t)}}\right)\right]\right) \tag{32}$$

Setting the derivative of $\mathcal{L}_{\left[a_{m^{(t)}}\right]}$ with respect to $a_{m^{(t)}}$ to zero, we can find the point estimate for $a_{m^{(t)}}$ in the following closed-form:

$$a_{m^{(t)}} = \ln \sum_{n=1}^{N}\sum_{k=1}^{K} E_q\left[s_{n,k,m^{(t)}}\right] - \ln \sum_{n=1}^{N}\sum_{k=1}^{K} E_q[u_{nk}]E_q\left[\exp\left(v_{k,m^{(t)}}\right)\right] \tag{33}$$

Parameter update for β

The variational objective function with respect to β is

$$\mathcal{L}_{[\beta]}$$

$$= \sum_{t=1}^{T}\sum_{m^{(t)}=1}^{M^{(t)}}\sum_{h=1}^{H} E_q\left[z_{m^{(t)},h}\right]\left(-\frac{\beta}{2}E_q\left[\left(\boldsymbol{v}_{m^{(t)}} - \boldsymbol{\Theta}\boldsymbol{g}_h^{(t)}\right)^T\left(\boldsymbol{v}_{m^{(t)}} - \boldsymbol{\Theta}\boldsymbol{g}_h^{(t)}\right)\right]\right.$$

$$\left. + \frac{1}{2}K\ln\beta\right) \tag{34}$$

Setting the derivative of $\mathcal{L}_{[\beta]}$ with respect to β to zero, we can find the point estimate for β in the following closed-form:

$$\beta = \sum_{t=1}^{T}\sum_{m^{(t)}=1}^{M^{(t)}}\sum_{h=1}^{H} TKH \Bigg/ \sum_{t=1}^{T}\sum_{m^{(t)}=1}^{M^{(t)}}\sum_{h=1}^{H} E_q\left[z_{m^{(t)},h}\right] E_q\left[\left(\boldsymbol{v}_{m^{(t)}} - \boldsymbol{\Theta}\boldsymbol{g}_h^{(t)}\right)^T\left(\boldsymbol{v}_{m^{(t)}} - \boldsymbol{\Theta}\boldsymbol{g}_h^{(t)}\right)\right] \quad (35)$$

***Parameter update for prototype metagene coefficient* $\boldsymbol{\Theta}$**

The variational objective function with respect to $\boldsymbol{\Theta}$ is

$$\mathcal{L}_{[\boldsymbol{\Theta}]} = \sum_{t=1}^{T}\sum_{m^{(t)}=1}^{M^{(t)}}\sum_{h=1}^{H} E_q\left[z_{m^{(t)},h}\right]\left(-\frac{\beta}{2}\left(-2E_q[\boldsymbol{v}_m]^T\boldsymbol{\Theta}\boldsymbol{g}_h^{(t)} + \left(\boldsymbol{g}_h^{(t)}\right)^T\boldsymbol{\Theta}^T\boldsymbol{\Theta}\boldsymbol{g}_h^{(t)}\right)\right)$$

$$+\sum_{t=1}^{T}\sum_{h=1}^{H_{free}}\sum_{k=1}^{K}\left(-\frac{1}{2}\boldsymbol{\Theta}\boldsymbol{g}_h^{(t)}\mathbf{B}^{-1}\left(\boldsymbol{\Theta}\boldsymbol{g}_h^{(t)}\right)^T\right)$$

$$= \mathrm{Tr}(\boldsymbol{\Theta}\mathbf{G}\mathbf{Z}^T\mathbf{V}^T) - \frac{1}{2}\beta\mathrm{Tr}(\boldsymbol{\Theta}\mathbf{G}\mathbf{R}\mathbf{G}^T\boldsymbol{\Theta}^T) - \frac{1}{2}\mathrm{Tr}(\boldsymbol{\Theta}\mathbf{G}\mathbf{B}^{-1}\mathbf{G}^T\boldsymbol{\Theta}^T) \quad (36)$$

where $\mathbf{R}$ is a diagonal matrix where

$$r_{ij} = \begin{cases} 0 & i \neq j \\ \sum_{m=1}^{M} E_q[z_{mh}] & i = j \end{cases} \quad (37)$$

The metagene coefficients for each prototype is updated by:

$$\boldsymbol{\Theta} = \mathbf{V}\mathbf{Z}\mathbf{G}^T(\beta\mathbf{G}\mathbf{R}\mathbf{G}^T + \mathbf{G}\mathbf{B}^{-1}\mathbf{G}^T)^{-\mathbf{1}} \quad (38)$$

The choices of prototypes per layer ($\boldsymbol{S}$), the number of layers of free prototypes ($\boldsymbol{\rho}$), and the number of layers ($\mathbf{R}$) for TCM

There are three major parameters for the ring-shape structure: (1) the number of prototypes per layer ($\mathbf{S}$), (2) the number of layers of free prototypes ($\boldsymbol{\rho}$), and (3) the number of layers ($\mathbf{R}$) (Supplementary Fig. 1b). We determined the optimal selection of these parameters using the simulated temporal scRNA-seq dataset with three differentiation models and five different lineages (2, 3, 5, 7 and 10)

(see Supplementary Note 1 for the details of simulating the temporal scRNA-seq dataset and Supplementary Note 2 for three cell differentiation models). For each dimension reduction result, we measured, (1) the adjusted Rand Index (ARI) between simulated and predicted cell lineages, and (2) the number of predicted lineages from the cells from the last time point. An optimal dimension reduction result would produce a high ARI between simulated and predicted cell lineages and the number of predicted cell lineages close to the simulated ones (the blue dashed lines). We found that 15 prototypes per layer ($S = 15$) (Supplementary Fig. 18a and 18b), three layers of convolving prototypes ($\mathrm{R} - \rho = 3$) (Supplementary Fig. 18c and 18d), and 10 layers per time point ($\mathrm{R} = 10$) (Supplementary Fig. 18e and 18f) achieved the overall best performance for both metrics. We used this parameter combination throughout the experiments.

Computational time for batch optimization

As we were interested in mapping the cells onto a 2D latent space instead of monitoring the convergence of the ELBO (evidence lower bound) during the optimization, we empirically monitored the change of cell assignment to the metacells ($\|\mathbf{\Delta Z}\|_F$). We found that this metric usually converges after 200 iterations, and the cell assignment to the metacells became stable (Supplementary Fig. 19a). Supplementary Fig. 19b shows that the computational time for running TCM on simulated datasets ($N = 10{,}000, S = 15, R - \rho = 3, R = 10$) after 200 iterations. As outlined, it took about 450 seconds for a dataset with 500 cells and about 6,500 seconds for a dataset with 10,000 cells. We have also implemented a fast stochastic variational inference (SVI) based optimization procedure for processing large scale scRNA-seq datasets (Supplementary Note 7).

Supplementary Note 5

TCM using scRNA-seq datasets without explicit time index

TCM was specifically designed to visualize the temporal scRNA-seq dataset where the time stamp or time index is available. When the time index is not available, TCM is reduced to a process of mapping single cells onto a single latent space, where all prototypes on such a latent space become free prototypes. Therefore, running TCM on scRNA-seq dataset without time index (i.e. named as non-temporal TCM or nt-TCM) can be viewed as a process of clustering single cells on the latent space.

We investigated the performance of nt-TCM on three independent scRNA-seq datasets without time index. First, using a previously published scRNA-seq dataset including 11 independent cell types[11], we demonstrated that nt-TCM was able to separate known cell populations (adjusted Rand Index = 0.843), though the performance was slightly worse than t-SNE (adjusted Rand index = 0.913) (Supplementary Fig. 20a and 20b). Interestingly, when applying nt-TCM on a relatively homogenous hematopoietic stem cell (HSC) population[12], nt-TCM was able to identify a separate sub-population of cells showing relatively high expression of Itga2b and low expression of Procr compared with the remaining cells (Supplementary Fig. 20c and 20d). This observation was consistent with results reported by Wilson et al.: which defined a HSC subpopulation with homogenous gene expression as the molecular overlapping population (MolO) and the remaining HSCs as cells with no molecular overlap (NoMO). In this study, Itga2b (EPCR) and Procr were the top ranking markers for MolO and NoMO, respectively. In contrast, the visualization of this HSC dataset using t-SNE did not suggest or define the separation of MolO and NoMO cell populations (Supplementary Fig. 20e). Finally, the application of nt-TCM on a scRNA-seq dataset of mESC with three different cell cycle stages (G1, S and G2M)[13], after the removal of 892 cell-cycle annotated genes, produced relatively

weaker separation of cells with different cell cycle stages compared to t-SNE (Supplementary Fig. 20f and 20g).

In summary, we found the use of TCM for scRNA-seq datasets without time index was able to separate the subpopulations on the latent space. When applying nt-TCM on relatively homogenous scRNA-seq dataset such as HSCs without time index, the individual cells tended to scatter across the whole latent space, where some subpopulations may show interesting biological meaning (e.g. NoMo HSCs). Applying nt-TCM on a sample of cycling cells (mESCs with three cell cycle stages) mixed the cells from different stages. More studies need to be conducted to compare the performance between nt-TCM and other conventional method such as t-SNE and diffusion map on non-temporal scRNA-seq datasets.

Supplementary Note 6

Correlation between the time labels and pseudotime on real temporal scRNA-seq datasets

Since TCM visualizes the temporal scRNA-seq data based on the known time labels, prior to the running of TCM, it is important to investigate the correlation between the known time labels and the underlying gene expression patterns. If the datasets have little or no correlation between the time labels and the underlying gene expression patterns, then the utilization of the known time labels directly may not provide any additional benefits for the visualization of the temporal scRNA-seq data.

We used the correlation coefficients (Kendall's tau) between the time labels and the pseudotime to investigate the relationship between known time labels and gene expression patterns, a strategy that has often been used to evaluate the performance of pseudotime inference algorithms[14,15]. We argued that a strong correlation between the known time stamps and the inferred pseudotime indicated strong temporal information encoded in the time labels. Otherwise, a close-to-zero correlation coefficient suggested that the known time labels did not preserve any coherent temporal information.

We considered three pseudotime inference algorithms: diffusion pseudotime (DPT) [15], TSCAN[14] and a simple MDS based method that can be scaled up for very large scRNA-seq datasets (see below). We found that for eleven real temporal scRNA-seq datasets that were examined in this study, the mean Kendall rank correlation coefficients between the known time labels and the inferred pseudotime was 0.39 (DPT: 0.35, TSCSN: 0.38, MDS: 0.43), suggesting a relatively strong correlation between the time labels and the underlying gene expression patterns (Supplementary Figure 21a).

Correlation between the time labels and pseudotime on highly asynchronized and random temporal scRNA-seq datasets

It is also possible that there exists highly asynchronized and random temporal scRNA-seq datasets, where there is little or no correlation between the known time labels and the underlying gene expression pattern. To investigate the correlation between time labels and pseudotime for these types of data, we simulated temporal scRNA-seq datasets where time labels were either uniformly sampled (*uniform cell sampling*) or the sampling process was independent of time points (*mixed cell sampling*) (see Supplementary Note 2). Supplementary Figures 21c and 21d illustrated the sampling probability p_{rt} for uniform and mixed cell sampling models. Not surprisingly, the mean correlation coefficients between time labels and pseudotime were close to zero (0.026 and 0.044 for uniform and mixed cell sampling), which were significantly lower than the sequential cell sampling where the sampling time was positively correlated with the developmental speed (mean Kendall's tau 0.258), as well as the correlation coefficients computed from the real datasets (Supplementary Figures 21a and 21b).

Thus, when the correlation between time labels and inferred pseudotime is close-to-zero (e.g. < 0.15), we recommend the use of more generic methods such as t-SNE or diffusion map to visualize the scRNA-seq data. Alternatively, TCM also provides an option to construct the pseudotime labels and the use of pseudotime labels, instead of the known time labels, to visualize the temporal scRNA-seq data. We found that for uniform and mixed cell sampling models, the pseudotime labels reflected developmental speed better than the original time labels (Supplementary Figure 21f and 21i), and the results were comparable with the generic methods such as t-SNE and diffusion map (Supplementary Figure 21l and 21m). We also found that for random and highly asynchronized temporal scRNA-seq data (random and mixed cell sampling), using pseudotime labels there was a significantly better performance of lineage discovery and the

separation of cell lineages from the last pseudotime points in our simulation studies (Supplementary Figures 21n and 21o).

Constructing pseudotime labels based on Multidimensional Scaling (MDS)

We have developed a simple MDS based pseudotime inference method that can be scaled up for very large scRNA-seq datasets, and have comparable performance with the published pseudotime inference methods such as DPT and TSCAN, as measured by the correlation coefficient between the known time labels and inferred pseudotime on real datasets (Supplementary Figure 21a).

First, an MDS (Multidimensional Scaling) algorithm was performed on the log transformed and standardized read count data, followed by the scaling of each low dimensional vectors to $[-1,1]$. Then, the cells were ordered by the average absolute values of the scaled low dimensional representation. The pseudotime labels were assigned by splitting the cells into equal parts (the number of pseudotime time points). For scRNA-seq datasets that were small or of moderate size (e.g. <2,000 cells), the R's *cmdscale* function was used for MDS. For large scRNA-seq datasets, we adopted a sampling-based fast MDS approximation algorithm[16]. TCM uses the function *pseudotime.table* for the construction of pseudotime labels.

Supplementary Note 7

Application of TCM on large scale scRNA-seq datasets

As large scale scRNA-seq datasets become more common[17], it is critical for the analysis tools to be sufficiently efficient to tackle such big datasets. TCM provides an option of using *stochastic variational inference (SVI)*, as an alternative to the batch optimization (Supplementary Note 4), for the visualization of large scale temporal scRNA-seq datasets. SVI performs stochastic optimization on the objective function used in the mean field variational Bayesian (VB) inference[9,18]. The stochastic variational technique is a general method that can address big data issues for many graphic model families.

At each iteration of SVI, the algorithm had an estimate of the global variational parameter (for example, the cell-wise metagene coefficient $\boldsymbol{v}_m$). It sampled single data (cell) uniformly from the data and computed the intermediate global parameter, that is, the next value if the data set contained S replicates of the sampled point. It then set the new estimate of the global parameter to be a weighted average of the previous estimate and the intermediate parameter. The iteration converged to a local optimum of the ELBO. For the detailed theory behind SVI, we recommend Hoffman et al. pioneering studies usingSVI[18].

Compared with the batch optimization method, the SVI method reduced the running time from ~7,000 seconds to ~1,100 seconds for a synthetic dataset with 10,000 genes and 10,000 cells, and is faster than the generic dimension reduction method such as diffusion map (*diffuse* in R *diffusionMap* package) and t-SNE (*Rtsne* in R *Rtsne* package) (Supplementary Figures 19b and 19f). Moreover, we also found that TCM with SVI optimization achieved a comparable performance for the separation of lineages as TCM with batch optimization (Supplementary Figures 19c and 19d). We believe that a fast SVI-based TCM

will broaden the application of TCM for large scale scRNA-seq datasets.

Supplementary Note 8

Inferring the developmental trajectories from TCM representations

TCM was primarily designed as a dimension reduction tool to visualize temporal scRNA-seq datasets. It has been shown that the developmental trajectories can be inferred based upon low dimensional representations, by using techniques such as minimum spanning tree (MST) connecting the individual cells or cell clusters[5,14,19-22], shortest paths connecting the progenitor and differentiated cells[11-13], principal curves[23,24], or correlation of low dimensional cellular representations[15,25].

Similarly, we have developed an empirical method to infer the developmental trajectory based upon the 2D representations of cellular topology from TCM. Specifically, for an active prototype (a prototype that has at least one mapped cell) on time t layer, we identified the most similar active prototypes at time $t-1$ layer as the parental prototype, and the most similar active prototypes at time $t+1$ layer as the children prototype. The distance between prototypes was computed as the Euclidean distance between their estimated metagene coefficients. The trajectories were drawn between the current prototype and its parental or children prototype as the shortest paths between them. The trajectory inference was implemented as the function *trajectory()* in the TCM package.

We found that this empirical method was able to recover the simulated lineages under three cell differentiation models (sequential, uniform and mixed cell sampling) (Supplementary Figures 22a-22c), while diffusion pseudotime (DPT) and TSCAN were unable to separate closely related lineages (e.g. simulated lineages L2 and L3 for DPT) (Supplementary Figures 22d-22g). We have also systematically evaluated the performance of trajectory inference between TCM (both sequential cell sampling using time labels and uniform cell sampling using pseudotime labels), DPT and TSCAN, under 2, 3, 5, 7, and 10 simulated

lineages ($N = 2{,}000, M = 500$). We found that TCM using sequential cell sampling (TCM(S)) followed by trajectory inference had significantly better performance on the estimation of single cell branches, while TCM using uniform cell sampling (TCM(U)) had similar performance with DPT when two or three simulated lineages were present. However, when there were five or more lineages present in the data, TCM had significantly better performance than either DPT or TSCAN regarding the definition of the lineage branches (Supplementary Figure 22h).

It should be noted that this trajectory inference method assumes that each active prototype at time t layer must have an immediate parental prototype at the $t-1$ layer (except when $t=1$) and an immediate children prototype at the $t+1$ layer (except when $t=T$). If for a certain lineage, the parental or children cell populations are absent in the cell populations with time label $t-1$ or $t+1$, the trajectory inference may generate incorrect results.

In this case, for temporal scRNA-seq datasets where each time point does not properly cover all the possible lineages, pseudotime labels need to be used to ensure that all possible lineages are properly covered at each pseudotime label, as we have demonstrated using pseudotime labels for simulated temporal scRNA-seq datasets of uniform and mixed cell sampling models (Supplementary Figures 22b, 22c and 22h). The other option is to merge the neighboring time labels (i.e., reducing the total time points) and ensure the parental/children prototypes are well represented in the neighboring time point.

We applied the trajectory inference method to the Loh et al. dataset on hESC differentiation after merging the D2.25 somitomere cells with D2 cells. The inferred trajectory generally reflected the bifurcation of paraxial and lateral mesodermal lineages (Supplementary Figure 22i), while DPT and TSCAN were unable to reveal a clear diversification of the two mesodermal lineages (Supplementary Figure 5a and 5d).

Supplementary References

1. Pierson, E. & Yau, C. ZIFA: Dimensionality reduction for zero-inflated single-cell gene expression analysis. *Genome Biol.* **16,** 241 (2015).
2. Kharchenko, P. V., Silberstein, L. & Scadden, D. T. Bayesian approach to single-cell differential expression analysis. *Nat Methods* (2014). doi:10.1038/nmeth.2967
3. Gong, W. *et al.* Dpath software reveals hierarchical haemato-endothelial lineages of Etv2 progenitors based on single-cell transcriptome analysis. *Nat Commun* **8,** 14362 (2017).
4. Klein, A. M. *et al.* Droplet barcoding for single-cell transcriptomics applied to embryonic stem cells. *Cell* **161,** 1187–1201 (2015).
5. Grün, D. *et al.* De Novo Prediction of Stem Cell Identity using Single-Cell Transcriptome Data. *Cell Stem Cell* **19,** 266–277 (2016).
6. Grün, D. *et al.* Single-cell messenger RNA sequencing reveals rare intestinal cell types. *Nature* (2015). doi:10.1038/nature14966
7. Wilson, N. K. *et al.* Combined Single-Cell Functional and Gene Expression Analysis Resolves Heterogeneity within Stem Cell Populations. *Cell Stem Cell* (2015). doi:10.1016/j.stem.2015.04.004
8. Bishop, C. M. *Pattern Recognition and Machine Learning*. (Springer, 2006).
9. Jordan, M. I., Ghahramani, Z., Jaakkola, T. S. & Saul, L. K. An introduction to variational methods for graphical models. *Mach Learn* **37,** 183–233 (1999).
10. Wang, C. & Blei, D. M. Variational inference in nonconjugate models. *The Journal of Machine Learning Research* **14,** 1005–1031 (2013).
11. Pollen, A. A. *et al.* Low-coverage single-cell mRNA sequencing reveals cellular heterogeneity and activated signaling pathways in developing cerebral cortex. *Nat. Biotechnol.* (2014). doi:10.1038/nbt.2967
12. Wilson, N. K. *et al.* Combined Single-Cell Functional and Gene Expression Analysis Resolves Heterogeneity within Stem Cell Populations. *Cell Stem Cell* **16,** 712–724 (2015).
13. Buettner, F. *et al.* Computational analysis of cell-to-cell heterogeneity in single-cell RNA-sequencing data reveals hidden subpopulations of cells. *Nat. Biotechnol.* **33,** 155–160 (2015).
14. Ji, Z. & Ji, H. TSCAN: Pseudo-time reconstruction and evaluation in single-cell RNA-seq analysis. *Nucleic Acids Res* (2016). doi:10.1093/nar/gkw430
15. Haghverdi, L., Büttner, M., Wolf, F. A., Buettner, F. & Theis, F. J. Diffusion pseudotime robustly reconstructs lineage branching. *Nat Methods* **13,** 845–848 (2016).
16. Yang, T., Liu, J., McMillan, L. & Wang, W. A fast approximation to multidimensional scaling. in (2006).
17. Regev, A. *et al.* The Human Cell Atlas. (2017). doi:10.1101/121202
18. Hoffman, M. D., Blei, D. M., Wang, C. & Paisley, J. Stochastic variational inference. *The Journal of Machine Learning Research* **14,** 1303–1347 (2013).

19. Street, K. *et al.* Slingshot: Cell lineage and pseudotime inference for single-cell transcriptomics. *bioRxiv* 128843 (2017). doi:10.1101/128843
20. Qiu, X. *et al.* Single-cell mRNA quantification and differential analysis with Census. *Nat Methods* (2017). doi:10.1038/nmeth.4150
21. Trapnell, C. *et al.* The dynamics and regulators of cell fate decisions are revealed by pseudotemporal ordering of single cells. *Nat. Biotechnol.* **32,** 381–386 (2014).
22. Shin, J. *et al.* Single-Cell RNA-Seq with Waterfall Reveals Molecular Cascades underlying Adult Neurogenesis. *Stem Cell* 1–14 (2015). doi:10.1016/j.stem.2015.07.013
23. Campbell, K., Ponting, C. P. & Webber, C. Laplacian eigenmaps and principal curves for high resolution pseudotemporal ordering of single-cell RNA-seq profiles. *bioRxiv* 027219 (2015). doi:10.1101/027219
24. Petropoulos, S. *et al.* Single-Cell RNA-Seq Reveals Lineage and X Chromosome Dynamics in Human Preimplantation Embryos. *Cell* **165,** 1012–1026 (2016).
25. Lönnberg, T. *et al. Temporal mixture modelling of single-cell RNA-seq data resolves a CD4+ T cell fate bifurcation*. (2016). doi:10.1101/074971
